# Multifunctional Curcumin-Inspired 3,5-Diarylidene-4-Piperidones: Design, Synthesis, Biological Evaluation and Computational Mechanistic Studies

**DOI:** 10.3390/ph19060935

**Published:** 2026-06-13

**Authors:** Angel K. Nkosi, Adel S. Girgis, Ahmed Samir, Mohamed A. Morsy, Amira M. Shaban, Walid Fayad, Ahmed A. F. Soliman, Christine T. Williams, Shogo Mori, Leena Khanna, Guido F. Verbeck, Siva S. Panda

**Affiliations:** 1 Department of Chemistry and Biochemistry, Augusta University, Augusta, GA 30912, USA; 2 Department of Pesticide Chemistry, National Research Centre, Giza 12622, Egypt; 3 Microbiology Department, Faculty of Veterinary Medicine, Cairo University, Cairo 12211, Egypt; 4 Al-Azhar Virology Research Center, Faculty of Medicine, Al-Azhar University, Cairo 71524, Egypt; 5 Botany and Microbiology Department, Faculty of Science, Beni-Suef University, Beni-Suef 62511, Egypt; 6 Drug Bioassay-Cell Culture Laboratory, Pharmacognosy Department, National Research Centre, Giza 12622, Egypt; 7 University School of Basic & Applied Sciences, Guru Gobind Singh Indraprastha University, New Delhi 110078, India; 8 Department of Biochemistry and Molecular Biology, Augusta University, Augusta, GA 30912, USA

**Keywords:** 4-piperidones, antimicrobial, *Staphylococcus aureus*, biofilm, efflux pump, gyrase, cancer, molecular modeling

## Abstract

**Background/Objectives:** Antimicrobial resistance and bacterial persistence underscore the need to develop new chemotypes with multifunctional antibacterial mechanisms. This study aimed to design, synthesize, and evaluate curcumin-inspired 3,5-diarylidene-4-piperidones as versatile small molecules exhibiting antibacterial, antibiofilm, anti-efflux, DNA gyrase-inhibitory, and antiproliferative properties. **Methods:** A targeted series of triazole-conjugated 3,5-diarylidene-4-piperidones was synthesized through copper-catalyzed azide-alkyne cycloaddition click chemistry and subsequently characterized using standard spectroscopic techniques. The compounds were assessed for antibacterial activity against *Staphylococcus aureus*, *Enterococcus faecalis*, and *Escherichia coli*. Selected active compounds underwent further evaluation for DNA gyrase inhibition, antibiofilm activity against multidrug-resistant *S. aureus* ATCC 33591, ethidium bromide accumulation, and antiproliferative effects on HCT116 and MCF7 cancer cells, with RPE1 cells serving as a control to evaluate cytotoxicity in normal cells. Additionally, computational studies, including QSAR analysis and molecular docking, were conducted to bolster structure–activity relationships and provide mechanistic insights. **Results:** Several derivatives demonstrated selective antibacterial activity against Gram-positive bacteria, particularly *S. aureus*, while exhibiting limited or no efficacy against *E. coli*. Compounds **7n** and **7l** emerged as the most potent against *S. aureus*, with minimum inhibitory concentrations (MICs) of 7.8 and 8.2 μM, respectively. Notably, compound **7l** inhibited *S. aureus* DNA gyrase supercoiling, displaying an IC_50_ of 3.20 μM, comparable to ciprofloxacin. Compound **7e** exhibited the strongest antibiofilm activity against multidrug-resistant *S. aureus*, whereas compound **7a** resulted in the highest accumulation of ethidium bromide, indicating robust anti-efflux activity. Antiproliferative assays revealed that select halogenated derivatives were effective against HCT116 and MCF7 cells, while the most promising antibacterial compounds exhibited minimal cytotoxicity toward RPE1 cells. Quantitative structure–activity relationship (QSAR) and docking studies supported the observed structure–activity relationships and suggested potential interactions with the ATPase binding site of DNA gyrase B. **Conclusions:** Triazole-conjugated 3,5-diarylidene-4-piperidones are promising multifunctional scaffolds with selective anti-*S. aureus* activity, antibiofilm and anti-efflux properties, and, for compound **7l**, potent DNA gyrase inhibition. These findings support further optimization of this chemotype as a platform for developing antibacterial agents with polymechanistic activity.

## 1. Introduction

Antimicrobial resistance (AMR) is one of the most serious global health threats of the 21st century, eroding decades of progress in infectious disease control and modern medicine [1,2,3]. The impact of AMR is especially severe in hospital settings, where multidrug-resistant pathogens threaten the care of immunocompromised patients, including those undergoing cancer treatment [4,5]. Among key bacterial species, *Staphylococcus aureus* stands out for its high adaptability, virulence, and rapid development of resistance [6,7]. The widespread rise in methicillin-resistant *S. aureus* (MRSA) has greatly limited treatment options, leading to higher death rates, longer hospital stays, and increasing healthcare costs [8,9,10,11].

The impact of AMR is especially significant in oncology. Infection-related complications continue to be a major cause of illness and death in cancer patients, where broad-spectrum antibiotics are commonly used to treat febrile neutropenia and other treatment-related infections [12]. A large number of deaths among critically ill cancer patients have been linked to hospital-acquired infections, most of which involve multidrug-resistant organisms. As a result, the decreasing effectiveness of current antibiotics presents an increasing threat to the success of modern cancer treatment strategies [13,14].

Although antibiotic misuse in healthcare and agriculture is a well-known driver of AMR, increasing evidence indicates that non-antibiotic chemical pressures also shape bacterial resistance [15]. Chemotherapeutic agents can indirectly promote AMR by increasing susceptibility to infection, prolonging hospitalization, and necessitating repeated antibiotic exposure. More importantly, several anticancer drugs have direct antibacterial activity, exerting selective pressure on bacterial populations and thereby promoting the development of resistance. For example, cisplatin inhibits bacterial growth by inducing DNA crosslinking, and bacterial adaptation to this damage can enhance DNA repair, leading to cross-resistance to other DNA-targeting agents such as fluoroquinolones [16,17]. Similarly, methotrexate, an antifolate structurally similar to trimethoprim, has been shown to drive the evolution of resistance and horizontal gene transfer in bacteria, thereby co-selecting multidrug-resistant strains [18]. Anthracyclines, although derived from *Streptomyces* species and thus inherently antimicrobial, can also induce expression of bacterial efflux pumps, thereby conferring resistance to structurally distinct antibiotics [19,20].

On the other hand, some anticancer agents have been shown to restore or enhance antibiotic activity by disrupting bacterial efflux systems or boosting membrane permeability. This dual function reveals an underutilized therapeutic opportunity: the targeted development of multifunctional small molecules that can act as direct antibacterial agents while also modulating resistance mechanisms [21,22,23]. This strategy is especially promising for high-priority Gram-positive pathogens such as *S. aureus*, in which efflux, biofilm formation, and target protection are key factors in persistence and multidrug resistance [24,25,26].

In this context, curcumin and its synthetic analogs have emerged as promising candidates for the development of multifunctional drugs. Curcumin, a naturally occurring polyphenol derived from *Curcuma longa*, exhibits a range of biological activities, including anti-inflammatory, anticancer, and antimicrobial effects, and has been shown to be effective against *S. aureus* [27,28,29]. However, its therapeutic application is hindered by poor chemical stability, low bioavailability, and modest potency. These challenges have spurred ongoing medicinal chemistry efforts to develop curcumin-inspired scaffolds that preserve biological activity while enhancing physicochemical and pharmacological properties. Among these scaffolds, 3,5-diarylidene-4-piperidones stand out as a particularly attractive chemotype. These curcumin mimics maintain essential conjugated pharmacophores while offering improved chemical stability, structural modularity, and ease of synthesis. Previous studies have demonstrated that 3,5-diarylidene-4-piperidones exhibit a wide range of bioactivities, including anti-inflammatory [30] and antiproliferative [31] activities. As shown in earlier reports, the integration of heterocyclic moieties, such as 1,2,3-triazoles, into curcumin frameworks has proven to be an effective strategy for enhancing stability, solubility, and biological activity. This approach has led to the development of potent anticancer agents with improved metabolic profiles. The resulting triazole-incorporated curcumin analogs underscore the significance of molecular hybridization techniques in creating multifunctional scaffolds that simultaneously modulate multiple biological targets [32].

In addition, the extended conjugation and cation-tolerant frameworks of 3,5-diarylidene-4-piperidones suggest possible interactions with bacterial membranes, efflux systems, and critical intracellular targets, including DNA gyrase, a validated antibacterial target in *S. aureus* [33].

Given the urgent clinical need for new treatments effective against *S. aureus*, especially multidrug-resistant strains common in cancer settings, and the growing recognition that anticancer-inspired molecules can modulate bacterial resistance mechanisms, exploring multifunctional curcumin mimics is both timely and strategically important. Compounds that combine direct antibacterial action, efflux pump modulation, biofilm disruption, and acceptable mammalian cell selectivity may offer a new approach to address infection-related complications in cancer treatment and broader clinical applications.

In this study, we outline the design, synthesis, and comprehensive biological testing of a series of curcumin-inspired 3,5-diarylidene-4-piperidones, with a focus on their antimicrobial activity against *S*. *aureus*. The compounds were methodically examined for antibacterial activity against multidrug-resistant strains, inhibition of biofilm formation, modulation of efflux pumps using ethidium bromide accumulation assays, and inhibition of DNA gyrase. Simultaneously, their antiproliferative effects on cancer cell lines and their selectivity for normal cells were evaluated, with support from computational mechanistic studies. Overall, this work highlights 3,5-diarylidene-4-piperidones as promising multifunctional small-molecule candidates at the crossroads of antimicrobial and anticancer drug development.

## 2. Results

**Figure 1:**
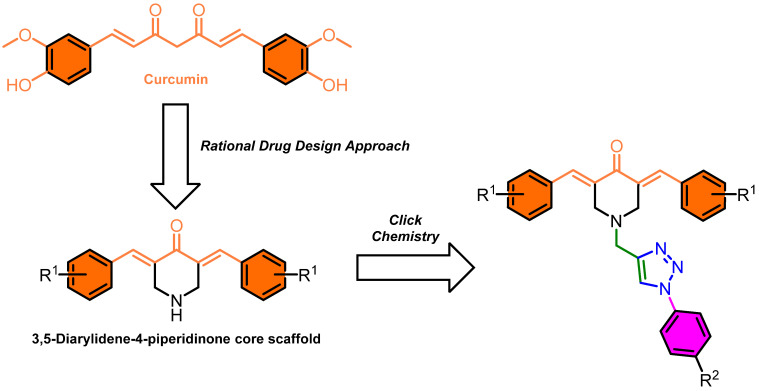
Design of the proposed compounds.

Guided by principles of rational drug design and the multi-target-directed ligand (MTDL) strategy, we developed a targeted series of compounds to address key challenges in antimicrobial drug development, especially resistance and limited therapeutic durability. The 3,5-diarylidene-4-piperidinone core, chosen for its curcumin-like properties and proven biological significance [30,31], was structurally modified using click chemistry to include a 1,2,3-triazole linker (Figure 1). This approach aimed to improve molecular stability, allow precise electronic and steric adjustments, and facilitate concurrent targeting of multiple bacterial components. By integrating these features into a single chemical structure, the compounds were expected to exhibit multifunctional antimicrobial activity, potentially reducing the risk of resistance and reducing reliance on combination therapies. The following sections outline the synthesis, biological testing, and mechanistic studies of this compound series, emphasizing structure–activity relationships and their importance for antibacterial activity against *S. aureus*.

### 2.1. -Synthesis and Characterization

**Scheme 1:**
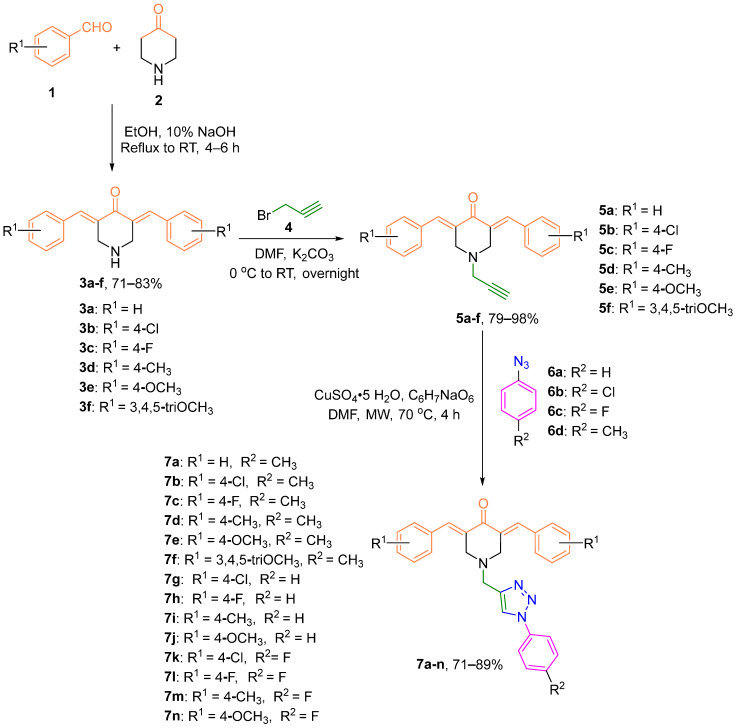
Synthetic route for compounds **7a**–**n**.

The target triazole-conjugated 3,5-diarylidene-4-piperidinones (**7a**–**n**) were synthesized using the route outlined in Scheme 1. Initially, 3,5-diarylidene-4-piperidinone intermediates (**3a**–**f**) were prepared through base-catalyzed aldol condensation of 4-piperidinone (**2**) with substituted aromatic aldehydes (**1**) following previously reported procedures [30,31]. The reactions afforded the desired conjugated intermediates in yields ranging from 71 to 83%.

Subsequently, selective *N*-propargylation of intermediates **3a**–**f** was achieved by treatment with propargyl bromide (**4**) in the presence of potassium carbonate (K_2_CO_3_) in dimethylformamide (DMF) at ambient temperature, affording the corresponding *N*-propargylated derivatives (**5a**–**f**) in yields of 79–95%. In parallel, substituted aryl azides (**6a**–**d**) were prepared using standard diazotization–azidation procedures.

The final compounds (**7a**–**n**) were obtained through copper-catalyzed azide–alkyne cycloaddition (CuAAC) between alkynes **5a**–**f** and aryl azides **6a**–**d**. Reaction conditions were optimized by evaluating different solvent systems, catalysts, reducing agents, and heating methods (Table 1). Among the tested conditions, the combination of CuSO_4_·5H_2_O and sodium D-isoascorbate in DMF under microwave irradiation produced the highest conversion. Microwave heating at 70 °C for 4 h afforded compounds **7a**–**n** in yields of up to 89%, whereas conventional heating provided lower yields.

All synthesized compounds were purified and characterized using standard spectroscopic methods, confirming the successful formation of the target triazole-linked 3,5-diarylidene-4-piperidinone derivatives.

### 2.2. Biological Studies

#### 2.2.1. Antibacterial Properties

The synthesized compounds **7a**–**n**, representing curcumin-inspired bis(enone)-containing 3,5-diarylidene-4-piperidone derivatives [34,35,36,37], were evaluated for antibacterial activity against *Staphylococcus aureus* (ATCC 25923), *Enterococcus faecalis* (ATCC 29212), and *Escherichia coli* (ATCC 25922) using established methodologies [37,38]. Ciprofloxacin (CIP), a clinically established fluoroquinolone antibacterial agent with broad-spectrum activity [39,40,41,42], was included as the reference compound. The antibacterial activity results are summarized in Table 2.

Several derivatives demonstrated measurable antibacterial activity against *S. aureus*. Compound **7n** (R^1^ = 4-OCH_3_, R^2^ = F) exhibited the highest potency with an MIC value of 7.8 μM, followed closely by compound **7l** (R^1^ = R^2^ = F, MIC = 8.2 μM). Compounds **7f** (R^1^ = 3,4,5-triOCH_3_, R^2^ = CH_3_) and **7a** (R^1^ = H, R^2^ = CH_3_) also showed strong antibacterial activity, with MIC values of 12.8 and 17.9 μM, respectively. Compound **7e** (R^1^ = 4-OCH_3_, R^2^ = CH_3_) retained moderate activity with an MIC value of 31.6 μM.

Against *E. faecalis*, antibacterial activity was generally reduced relative to that observed against *S. aureus*. Among the tested compounds, **7n** exhibited the strongest inhibitory effect, with an MIC of 250.7 μM. Most other derivatives exhibited moderate to weak activity.

No detectable antibacterial activity was observed against Gram-negative *E. coli* under the experimental conditions employed.

#### 2.2.2. DNA Gyrase Inhibition

Based on their antibacterial activity against *S. aureus*, compounds **7a**, **7e**, **7f**, **7l**, and **7n** were selected for evaluation of DNA gyrase inhibitory activity using a DNA supercoiling assay followed by agarose gel electrophoresis and densitometric analysis [43]. CIP, a clinically established DNA gyrase inhibitor [44], was included as the reference inhibitor.

**Figure 2:**
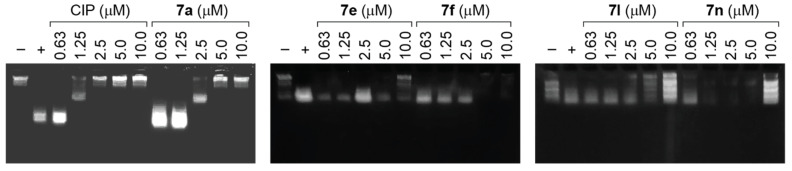
DNA gyrase (*S. aureus*) agarose gel electrophoresis of the tested compounds.

**Figure 3:**
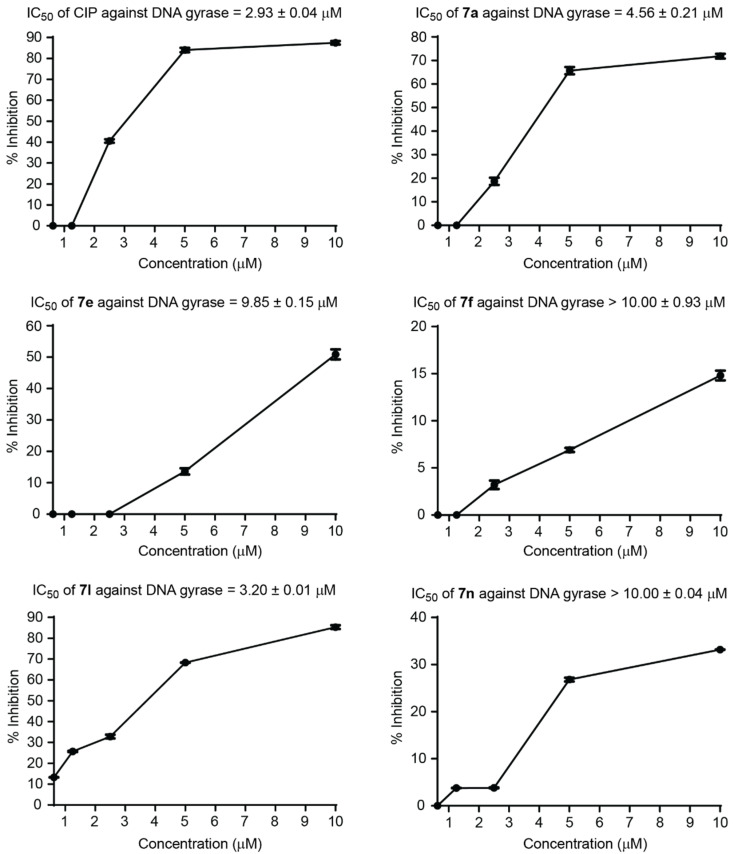
Dose–response curves of the tested compounds as DNA gyrase inhibitors.

As summarized in Table 3 and illustrated in Figure 2 and Figure 3, several compounds inhibited *S. aureus* DNA gyrase. Compound **7l** exhibited the strongest inhibitory activity with an IC_50_ value of 3.20 μM. Compound **7a** also showed potent inhibition (IC_50_ = 4.56 μM), while compound **7e** demonstrated moderate activity (IC_50_ = 9.85 μM). In contrast, compounds **7f** and **7n** showed limited inhibitory effects under the assay conditions (IC_50_ > 10 μM). The inhibitory activities of the tested compounds were compared with ciprofloxacin (IC_50_ = 2.93 μM), and the complete results are presented in Table 3.

#### 2.2.3. Antibiofilm Properties

Biofilms are organized clusters of microorganisms that form an aggregate biomass within a self-produced slimy matrix [45]. Certain bacterial strains, notably *S. aureus*, can transition from a solitary, planktonic lifestyle to a structured biofilm network [46,47]. Within this network, bacterial cells adhere closely to one another, creating a physical barrier that limits antibiotic penetration and significantly increases antimicrobial tolerance [48,49].

The most promising antibacterial compounds (**7a**, **7e**, **7f**, **7l**, and **7n**) were evaluated for their ability to inhibit biofilm formation using a standard antibiofilm assay [50]. Testing was performed against the multidrug-resistant (MDR) *S. aureus* strain ATCC 33591, and activity was compared with that of ciprofloxacin (CIP) at concentrations corresponding to double the observed MIC values (Table 4).

As summarized in Table 4, several compounds inhibited biofilm formation to varying extents. Compound **7e** demonstrated the strongest antibiofilm activity, resulting in 20.2% biofilm detected under the assay conditions. Compounds **7l** and **7n** also reduced biofilm formation, with biofilm detection values of 39.1% and 54.4%, respectively. Compounds **7a** and **7f** showed lower antibiofilm effects.

#### 2.2.4. Anti-Efflux Properties

Microbial efflux is a physiological mechanism that enables microorganisms to actively extrude antibiotics from the intracellular environment. As a primary bacterial defense strategy, efflux pumps play a significant role in the development of MDR, particularly in pathogens such as *S. aureus*. Consequently, extensive research has focused on optimizing efflux pump inhibitors to develop novel antibacterial agents or to restore the efficacy of existing clinical antibiotics [51,52,53].

Efflux pump inhibitory activity was evaluated by measuring intracellular accumulation of ethidium bromide (EtBr) using a semiautomated fluorometric assay [54]. The selected compounds (**7a**, **7e**, **7f**, **7l**, and **7n**) were tested against the multidrug-resistant (MDR) *S. aureus* strain ATCC 33591 at concentrations corresponding to one-half of their MIC values. Carbonyl cyanide *m*-chlorophenylhydrazone (CCCP) was used as the reference inhibitor.

**Figure 4:**
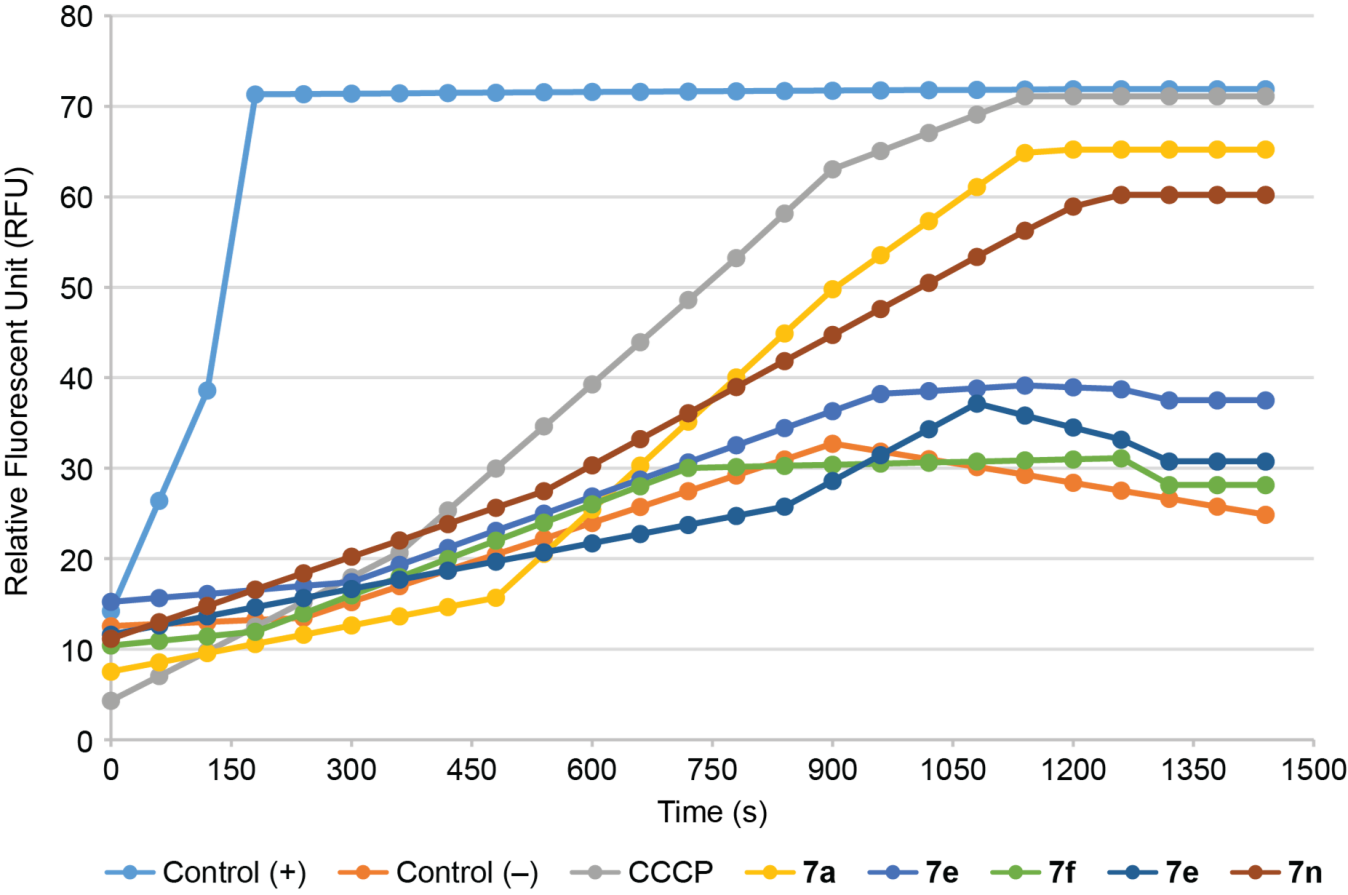
Anti-efflux pumping properties of the tested compounds [control (+) is the heat-killed bacteria, control (−) is the untreated bacteria, and CCCP is carbonyl cyanide *m*-chlorophenylhydrazone, “reference anti-efflux drug”].

As summarized in Table 5 and Figure 4, the tested compounds exhibited varying degrees of efflux pump inhibitory activity. Compound **7a** showed the strongest response, with 90.7% efflux-inhibitory activity, approaching the activity of CCCP (98.9%). Compound **7n** also demonstrated strong activity with 83.7% inhibition. Compounds **7e**, **7l**, and **7f** exhibited moderate inhibitory effects, with activity values of 52.2%, 42.8%, and 39.1%, respectively.

#### 2.2.5. Antiproliferation Properties

The antiproliferative activity of compounds **7a**–**n** was evaluated against HCT116 (colon) and MCF7 (breast) cancer cell lines using the MTT assay [55]. Sunitinib [56,57] and 5-fluorouracil [58] were included as reference compounds. The results are summarized in Table 6 and Supplementary Figures S76–S78.

HCT116

Several derivatives demonstrated measurable antiproliferative activity against HCT116 cells. Compound **7k** (R^1^ = 4-Cl, R^2^ = F) exhibited the strongest activity with an IC_50_ value of 5.26 μM. Compounds **7c** (R^1^ = 4-F, R^2^ = CH_3_) and **7h** (R^1^ = 4-F, R^2^ = H) also showed potent activity, with IC_50_ values of 6.01 and 6.06 μM, respectively. Compounds **7g** (R^1^ = 4-Cl, R^2^ = H) and **7b** (R^1^ = 4-Cl, R^2^ = CH_3_) demonstrated moderate antiproliferative effects, with IC_50_ values of 8.60 and 9.59 μM, respectively.

MCF7

Antiproliferative activity against MCF7 cells was more limited. Among the tested compounds, only **7b** and **7c** demonstrated measurable activity, with IC_50_ values of 8.70 and 7.73 μM, respectively.

RPE1

Cytotoxicity toward normal cells was evaluated using the RPE1 cell line (retinal pigment epithelium). All compounds exhibiting antiproliferative activity maintained IC_50_ values above the tested concentration threshold in RPE1 cells. In addition, the antibacterial lead compounds (**7a**, **7e**, **7f**, **7l**, and **7n**) displayed minimal cytotoxicity toward RPE1 cells and showed limited antiproliferative activity against HCT116 and MCF7 cells.

### 2.3. Computational Results

Computational chemistry techniques have garnered increasing interest within medicinal chemistry due to their utility in optimizing and designing bioactive agents. A variety of in silico methodologies have demonstrated significant efficacy in identifying and refining biological hits and leads. Key among these approaches are quantitative structure–activity relationship (QSAR) modeling, pharmacophore analysis, molecular docking, and molecular dynamics (MD) simulations [59,60,61].

#### 2.3.1. QSAR Analysis

A quantitative structure–activity relationship (QSAR) analysis was performed using CODESSA-Pro to investigate relationships between molecular descriptors and antibacterial activity against *S. aureus* (ATCC 25923) [61]. A dataset consisting of synthesized compounds with varying antibacterial potencies was used to generate predictive models based on the reciprocal MIC values (1/MIC).

**Figure 5:**
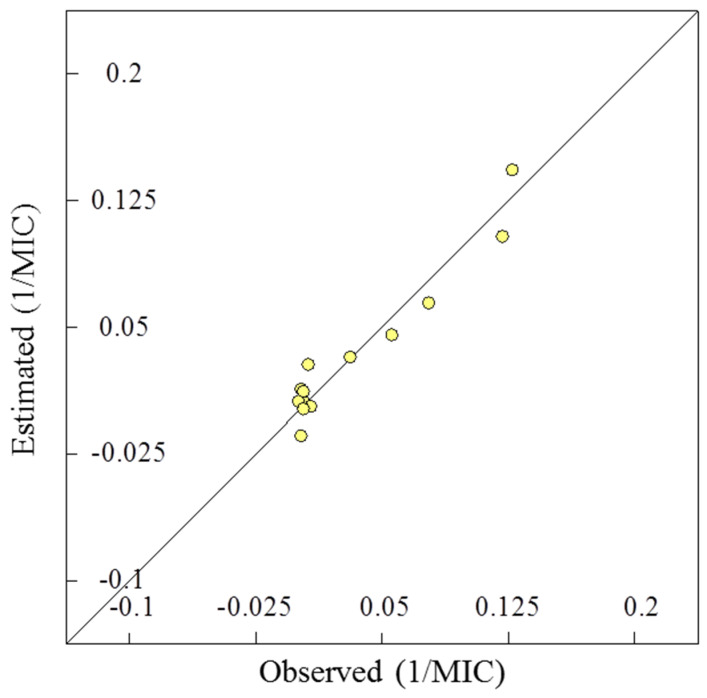
QSAR plot representing the observed versus predicted MIC values of the tested compounds.

A three-descriptor QSAR model was identified as the optimal model for describing antibacterial activity and demonstrated strong statistical performance with R^2^ = 0.931, R^2^cvOO = 0.830, and R^2^cvMO = 0.862. The model covered compounds with observed activity values ranging from 0.001 to 0.128 μM^−1^ (7.8–985.8 μM), while predicted values ranged from −0.014 to 0.143 μM^−1^ (−66.8–1458.8 μM) (Figure 5 and Supplementary Tables S1–S3).

The final model incorporated three descriptors: the minimum electron–nuclear (e–n) attraction of a C–N bond, the π–π bond order, and the mean information content. Descriptor definitions and corresponding equations are provided in Equations (3) and (4) [62].

Internal validation was performed to evaluate model robustness. Leave-one-out (LOO) and leave-many-out (LMO) cross-validation results were consistent with the original model statistics (R^2^ = 0.931, R^2^cvOO = 0.830, R^2^cvMO = 0.862). Additional statistical parameters included s^2^ = 0.0002 and F = 45.097, supporting the reliability of the generated model.

#### 2.3.2. Molecular Dynamics Simulation Results

Molecular docking simulations were performed to evaluate the interaction of selected compounds with *S. aureus* DNA gyrase B. Docking calculations were conducted using the CDOCKER protocol implemented in Discovery Studio 4.1 employing a CHARMM-based MD sampling algorithm [63].

The receptor model used was the crystallographic structure of *S. aureus* DNA gyrase B co-crystallized with an ATP-competitive inhibitor (PDB ID: 3TTZ; resolution 1.63 Å) [64,65]. Protein preparation included removal of crystallographic water molecules, correction of missing residues, addition of hydrogen atoms, and energy minimization using the CHARMM force field with MMFF94 partial charges.

The ATPase binding site was defined using the coordinates of the co-crystallized ligand (X = 0.220, Y = 3.716, Z = 24.113) with a spherical region of radius 9.208 Å [66]. Re-docking of the native ligand reproduced the crystallographic binding orientation and yielded an RMS gradient value of 0.091.

**Figure 6:**
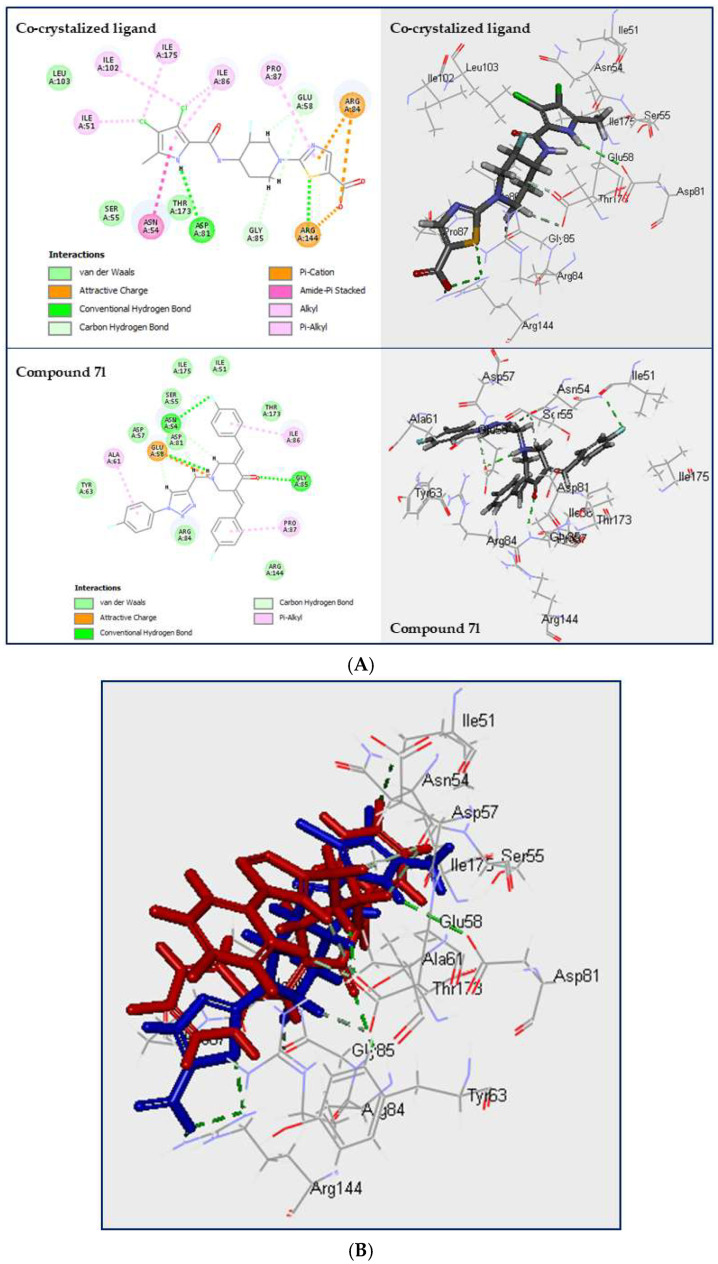
(**A**). 2D- and 3D-docking poses of co-crystallized ligand and **7l** in the active site of PDB ID: 3TTZ. (**B**). Alignment of docked co-crystallized ligand (blue) with compound **7l** (red) in the active site of PDB ID: 3TTZ.

Based on antibacterial and DNA gyrase inhibition results, compounds **7a**, **7e**, **7f**, **7l**, and **7n** were selected for docking analysis. Docking scores and interaction profiles are summarized in Table 7, and representative binding poses are shown in Figure 6 and Figure S81.

All evaluated compounds occupied the ATPase-binding pocket of DNA gyrase B and formed combinations of hydrogen-bonding, π–alkyl, π–cation, and hydrophobic interactions with residues including Asn54, Gly85, Pro87, Ile86, Arg84, Arg144, Glu58, and Ile102.

Compound **7l** showed hydrogen-bonding interactions with Asn54 and Gly85, an electrostatic interaction with Glu58, and multiple π–alkyl contacts with Pro87 and Ile86. Compound **7a** exhibited primarily π–alkyl and π–cation interactions with Pro87, Ile86, and Arg84. Compound **7e** formed interactions involving Arg144, Asn54, Arg84, Pro87, and Ile86. Compounds **7f** and **7n** also occupied the ATPase pocket and exhibited hydrogen-bonding and hydrophobic interactions with residues including Arg144, Gly85, Pro87, and Ile86.

#### 2.3.3. Molecular Dynamics SimulationResults

**Figure 7:**
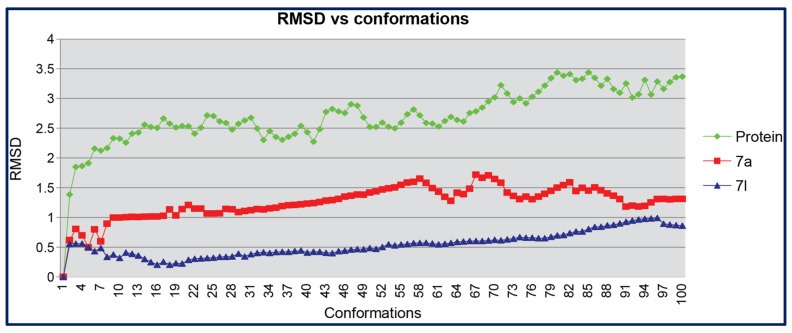
RMSD of the protein backbone and compounds **7a** and **7l** in the protein PDB ID: 3TTZ.

**Figure 8:**
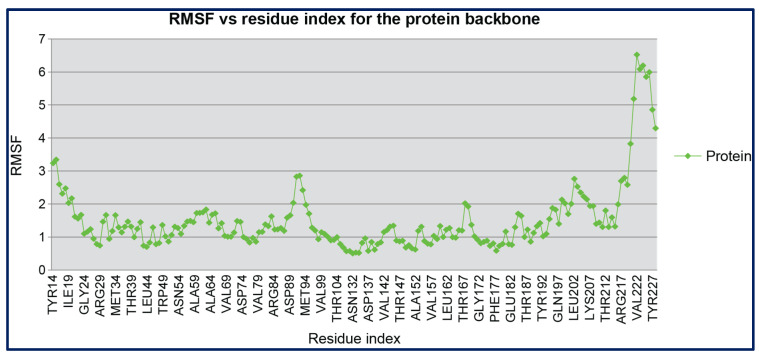

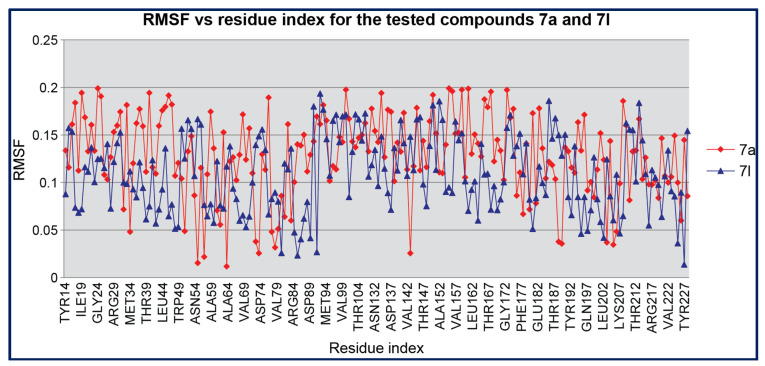
RMSF of the (**above**) protein backbone and (**below**) compounds **7a** and **7l** in the protein PDB ID: 3TTZ.

Molecular dynamics (MD) simulations were performed to evaluate the dynamic behavior of selected protein–ligand complexes and to further assess the stability of the docking poses. Compounds **7a** and **7l** were selected based on their antibacterial, biochemical, and docking performance. Simulations were conducted in Discovery Studio 4.1 using the top-ranked docking conformations as starting structures. RMSD and RMSF analyses were performed using trajectories obtained over a 100 ns simulation period (Figure 7 and Figure 8; Supplementary Tables S4–S9) [55].

RMSD

Root-mean-square deviation (RMSD) analysis was used to monitor conformational changes in the ligand and protein backbone throughout the simulation. Compound **7a** exhibited RMSD values ranging from 0.498 to 1.721 Å, while compound **7l** displayed RMSD values of 0.992 to 1.721 Å. In comparison, the protein backbone RMSD ranged from 1.384 to 3.440 Å.

The average RMSD values calculated for compounds **7a** and **7l** were 1.251 and 0.540 Å, respectively, whereas the average RMSD of the protein backbone was 2.702 Å (Figure 7).

RMSF

Root-mean-square fluctuation (RMSF) analysis was performed to examine local flexibility during the simulation period (Figure 8; Supplementary Tables S7–S9). Compound **7a** exhibited RMSF values ranging from 0.012 to 0.199 Å, and compound **7l** showed values of 0.014 to 0.194 Å, whereas the protein backbone exhibited fluctuations between 0.505 and 6.196 Å.

Residues associated with binding interactions exhibited lower fluctuations in ligand-bound simulations. Arg144 displayed RMSF values of 0.026 Å for compound **7a** and 0.148 Å for compound **7l** compared with 1.213 Å for the protein backbone. Similarly, Ile86 and Pro87 exhibited reduced fluctuations in the ligand-bound complexes relative to the corresponding protein backbone values.

#### 2.3.4. Physicochemical Properties (In Silico Results)

The physicochemical properties and preliminary drug-likeness parameters for compounds **7a**–**n** were calculated using SwissADME [67] and are summarized in Table 8, with curcumin included as a reference compound. Table 9 provides a visual representation of the physicochemical space [68] occupied by the most active synthesized compounds, with additional data in the Supporting Information.

The calculated parameters included molecular weight, hydrogen-bond acceptors and donors, lipophilicity (logP), rotatable bond count, topological polar surface area (TPSA), molar refractivity (MR), and predicted blood–brain barrier (BBB) permeability.

Most compounds exhibited hydrogen-bond acceptor counts and hydrogen-bond donor values within commonly applied drug-likeness ranges. Molecular weights varied across the series, with several compounds exceeding 500 Da. Calculated lipophilicity values generally clustered toward the higher end of the evaluated range. Rotatable bond counts remained moderate across most derivatives, while TPSA values varied depending on the substitution pattern. Molar refractivity values were generally higher than those calculated for curcumin.

Predicted BBB permeation profiles differed among compounds, with some derivatives predicted to cross the BBB and others predicted to remain non-permeant.

#### 2.3.5. In Silico Toxicity Assessment

Various chemotherapy drugs exhibit substantial renal, liver, cardiac, and nervous system toxicities [69]. For instance, anticancer drugs such as cisplatin, cytarabine, vinca alkaloids, and anthracyclines exert their toxic effects by inducing coagulation necrosis, myocarditis, cholestasis, peripheral paresthesia, thrombosis, and other mechanisms [69,70,71]. Toxicity prediction of the synthesized compounds was performed using ProTox 3.0 to obtain preliminary safety-related parameters. The predicted toxicity profiles are summarized in Table 10 [72].

The majority of the synthesized hybrids were predicted to exhibit a probability greater than 50% of lacking hepatotoxicity, nephrotoxicity, and cardiotoxicity. In addition, all compounds were predicted to have relatively high estimated lethal dose (LD_50_) values of approximately 500 mg/kg.

## 3. Discussion

### 3.1. Discussion of Synthetic Strategy

The synthetic strategy employed in this study enabled the efficient generation of a structurally diverse library of triazole-conjugated 3,5-diarylidene-4-piperidinones **7a**–**n** while preserving the core diarylidene-4-piperidinone framework. Selection of this scaffold was guided by its established utility as a curcumin-inspired platform, favorable synthetic accessibility, and opportunities for structural diversification [30,31]. The modular sequence of aldol condensation, *N*-propargylation, and CuAAC-mediated conjugation provided a practical route for introducing multiple electronic and steric variations without substantially altering the central pharmacophore.

Formation of intermediates **3a**–**f** through aldol condensation proceeded efficiently under mild basic conditions, demonstrating good tolerance toward both electron-donating and electron-withdrawing substituents on the aromatic rings. Subsequent *N*-propargylation generated versatile alkyne-containing intermediates suitable for late-stage diversification, thereby enabling the rapid assembly of analogs while minimizing the need for synthetic redesign of earlier intermediates.

Optimization of the CuAAC reaction highlighted the importance of both solvent selection and energy input for efficient triazole formation. Among the conditions evaluated, DMF combined with CuSO_4_·5H_2_O and sodium D-isoascorbate under microwave irradiation provided the highest conversion and product yields. The marked improvement observed under microwave conditions, compared with conventional heating, suggests enhanced reaction efficiency and faster generation of the catalytically active Cu(I) species. The progressive increase in yield observed with reduced reaction time under microwave-assisted conditions indicates improved reaction kinetics and reduced formation of undesired side products.

Incorporation of the 1,2,3-triazole linker is an advantageous molecular design feature because this heterocycle is recognized as a chemically stable and metabolically robust bioisostere that can influence molecular polarity, electronic distribution, and intermolecular interaction profiles. In addition to serving as a rigid linker, the triazole moiety provides additional heteroatoms that can participate in hydrogen-bonding interactions and may modulate physicochemical and biological properties.

### 3.2. Biological Activity Discussion

#### 3.2.1. Discussion of Antibacterial Activity

Evaluation of the synthesized diarylidene-4-piperidone derivatives revealed selective antibacterial activity predominantly against Gram-positive organisms, with *S. aureus* displaying substantially greater susceptibility than *E. faecalis* and *E. coli*. This activity profile is consistent with previous reports describing antibacterial effects of curcumin-inspired bis(enone)-containing scaffolds and related 3,5-diarylidene-4-piperidones [34,35,36,37].

The most active derivatives, **7n** and **7l**, exhibited low micromolar activity against *S. aureus*, indicating that both aromatic substitution and triazole-linked modifications influence antibacterial performance. The absence of detectable activity against *E. coli* may reflect reduced penetration through the Gram-negative outer membrane and/or intrinsic resistance mechanisms that limit intracellular accumulation of these compounds.

Analysis of structure–activity relationships suggests that substitution patterns on the exocyclic ylidene rings strongly influence antibacterial potency. In general, methoxy substitution improved anti-*S. aureus* activity relative to methyl-substituted analogs, particularly when comparing compounds **7e**/**7f**/**7d** (MIC = 31.6, 12.8, and 269.7 μM, respectively) and **7n**/**7m** (MIC = 7.8 and 267.5 μM, respectively). These observations suggest that electron-donating methoxy groups may favor structural or physicochemical features associated with antibacterial activity.

Substitution at the triazole-linked aryl group also influenced activity. In several matched pairs, derivatives bearing a *p*-tolyl substituent exhibited greater antibacterial potency than their corresponding phenyl analogs, including **7b**/**7g**, **7c**/**7h**, and **7d**/**7i**. Although exceptions were observed, these results indicate that subtle steric and electronic differences introduced at the triazole substituent can substantially alter biological response.

#### 3.2.2. Discussion of DNA Gyrase Inhibition

Evaluation of selected antibacterial candidates revealed that inhibition of DNA gyrase contributes to the antibacterial activity of part of this compound series. Among the tested derivatives, compound **7l** displayed enzymatic potency approaching that of ciprofloxacin, indicating that structural modification of the diarylidene-4-piperidone scaffold can generate compounds capable of effectively interfering with bacterial DNA topology regulation.

However, the relationship between whole-cell antibacterial activity and enzymatic inhibition was not fully proportional across all compounds. Although compounds such as **7l** and **7a** showed strong inhibition in both assays, compounds including **7f** and **7n** retained antibacterial activity despite relatively weak inhibition of isolated DNA gyrase. These observations suggest that whole-cell efficacy is influenced by factors beyond target affinity alone.

Differences between antibacterial and biochemical assay outcomes may arise from variations in compound uptake, intracellular accumulation, membrane permeability, or differences between isolated enzyme systems and the more complex cellular environment. The retained antibacterial activity observed for compounds with modest gyrase inhibition also supports the possibility that additional mechanisms contribute to the overall biological response.

#### 3.2.3. Interpretation of Antibiofilm Activity

The ability of selected derivatives to inhibit biofilm formation against MDR *S. aureus* demonstrates an additional functional dimension beyond direct antibacterial activity. Because biofilm formation is a key contributor to bacterial persistence and reduced antibiotic susceptibility, compounds that disrupt this process may offer advantages in combating resistant infections.

Among the tested derivatives, compound **7e** exhibited the strongest antibiofilm effect and slightly outperformed ciprofloxacin under the assay conditions employed. This observation suggests that structural modifications within the diarylidene-4-piperidone framework can substantially influence antibiofilm behavior independently of overall antibacterial potency. Although compounds **7l** and **7n** were less effective than **7e** at suppressing biofilm formation, they retained measurable activity and exhibited favorable antibacterial profiles.

Comparison across biological assays indicates that antibiofilm activity does not fully correlate with either planktonic antibacterial potency or DNA gyrase inhibition. This suggests that biofilm suppression may involve additional mechanisms beyond growth inhibition alone, potentially including interference with bacterial adhesion, extracellular matrix formation, or stress-adaptation pathways.

#### 3.2.4. Mechanistic Insights into Efflux Inhibition

Efflux-mediated resistance represents an important mechanism contributing to multidrug resistance in *S. aureus*, making inhibition of transporter activity an attractive complementary antibacterial strategy. Evaluation of intracellular ethidium bromide accumulation demonstrated that selected diarylidene-4-piperidone derivatives possess varying capacities to interfere with bacterial efflux function.

Among the tested compounds, **7a** displayed the strongest anti-efflux activity and produced intracellular accumulation levels approaching those of the reference inhibitor CCCP. Compound **7n** also exhibited substantial activity, whereas compounds **7e**, **7l**, and **7f** showed lower but measurable effects. These findings indicate that structural modifications within the series influence bacterial accumulation behavior and may contribute to differences observed in whole-cell antibacterial activity.

Comparison with antibacterial and DNA gyrase assays suggests that efflux inhibition does not directly parallel enzymatic inhibition or antibacterial potency across all derivatives. For example, compounds demonstrating strong anti-efflux activity were not necessarily the most potent DNA gyrase inhibitors, suggesting that multiple biological mechanisms contribute to the observed antibacterial responses.

#### 3.2.5. Discussion on Antiproliferation Activity

Evaluation of antiproliferative activity revealed that the biological response in this compound series depended on the substitution pattern and differed substantially from the antibacterial profile. While several derivatives displayed measurable anticancer activity, the most potent antibacterial compounds generally showed limited growth inhibition in cancer cells, suggesting partial divergence between antibacterial and antiproliferative determinants within the scaffold.

Against HCT116 cells, compounds containing halogen substitution on the exocyclic ylidene moiety exhibited the most favorable activity profile. In particular, derivatives containing fluorinated aromatic groups tended to demonstrate greater potency than closely related chloro analogs, as illustrated by compound pairs **7c**/**7b** and **7h**/**7g**. These observations suggest that electronic effects associated with halogen substitution may influence interactions relevant to antiproliferative activity.

In contrast, activity against MCF7 cells was more restricted, observed only for compounds **7b** and **7c**, indicating that structural requirements for activity may differ across tumor types. The relatively narrow activity profile suggests that these compounds do not function as broadly cytotoxic agents under the conditions tested.

Importantly, compounds exhibiting measurable antiproliferative activity retained favorable selectivity toward normal RPE1 cells. Likewise, the antibacterial lead compounds exhibited minimal toxicity in normal cells yet remained largely inactive in cancer assays. This separation between antibacterial and mammalian cell activity may be an advantageous property for future antibacterial optimization by reducing concerns about nonspecific cytotoxicity.

### 3.3. Computational Analysis and Interpretation

#### 3.3.1. QSAR-Based Structure–Activity Relationship Analysis

The QSAR analysis provided computational support for the experimentally observed antibacterial trends and identified molecular features associated with anti-*S. aureus* activity. The strong agreement between experimental and predicted activity values suggests that the selected descriptor set captures the relevant structural determinants governing the antibacterial response in this compound series.

Among the descriptors, the minimum electron–nuclear (e–n) attraction of the C–N bond contributed most strongly to the model, as indicated by its t-value. This descriptor reflects electrostatic stabilization within the molecular framework and shows a positive relationship with antibacterial activity in the 1/MIC model. Compounds with higher descriptor values generally exhibited improved predicted potency, as illustrated by compounds **7n** and **7g**.

The π–π bond order descriptor also emerged as an important contributor, reflecting differences in π-electron localization and electronic distribution across the conjugated scaffold. Its contribution is consistent with the experimentally observed SARs involving aromatic substitution on the ylidene moiety. Comparisons such as **7n** and **7k** suggest that increased values for this descriptor are associated with improved antibacterial potency.

In contrast, mean information content demonstrated a negative contribution to activity. This topological descriptor reflects molecular symmetry and structural complexity, indicating that increased structural complexity within the current chemical space may reduce antibacterial performance. The relationship observed between compounds **7c** and **7l** supports this trend.

Because the present analysis was performed on a focused dataset, internal validation was selected as the primary approach to assess model performance. The close agreement among R^2^, LOO, and LMO values, together with favorable statistical parameters, supports the model’s robustness within the investigated chemical space. Nevertheless, external validation on larger, more structurally diverse datasets will be necessary to establish broader predictive applicability.

#### 3.3.2. Molecular Docking Interpretation and Binding Mechanism

Molecular docking analysis provided a structural context for the experimentally observed DNA gyrase inhibition and supported the antibacterial SARs identified within this series. The ability of all selected compounds to occupy the ATPase binding region suggests that the diarylidene-4-piperidone scaffold is compatible with productive interaction within the DNA gyrase B active site.

Among the evaluated compounds, **7l** demonstrated the most favorable overall interaction profile and corresponded closely with its experimentally observed enzyme inhibition. The combination of hydrogen-bonding interactions with Asn54 and Gly85, electrostatic stabilization involving Glu58, and hydrophobic contacts with Pro87 and Ile86 may contribute to improved binding stability within the ATPase pocket. The observed spatial overlap with the co-crystallized ligand further supports a binding mode consistent with ATP-site occupancy.

Compound **7a** retained favorable enzymatic activity despite exhibiting a simpler interaction network dominated by hydrophobic contacts, suggesting that extensive hydrogen bonding may not be strictly required for measurable inhibition. In contrast, compound **7e** exhibited a mixed interaction profile combining polar and nonpolar contacts but showed lower experimental potency than **7l**, indicating that interaction geometry may influence functional outcomes.

Compounds **7f** and **7n** produced docking poses consistent with ATPase binding but showed weaker correspondence to the enzymatic inhibition results. This divergence suggests that favorable docking scores alone do not fully predict biological activity and that factors such as ligand orientation, protein dynamics, intracellular accumulation, membrane permeability, or additional cellular mechanisms may contribute to antibacterial performance.

#### 3.3.3. Dynamic Behavior and Stability Analysis of Protein–Ligand Complexes

MD simulations were performed to complement the docking analysis by evaluating whether the predicted binding modes remained stable under dynamic conditions. The overall RMSD and RMSF profiles indicate that the docked conformations remain stable throughout the simulation period for both evaluated compounds.

The relatively narrow RMSD distributions observed for compounds **7a** and **7l**, together with lower average ligand RMSD values compared with the protein backbone, indicate that both ligands maintained stable positioning within the DNA gyrase ATPase binding pocket over the 100 ns trajectory. Among the evaluated complexes, compound **7l** exhibited the lowest average RMSD, consistent with its favorable docking profile and experimentally observed inhibition of DNA gyrase.

RMSF analysis further supported these observations by demonstrating reduced mobility of residues involved in ligand binding. In particular, residues such as Arg144, Ile86, and Pro87 exhibited lower fluctuations in the ligand-bound state compared with the free protein backbone. These findings suggest that ligand binding contributes to stabilization of local regions of the ATPase binding pocket.

The agreement between docking and MD analyses strengthens confidence in the predicted binding orientations and indicates that the observed docking poses are not artifacts of static calculations alone. Nevertheless, MD simulations remain computational approximations and do not fully capture all biological variables, including long-timescale conformational transitions, intracellular conditions, and broader cellular effects.

#### 3.3.4. Physicochemical Profile and Drug-Likeness Interpretation

Computational evaluation of physicochemical and ADME-related properties provided preliminary insight into the developability of the synthesized triazole-conjugated 3,5-diarylidene-4-piperidone derivatives **7a**–**n** and complemented the experimental biological findings. Assessment of individual molecular descriptors together with visualization of physicochemical space enabled a broader understanding of how structural modifications influence both biological performance and drug-like characteristics.

Overall, the synthesized series occupied physicochemical space that is broadly compatible with small-molecule development while simultaneously revealing properties that may benefit from further optimization. Most derivatives maintained acceptable hydrogen-bonding characteristics, including favorable hydrogen-bond acceptor counts and the absence of hydrogen-bond donors across the series, features that may support membrane transport and molecular recognition.

Several compounds exceeded the conventional molecular weight threshold commonly associated with Lipinski’s Rule of Five. This increase is consistent with the expanded structural complexity introduced through triazole conjugation and aromatic extension. Although elevated molecular weight may affect oral absorption, such deviations are not uncommon among multifunctional small molecules and should be interpreted in the context of the overall physicochemical profile rather than as isolated limitations.

The calculated lipophilicity values generally clustered toward the upper end of the preferred range, indicating greater hydrophobicity than curcumin. Moderate-to-high lipophilicity may favor interactions with bacterial membranes and intracellular targets; however, excessive values could negatively affect aqueous solubility and broader pharmacokinetic behavior. Likewise, molecular flexibility remained generally controlled across the series, with rotatable bond counts remaining within ranges commonly associated with acceptable conformational adaptability.

Polarity and surface characteristics also contributed to differentiation among compounds. TPSA values for many derivatives remained within ranges often associated with passive permeability, whereas compounds with higher polarity may exhibit reduced diffusion potential. Increased molar refractivity values relative to curcumin reflect the greater aromaticity and conjugation introduced through triazole incorporation, which may enhance intermolecular interactions but could also affect absorption characteristics in larger analogs.

Visualization of physicochemical space provided an integrated framework for interpreting these molecular properties collectively rather than individually. The distribution of the most active compounds suggests that antibacterial activity was achieved within a region characterized by balanced lipophilicity, moderate flexibility, controlled polarity, and increased molecular complexity. These observations indicate that the current scaffold can maintain biological activity while remaining within a physicochemical window compatible with continued medicinal chemistry optimization.

Predicted blood–brain barrier permeability further differentiated the series. Compounds predicted to exhibit limited BBB penetration may be advantageous for systemic antibacterial applications by reducing central nervous system exposure, whereas BBB-permeant analogs may require additional optimization depending on the intended therapeutic objective.

Taken together, the physicochemical analysis supports the continued development of selected diarylidene-4-piperidone derivatives and provides guidance for future structure-based optimization to balance biological potency, molecular complexity, and developability.

#### 3.3.5. Discussion of Toxicity and Safety Profile

A preliminary in silico toxicity assessment was conducted to complement the biological evaluation and provide an early indication of developability across the synthesized series. Because toxicity remains a major limitation in therapeutic development, early computational profiling may help prioritize compounds for further investigation.

The predicted toxicity profiles suggest that several of the synthesized derivatives may possess acceptable preliminary safety characteristics, particularly with respect to liver, kidney, and cardiac toxicity endpoints. The estimated LD_50_ values also indicate moderate predicted acute toxicity within the limitations of the computational model.

These observations are encouraging when considered alongside the experimental finding that the most active antibacterial compounds exhibited minimal cytotoxicity toward normal RPE1 cells. Together, these results support continued evaluation of selected derivatives while recognizing that computational toxicity prediction does not substitute for experimental toxicological assessment.

## 4. Materials and Methods

### 4.1. Chemical Synthesis

All chemicals were used as purchased from Fisher (Hampton, NH, USA) and Merck (Burlington, MA, USA), without further purification. Thin-layer chromatography (TLC) was conducted to evaluate the purity of the compounds using a 3:7 ratio of ethyl acetate (99.0%, Fisher, Hampton, NH, USA) to n-hexane (99.0%, Fisher, Hampton, NH, USA). The retardation factor (*R*_f_) values ranged from 0.5 to 0.7. TLC was performed on pre-coated silica gel aluminum plates (Merck 60 F254, MilliporeSigma, Burlington, MA, USA). The spots were visualized using iodine vapors (Fisher, Hampton, NH, USA) or by UV irradiation (Thermo Fisher Scientific, Waltham, MA, USA) at 254 nm. Melting points were determined using a capillary-point apparatus equipped with a digital thermometer (Thermo Fisher Scientific, Waltham, MA, USA). NMR spectra were recorded in CDCl_3_ on a Bruker spectrometer operating at 500 MHz for ^1^H (with TMS as an internal standard) and 125 MHz for ^13^C using the NMR facility at the Department of Chemistry and Biochemistry, Augusta University, Augusta, GA, USA. Infrared (IR) spectra (KBr, cm^−1^) were recorded on a Nicolet iS5 spectrophotometer (Thermo Fisher Scientific, Waltham, MA, USA) at the Department of Chemistry and Biochemistry, Augusta University, Augusta, GA, USA. Mass spectrometry (MS) was measured using an Agilent Technologies 6545 Q-TOF LC/MS (Agilent Technologies, Santa Clara, CA, USA). High-Performance Liquid Chromatography (HPLC) Agilent 1100 Series with a 1260 Infinity II LC was used to obtain the HPLC-MS data.

***The general procedure for the synthesis of* 5a–f** [73,74]

3,5-Diarylidene-4-piperidone (**3a**–**f,** 1 eq.), propargyl bromide (**4**, 1.1 eq.), and potassium carbonate were combined in a round-bottom flask equipped with a stir bar, utilizing DMF (10 mL) as the solvent. The mixture was cooled in an ice bath and stirred overnight, allowing the temperature to gradually rise from 0 °C to room temperature. For the work-up, the reaction was quenched with ice-cold deionized water until a precipitate appeared. The yellow solids were collected through vacuum filtration and rinsed with deionized water. The resulting compounds (**5a**–**f**) exhibited high purity; however, NMR analysis revealed the presence of residual DMF, which was used as the reaction solvent.

**3,5-Bis((*E*)-benzylidene)-1-(prop-2-yn-1-yl)piperidin-4-one (5a)**

Yellow solid, m. p.: 135-137 °C, yield: 87%. IR: ν_max_/cm^−1^; 3260, 1675, 1610, 1585, 1485, 1442, 1296, 1252, 1213, 1177, 1092, 992, 940, 763, 690, 659, 535, 518; ^1^H-NMR (500 MHz, CDCl_3_) δ: 7.88 (s, 2H, 2 olefinic CH), 7.49 (d, *J* = 8 Hz, 4H), 7.46 (d, *J* = 10 Hz, 4H), 7.43 (d, *J* = 8, 2H), 3.97 (s, 4H, 2 NCH_2_), 3.57 (s, 2H), 2.41 (s, 1H, alkyne CH); ^13^C-NMR (125 MHz, CDCl_3_) δ: 186.7 (C=O), 136.6 (olefinic C), 135.2, 133.0, 130.4, 129.1, 128.6, 74.6 (alkyne C), 53.4 (2 NCH_2_), 46.5 (>NCH_2_).

**3,5-Bis((*E*)-4-chlorobenzylidene)-1-(prop-2-yn-1-yl)piperidin-4-one (5b)**

Yellow solid, m. p.: 108–110 °C, yield: 95%. IR: ν_max_/cm^−1^; 3279, 1651, 1611, 1585, 1488, 1441, 1388, 1253, 1180, 1093, 1026, 975, 916, 817, 762, 688, 659, 515; ^1^H-NMR (500 MHz, CDCl_3_) δ: 7.79 (s, 2H, 2 olefinic CH), 7.45 (d, *J* = 8.5 Hz, 4H), 7.37 (d, *J* = 8.0 Hz, 4H), 3.91 (s, 4H, 2 NCH_2_), 3.57 (s, 2H), 2.41 (s, 1H, alkyne CH); ^13^C-NMR (125 MHz, CDCl_3_) δ: 186.3 (C=O), 135.4 (olefinic C), 135.2, 133.5, 133.27, 131.6, 128.9, 74.8 (alkyne C), 53.3 (2 NCH_2_), 46.6 (>NCH_2_).

**3,5-Bis((*E*)-4-fluorobenzylidene)-1-(prop-2-yn-1-yl)piperidin-4-one (5c)**

Yellow solid, m. p.: 146–148 °C, yield: 98%. IR: ν_max_/cm^−1^; 3278, 1669, 1614, 1574, 1505, 1414, 1309, 1299, 1255, 1223, 1184, 1156, 1104, 1088, 1000, 919, 829, 799, 777, 761, 699, 629, 579, 529, 495, 409; ^1^H-NMR (500 MHz, CDCl_3_) δ: 7.82 (s, 2H, 2 olefinic CH), 7.47–7.42 (m, 4H, ArH), 7.20–7.15 (m, 4H, ArH), 3.93 (s, 4H, 2 NCH_2_), 3.58 (s, 2H, >NCH_2_-), 2.41 (s, 1H, alkyne CH); ^13^C-NMR (125 MHz, CDCl_3_) δ: 186.5 (C=O), 162.99 (d, *J_C–F_* = 249.4 Hz, 2 × Ar–C–F, benzylidene fluorophenyl rings), 135.5 (olefinic C), 132.4 (Ar-CH=), 131.3 (d, *J_C–F_* = 8.2 Hz), 115.9 (d, *J_C–F_* = 21.7 Hz, Ar-CH), 74.8 (alkyne C), 53.3 (2 NCH_2_), 46.6 (>NCH_2_).

**3,5-Bis((*E*)-4-methylbenzylidene)-1-(prop-2-yn-1-yl)piperidin-4-one (5d)**

Yellow solid, m. p.: 133–135 °C, yield: 82%. IR: ν_max_/cm^−1^; 3231, 1672, 1601, 1573, 1508, 1446, 1411, 1315, 1295, 1253, 1195, 1175, 1128, 1117, 1084, 1001, 976, 929, 918, 817, 794, 692, 661, 514, 491, 413, 404; ^1^H-NMR (500 MHz, CDCl_3_) δ: 7.85 (s, 2H, 2 olefinic CH), 7.37 (d, *J* = 8 Hz, 4H), 7.29 (d, *J* = 8 Hz, 4H), 3.96 (s, 4H, 2 NCH_2_), 3.57 (s, 2H), 2.44 (s, 6H), 2.41 (s, 1H, alkyne CH); ^13^C-NMR (125 MHz, CDCl_3_) δ: 186.7 (C=O), 139.4, 136.6 (olefinic C), 132.4, 132.2, 130.5, 129.3, 74.5 (alkyne C), 53.5 (2 NCH_2_), 46.5 (>NCH_2_), 21.4 (CH_3_).

**3,5-Bis((*E*)-4-methoxybenzylidene)-1-(prop-2-yn-1-yl)piperidin-4-one (5e)**

Yellow solid, m. p.: 143–151 °C, yield: 79%. IR: ν_max_/cm^−1^; 3261, 1670, 1597, 1577, 1507, 1456, 1440, 1301, 1250, 1167, 1142, 1116, 1084, 1022, 1001, 976, 947, 928, 915, 828, 687, 673, 551, 524; ^1^H-NMR (500 MHz, CDCl_3_) δ: 7.82 (s, 2H, 2 olefinic CH), 7.43 (d, *J* = 8.5 Hz, 4H), 7.01 (d, *J* = 8.5 Hz, 4H), 3.96 (s, 4H, 2 NCH_2_), 3.90 (s, 6H, 2 OCH_3_), 3.59 (s, 2H, 2 NCH_2_), 2.41 (s, 1H, alkyne CH); ^13^C-NMR (125 MHz, CDCl_3_) δ: 186.6 (C=O), 136.2 (olefinic C), 132.4, 132.3, 131.1, 127.9, 114.1, 114.0, 74.5 (alkyne C), 55.3 (OCH_3_), 53.5 (2 NCH_2_), 46.6 (>NCH_2_).

**1-(** **Prop-2-yn-1-yl)-3,5-bis((*E*)-3,4,5-trimethoxybenzylidene)piperidin-4-one (5f)**

Yellow solid, m. p.: 157–159 °C, yield: 90%. IR: ν_max_/cm^−1^; 3285, 2934, 1661, 1606, 1580, 1502, 1445, 1417, 1387, 1336, 1303, 1243, 1209, 1151, 1119, 1021, 1006, 983, 940, 915, 836, 818, 662, 636, 616, 552, 517, 445; ^1^H-NMR (500 MHz, CDCl_3_) δ:, 7.79 (s, 2H, 2 olefinic CH), 6.69 (s, 4H), 3.98 (s, 4H, 2 NCH_2_), 3.94 (s, 6H, 2 OCH_3_), 3.93 (s, 12H, 4 OCH_3_), 3.58 (s, 2H), 2.38 (s, 1H, alkyne CH); ^13^C-NMR (125 MHz, CDCl_3_) δ: 186.4 (C=O), 153.1, 139.2, 136.8 (olefinic C), 132.3, 130.6, 108.0, 107.9, 74.4 (alkyne C), 61.0 (OCH_3_), 56.2 (OCH_3_), 53.6 (2 NCH_2_), 46.6 (>NCH_2_).

***The general procedure for the synthesis of* 6a–d** [74]

The sodium nitrite solution (1.33 eq. dissolved in 15 mL of DI water) and sodium azide solution (1.42 eq. in 15 mL of DI water) were prepared. Aniline derivative (1 eq.) was mixed with approximately 150 mL of a 1:1 water and hydrochloric acid solution at 0 °C and stirred. The sodium nitrite solution was added dropwise over 5 min at 0–5 °C, and the mixture was stirred for 40 min at the same temperature. Next, the sodium azide solution was added, and the mixture was stirred overnight as it gradually warmed from 0 °C to room temperature. For compounds **6a**–**d**, the workup involved diluting with 60 mL of water, then performing three extractions with 100 mL of ethyl acetate each. The combined organic layers were dried over anhydrous sodium sulfate. Finally, the solvent was removed via rotary evaporation, yielding the concentrated compounds **6a**–**d**.

***The general procedure for the synthesis of* 7a–n**

A solution of the respective alkyne derivative **5a**–**f** (1 eq.) in 3 mL of DMF was placed in a dried heavy-walled Pyrex tube containing a small stir bar at room temperature. Copper sulfate pentahydrate (0.3 eq.), sodium L-ascorbate (0.4 eq.), and the corresponding aryl azide **6a**–**d** (4 eq.) were added at room temperature. The reaction mixture was exposed to microwave irradiation (20 W) at 70 °C for 4 h, monitored by TLC. The mixture was allowed to cool, then quenched with ice-cold DI water (10 mL). Dark brown or dark yellow precipitates were isolated after vacuum filtration using DI water as the wash, and the desired compounds were obtained in pure form by column chromatography (20% ethyl acetate in hexanes or 1% methanol in methylene chloride).

**3,5-Di((*E*)-benzylidene)-1-((1-(*p*-tolyl)-1*H*-1,2,3-triazol-4-yl)methyl)piperidin-4-one (7a)**

Yellow solid, m. p.: 175–177 °C, yield: 85%. IR: ν_max_/cm^−1^; 1673, 1613, 1582, 1518, 1488, 1444, 1318, 1259, 1230, 1174, 1089; ^1^H-NMR (500 MHz, CDCl_3_) δ: 7.85 (s, 2H, 2 olefinic CH), 7.72 (s, 1H, triazole CH), 7.47–7.42 (m, 8H), 7.37 (d, *J* = 6.5 Hz, 2H), 7.29–7.27 (m, 4H), 4.00 (s, 4H, 2 NCH_2_), 3.98 (s, 2H, >NCH_2_), 2.42 (s, 3H, CH_3_); ^13^C-NMR (125 MHz, CDCl_3_) δ: 187.40 (C=O), 144.79, 138.9, 135.5 (olefinic C), 134.6, 132.6, 133.2, 130.2, 130.1, 120.8, 120.4, 54.6 (2 NCH_2_), 52.3 (>NCH_2_), 21.0 (CH_3_); HRMS: *m*/*z* for C_29_H_26_N_4_O [M + H]^+^ Calcd.:447.2179, Found: 447.2236.

**3,5-Bis((*E*)-4-chlorobenzylidene)-1-((1-(*p*-tolyl)-1*H*-1,2,3-triazol-4-yl)methyl)piperidin-4-one (7b)**

Yellow solid, m. p.: 183–185 °C, yield: 79%. IR: ν_max_/cm^−1^; 1575, 1489, 1264, 1240, 1180, 1094, 1072, 1044, 1010, 999, 989, 931, 814, 785, 768, 527, 516, 502, 457, 408; ^1^H-NMR (500 MHz, CDCl_3_) δ: 7.75 (s, 2H, 2 olefinic CH), 7.72 (s, 1H, triazole CH), 7.46 (d, *J* = 8.5 Hz, 2H), 7.38 (d, *J* = 8.5 Hz, 4H), 7.32–7.28 (m, 4H), 7.27–7.25 (m, 2H), 3.95 (s, 2H), 3.92 (s, 4H, 2 NCH_2_), 2.40 (s, 3H, CH_3_); ^13^C-NMR (125 MHz, CDCl_3_) δ: 187.0 (C=O), 144.6, 143.2, 139.0, 135.5 (olefinic C), 135.3, 133.4, 133.3, 131.6, 130.2, 129.0, 120.8, 120.4, 54.4 (2 NCH_2_), 52.3 (>NCH_2_), 21.1 (CH_3_); HRMS: *m*/*z* for C_29_H_24_Cl_2_N_4_O [M + H]^+^ Calcd.: 515.1399, Found: 515.1354.

**3,5-Bis((*E*)-4-fluorobenzylidene)-1-((1-(*p*-tolyl)-1*H*-1,2,3-triazol-4-yl)methyl)piperidin-4-one (7c)**

Yellow solid, m. p.: 175–177 °C, yield: 72%. IR: ν_max_/cm^−1^; 1677, 1616, 1598, 1520, 1506, 1458, 1414, 1341, 1331, 1316, 1293, 1269, 1256, 1219, 1160, 1104, 1094, 1001, 991, 922, 910, 833, 780, 769, 538, 520, 494, 408; ^1^H-NMR (500 MHz, CDCl_3_) δ: 7.77 (s, 2H, 2 olefinic CH), 7.74 (s, 1H, triazole CH), 7.47 (d, *J* = 8.5 Hz, 2H), 7.39–7.36 (m, 4H), 7.28–7.25 (m, 2H), 7.10 (t, *J* = 8.5 Hz, 4H), 4.00 (s, 2H, >NCH_2_), 3.93 (s, 4H, 2 NCH_2_), 2.40 (s, 3H, CH_3_); ^13^C-NMR (125 MHz, CDCl_3_) δ: 187.1 (C=O), 163.0 (d, *J_C–F_* = 248.8 Hz, 2 ×x Ar–C–F, benzylidene fluorophenyl rings), 144.7, 139.0, 135.6 (olefinic C), 134.7, 132.4, 131.2, 130.2, 120.9, 120.4, 115.9 (d, *J_C–F_* = 21.2 Hz, Ar-CH), 54.5 (2 NCH_2_), 52.4 (>NCH_2_), 21.0 (CH_3_); ^19^F-NMR (470.6 MHz, CDCl_3_) δ: −110.5; HRMS: *m*/*z* for C_29_H_24_F_2_N_4_O [M + H]^+^ Calcd.: 483.1991, Found: 483.1969.

**3,5-Bis((*E*)-4-methylbenzylidene)-1-((1-(*p*-tolyl)-1*H*-1,2,3-triazol-4-yl)methyl)pipaeridin-4-one (7d)**

Yellow solid, m. p.: 155–157 °C, yield: 86%. IR: ν_max_/cm^−1^; 1670, 1605, 1510, 1321, 1263, 1175, 1045, 989, 815, 516, 487; ^1^H-NMR (500 MHz, CDCl_3_) δ: 7.80 (s, 2H, 2 olefinic CH), 7.67 (s, 1H, triazole CH), 7.42 (d, *J* = 8.0 Hz, 2H), 7.28 (d, *J* = 8.0 Hz, 4H), 7.24 (d, *J* = 8.0 Hz, 2H), 7.20 (d, *J* = 7.5 Hz, 4H), 4.00 (s, 6H, 2 NCH_2_ + >NCH_2_), 2.39 (s, 3H, CH_3_), 2.35 (s, 6H, 2 CH_3_); ^13^C-NMR (125 MHz, CDCl_3_) δ: 187.5 (C=O), 145.1, 140.9, 139.5, 138.8, 136.8 (olefinic C), 134.7, 132.4, 130.5, 130.1, 129.4, 120.8, 120.4, 54.6 (2 NCH_2_), 52.3 (>NCH_2_), 21.4 (2 CH_3_), 21.1 (CH_3_); HRMS: *m*/*z* for C_31_H_30_N_4_O [M + H]^+^ Calcd.: 475.2492, Found: 475.2502.

**3,5-** **Bis((*E*)-4-methoxybenzylidene)-1-((1-(*p*-tolyl)-1*H*-1,2,3-triazol-4-yl)methyl)piperidin-4-one (7e)**

Yellow solid, m. p.: 187–189 °C, yield: 78%. IR: ν_max_/cm^−1^; 1662, 1592, 1558, 1520, 1508, 1457, 1441, 1421, 1331, 1318, 1270, 1248, 1230, 1169, 1151, 1121, 1090, 1045, 1025, 999, 930, 835, 822, 532, 497, 468; ^1^H-NMR (500 MHz, CDCl_3_) δ: 7.81 (s, 2H, 2 olefinic CH), 7.70 (s, 1H, triazole CH), 7.44 (d, *J* = 8.5 Hz, 2H), 7.36 (d, *J* = 8.5 Hz, 4H), 7.26–7.24 (m, 2H), 6.93 (d, *J* = 8.5 Hz, 4H), 3.97 (s, 6H, 2 OCH_3_), 3.82 (s, 6H, 2 NCH_2_ + >NCH_2_), 2.39 (s, 3H, CH_3_); ^13^C-NMR (125 MHz, CDCl_3_) δ: 187.0 (C=O), 160.3, 145.1, 138.8, 136.3 (olefinic C), 132.4, 131.3, 130.2, 128.0, 126.4, 120.8, 120.4, 116.9, 114.3, 55.3 (2 OCH_3_), 54.7 (2 NCH_2_), 52.4 (>NCH_2_), 21.1 (CH_3_); HRMS: *m*/*z* for C_31_H_30_N_4_O_3_ [M + H]^+^ Calcd.: 507.239067, Found: 507.2352.

**1-((1-(*p*-Tolyl)-1*H*-1,2,3-triazol-4-yl)methyl)-3,5-bis((*E*)-3,4,5-trimethoxybenzylidene)piperidin-4-one (7f)**

Yellow solid, m. p.: 245–247 °C, yield: 75%. IR: ν_max_/cm^−1^; 2939, 1656, 1639, 1572, 1525, 1496, 1467, 1451, 1408, 1330, 1309, 1229, 1185, 1125, 1099, 1050, 1006, 992, 920, 868, 814, 803, 777, 723, 692; ^1^H-NMR (500 MHz, CDCl_3_) δ: 8.22 (s, 1H, triazole CH), 8.06 (s, 2H, 2 olefinic CH), 7.71 (d, *J* = 7.5 Hz, 2H), 7.46 (d, *J* = 7.5 Hz, 2H), 7.22 (s, 4H), 3.97 (s, 6H, 2 OCH_3_), 3.94 (s, 12H, 4 OCH_3_), 3.93 (s, 4H, 2 NCH_2_), 3.91 (s, 2H, >NCH_2_), 2.56 (s, 3H, CH_3_); ^13^C-NMR (125 MHz, CDCl_3_) δ: 183.6 (C=O), 153.2, 152.9, 139.9, 139.0, 136.6 (olefinic C), 130.5, 130.3, 123.1, 120.6, 120.3, 108.1, 107.6, 106.8, 65.8 (4 OCH_3_), 60.9 (2 OCH_3_), 56.3 (2 NCH_2_ + >NCH_2_), 21.1 (CH_3_); HRMS: *m*/*z* for C_35_H_38_N_4_O_7_ [M + H]^+^ Calcd.: 627.2813, Found: 627.2586.

**3,5-Bis((*E*)-4-chlorobenzylidene)-1-((1-phenyl-1*H*-1,2,3-triazol-4-yl)methyl)piperidin-4-one (7g)**

Yellow solid, m. p.: 177–179 °C, yield: 78% (46 mg). IR: ν_max_/cm^−1^; 1702, 1672, 1610, 1579, 1489, 1408, 1288, 1250, 1229, 1189, 1180, 1094, 1010, 1000, 975, 914, 823, 810, 793, 689, 680, 531, 515, 411; ^1^H-NMR (500 MHz, CDCl_3_) δ: 7.69 (s, 3H, 2 olefinic CH + triazole CH), 7.68 (d, *J* = 7.5 Hz, 2H), 7.59 (d, *J* = 7.5 Hz, 2H), 7.50–7.38 (m, 5H), 7.31 (d, *J* = 8.0 Hz, 4H), 3.96 (s, 2H, >NCH_2_), 3.93 (s, 4H, 2 NCH_2_); ^13^C-NMR (125 MHz, CDCl_3_) δ: 186.9 (C=O), 135.6 (olefinic C), 135.3, 133.4, 133.3, 131.6, 129.8, 129.2, 129.0, 128.8, 120.8, 120.6, 120.5, 54.5 (2 NCH_2_), 52.3 (>NCH_2_); HRMS: *m*/*z* for C_28_H_22_Cl_2_N_4_O [M + H]^+^ Calcd.: 501.1243, Found: 502.1906.

**3,5-Bis((*E*)-4-fluorobenzylidene)-1-((1-phenyl-1*H*-1,2,3-triazol-4-yl)methyl)piperidin-4-one (7h)**

Yellow solid, m. p.: 181–183 °C, yield: 89%. IR: ν_max_/cm^−1^; 1673, 1614, 1597, 1579, 1504, 1304, 1286, 1218, 1198, 1154, 1049, 999, 921, 910, 848, 827, 809, 763, 693, 539, 530, 494, 422, 408; ^1^H-NMR (500 MHz, CDCl_3_) δ: 7.78 (s, 3H, 2 olefinic CH + triazole CH), 7.60 (d, *J* = 7.5 Hz, 2H), 7.48 (t, *J* = 7.5 Hz, 2H), 7.40-7.36 (m, 4H), 7.10 (t, *J* = 8.5 Hz, 4H), 3.97 (s, 2H, >NCH_2_), 3.94 (s, 4H, 2 NCH_2_); ^13^C-NMR (125 MHz, CDCl_3_) δ: 187.0 (C=O), 163.0 (d, *J_C–F_* = 250.0 Hz, 2 × Ar–C–F, benzylidene fluorophenyl rings), 145.0, 137.0, 135.6 (olefinic C), 132.4, 131.3, 129.8, 128.8, 120.9, 120.5, 115.9 (d, *J_C–F_* = 21.2 Hz, Ar-CH), 54.5 (2 NCH_2_), 52.4 (>NCH_2_); ^19^F-NMR (470.6 MHz, CDCl_3_) δ: −110.5; HRMS: *m*/*z* for C_28_H_22_F_2_N_4_O [M + H]^+^ Calcd.: 469.1834, Found: 469.1708.

**3,5-Bis((*E*)-4-methylbenzylidene)-1-((1-phenyl-1*H*-1,2,3-triazol-4-yl)methyl)piperidin-4-one (7i)**

Yellow solid, m. p.: 174–176 °C, yield: 79% (266 mg). IR: ν_max_/cm^−1^; 1670, 1600, 1573, 1508, 1320, 1293, 1248, 1231, 1178, 1090, 1040, 1021, 999, 975, 926, 840, 814, 757, 688, 673, 660, 534, 519, 488, 450, 440; ^1^H-NMR (500 MHz, CDCl_3_) δ: 7.44 (s, 2H, 2 olefinic CH), 7.41 (s, 1H, triazole CH), 7.39–7.36 (m, 2H), 7.30–7.26 (m, 2H), 7.25–7.21 (m, 5H), 7.17–7.15 (m, 4H), 3.97 (s, 6H, 2 NCH_2_ + >NCH_2_), 2.36 (s, 6H, 2 CH_3_); ^13^C-NMR (125 MHz, CDCl_3_) δ: 187.4 (C=O), 145.2, 139.5, 137.0, 136.9 (olefinic C), 132.4, 132.3, 130.6, 129.7, 129.5, 128.7, 120.8, 120.5, 54.6 (2 NCH_2_), 52.2 (>NCH_2_), 21.4 (2 CH_3_); HRMS: *m*/*z* for C_30_H_28_N_4_O [M + H]^+^ Calcd.: 461.2335, Found: 461.2186.

**3,5-Bis((*E*)-4-methoxybenzylidene)-1-((1-phenyl-1*H*-1,2,3-triazol-4-yl)methyl)piperidin-4-one (7j)**

Yellow solid, m. p.: 167–169 °C, yield: 73%. IR: ν_max_/cm^−1^; 1673, 1596, 1568, 1505, 1460, 1441, 1418, 1304, 1264, 1164, 1116, 1084, 1044, 1027, 998, 919, 828, 795, 754, 688, 530; ^1^H-NMR (500 MHz, CDCl_3_) δ: 7.59 (s, 2H, 2 olefinic CH), 7.58 (s, 1H, triazole CH), 7.55 (t, *J* = 6.0 Hz, 2H), 7.50–7.46 (m, 2H), 7.37–7.26 (m, 5H,), 7.00–6.92 (m, 4H), 3.99 (s, 2H, >NCH_2_), 3.97 (s, 4H, 2 NCH_2_), 3.82 (s, 6H, 2 OCH_3_); ^13^C-NMR (125 MHz, CDCl_3_) δ: 187.3 (C=O), 160.4, 145.4, 137.0, 136.4 (olefinic C), 132.4, 131.1, 129.7, 128.7, 127.9, 120.9, 120.4, 114.2, 55.4 (2 OCH_3_), 54.7 (2 NCH_2_), 52.3 (>NCH_2_); HRMS: *m*/*z* for C_30_H_28_N_4_O_3_ [M + H]^+^ Calcd.: 493.2234, Found: 493.2219.

**3,5-Bis((*E*)-4-chlorobenzylidene)-1-((1-(4-fluorophenyl)-1*H*-1,2,3-triazol-4-yl)methyl)piperidin-4-one (7k)**

Yellow solid, m. p.: 185–187 °C, yield: 79%. IR: ν_max_/cm^−1^; 1675, 1611, 1581, 1515, 1485, 1407, 1321, 1263, 1233, 1177, 1092, 1045, 996, 926, 834, 817, 788, 771, 652, 527; ^1^H-NMR (500 MHz, CDCl_3_) δ: 7.75 (s, 2H, 2 olefinic CH), 7.70 (s, 1H, triazole CH), 7.57–7.54 (m, 2H), 7.38 (d, *J* = 8.5 Hz, 4H), 7.31 (d, *J* = 8.5 Hz, 4H), 7.18 (t, *J* = 8.5 Hz, 2H), 3.95 (s, 2H, >NCH_2_), 3.92 (s, 4H, 2 NCH_2_); ^13^C-NMR (125 MHz, CDCl_3_) δ: 186.9 (C=O), 162.5 (d, *J_C–F_* = 247.5 Hz, Ar–C–F, triazole fluorophenyl), 145.1, 135.6 (olefinic C), 133.3, 131.6, 129.0, 122.5, 121.0, 116.7 (d, *J_C–F_* = 23.8 Hz, Ar-CH), 54.5 (2 NCH_2_), 52.2 (>NCH_2_); HRMS: *m*/*z* for C_28_H_21_Cl_2_FN_4_O [M + H]^+^ Calcd.: 519.1149, Found: 519.1105.

**3,5-Bis((*E*)-4-fluorobenzylidene)-1-((1-(4-fluorophenyl)-1*H*-1,2,3-triazol-4-yl)methyl)piperidin-4-one (7l)**

Yellow solid, m. p.: 169–171 °C, yield: 82%. IR: ν_max_/cm^−1^; 1612, 1597, 1572, 1517, 1506, 1445, 1415, 1331, 1295, 1270, 1226, 1185, 1157, 1100, 1048, 1004, 991, 852, 799, 609, 536, 529, 498; ^1^H-NMR (500 MHz, CDCl_3_) δ: 7.81 (s, 2H, 2 olefinic CH), 7.76 (s, 1H, triazole CH), 7.62–7.59 (m, 2H), 7.50–7.43 (m, 4H), 7.24–7.20 (m, 2H), 7.16–7.12 (m, 4H), 4.00 (s, 2H, >NCH_2_), 3.97 (s, 4H, 2 NCH_2_); ^13^C-NMR (125 MHz, CDCl_3_) δ: 187.0 (C=O), 164.0 (d, *J_C–F_* = 249.0 Hz, Ar–C–F, triazole fluorophenyl), 162.9 (d, *J_C–F_* = 248.2 Hz, 2 × Ar–C–F, benzylidene fluorophenyl rings), 145.2, 135.7 (olefinic C), 135.1, 133.3, 132.5, 132.4, 131.2, 122.4, 121.0, 116.7 (d, *J_C–F_* = 21.6 Hz, Ar-CH), 115.9 (d, *J_C–F_* = 21.7 Hz, Ar-CH), 54.5 (2 NCH_2_), 52.3 (>NCH_2_); ^19^F-NMR (470.6 MHz, CDCl_3_) δ: −95.5, −112.0; HRMS: *m*/*z* for C_28_H_21_F_3_N_4_O [M + H]^+^ Calcd.: 487.1740, Found: 487.1680.

**1-((1-(4-Fluorophenyl)-1*H*-1,2,3-triazol-4-yl)methyl)-3,5-bis((*E*)-4-methylbenzylidene)piperidin-4-one (7m)**

Yellow solid, m. p.: 169–171 °C, yield: 83%. IR: ν_max_/cm^−1^; 1673, 1601, 1580, 1513, 1443, 1411, 1324, 1293, 1262, 1228, 1177, 1158, 1099, 1076, 1044, 988, 927, 839, 796, 752, 703, 654, 608, 528, 489; ^1^H-NMR (500 MHz, CDCl_3_) δ: 7.80 (s, 2H, 2 olefinic CH), 7.65 (s, 1H, triazole CH), 7.53–7.51 (m, 2H), 7.29 (d, *J* = 7.5 Hz, 4H), 7.21 (d, *J* = 8.0 Hz, 4H), 7.15 (t, *J* = 8.5 Hz, 2H), 3.96 (s, 4H, 2 NCH_2_), 3.95 (s, 2H, >NCH_2_), 2.36 (s, 2 CH_3_); ^13^C-NMR (125 MHz, CDCl_3_) δ: 187.4 (C=O), 162.4 (d, *J_C–F_* = 248.1 Hz, Ar–C–F, triazole fluorophenyl), 139.5, 136.8 (olefinic C), 132.3, 132.2, 130.6, 129.4, 122.5, 122.4, 121.0, 116.5 (d, *J_C–F_* = 21.5 Hz, Ar-CH), 54.7 (2 NCH_2_), 52.2 (>NCH_2_), 21.4 (2 CH_3_); HRMS: *m*/*z* for C_30_H_27_FN_4_O [M + H]^+^ Calcd.: 479.2241, Found: 479.2533.

**1-((1-(4-Fluorophenyl)-1*H*-1,2,3-triazol-4-yl)methyl)-3,5-bis((*E*)-4-methoxybenzylidene)piperidin-4-one (7n)**

Yellow solid, m. p.: 173–175 °C, yield: 71%. IR: ν_max_/cm^−1^; 1674, 1598, 1578, 1507, 1461, 1440, 1419, 1324, 1304, 1235, 1194, 1167, 1117, 1084, 1044, 1028, 998, 929, 921, 828, 793, 783, 608, 530; ^1^H-NMR (500 MHz, CDCl_3_) δ: 7.81 (s, 2H, 2 olefinic CH), 7.68 (s, 1H, triazole CH), 7.36 (d, *J* = 7.5 Hz, 2H), 7.35–7.21 (m, 4H), 7.17 (d, *J* = 8 Hz, 2H), 6.93 (d, *J* = 7.5 Hz, 4H), 3.99 (s, 6H, 2 OCH_3_), 3.83 (s, 6H, 2 NCH_2_ + >NCH_2_); ^13^C-NMR (125 MHz, CDCl_3_) δ: 182.0 (C=O), 160.3 (d, *J_C–F_* = 248.1 Hz, Ar–C–F, triazole fluorophenyl), 143.1, 138.3, 136.8 (olefinic C), 132.4, 130.5, 128.1, 122.5, 122.4, 116.5 (d, *J_C–F_* = 21.5 Hz, Ar-CH), 55.4 (2 OCH_3_ + 2 NCH_2_), 52.3 (>NCH_2_); HRMS: *m*/*z* for C_30_H_27_FN_4_O_3_ [M + H]^+^ Calcd.: 511.2139, Found: 511.2111.

### 4.2. Antibacterial Bio-Assay

The synthesized compounds (**7a**–**n**), in addition to CIP (reference standard drug), were screened for their antibacterial properties against the Gram-positive bacteria *S. aureus* (ATCC 25923) and *E. faecalis* (ATCC 29212) and the Gram-negative bacterium *E. coli* (ATCC 25922) by the agar dilution standard method [37,38]. The tested compounds were dissolved in dimethylsulfoxide (DMSO). They were further diluted in Mueller-Hinton broth (MHB) to obtain a final concentration of 1250 μg/mL. An inoculum of approximately 1.5 × 10^8^ colony-forming units (CFU) per spot was applied to the surfaces of Mueller-Hinton agar plates containing graded concentrations of the respective compounds. The plates were incubated at 37 °C for 18 h. The spot with the lowest compound concentration showing growth was defined as the MIC (minimum inhibitory concentration). All the organisms used in this study were standard strains obtained from the American Type Culture Collection (ATCC). All MIC experiments were performed in duplicate (2 wells per compound). A negative control (DMSO) was included in each experiment. Table 2 presents the experimentally determined MIC values for all tested compounds.

### 4.3. DNA Gyrase Inhibition

The synthesized compounds discovered with promising anti-*S. aureus* properties **7a**, **7e**, **7f**, **7l**, and **7n** were subjected for DNA gyrase supercoiling assessment (standard agarose gel electrophoresis methodology followed by densitometry analysis using ImageJ software Image J 1.54) relative to CIP (standard reference drug) [41]. In this assay, inhibition of DNA gyrase prevents the conversion of relaxed DNA to supercoiled DNA. The resulting DNA fragments were visualized by agarose gel electrophoresis and quantified using ImageJ**.** The percentage inhibition was calculated using Equation (1).

(1) $$\%\;inhibition=\frac{Relaxed\;DNA}{\left(Relaxed\;DNA+Supercoiled\;DNA\right)}\times 100$$

Different concentrations (10, 5, 2.5, 1.25, and 0.625 μM) were considered for testing the compounds. The IC_50_ values were calculated using GraphPad Prism 5 (nonlinear regression, normalized response–variable slope), and the SEM was calculated using SPSS 16.

### 4.4. Antibiofilm Properties

Quantitative estimation of anti-biofilm properties of the promising agents observed **7a**, **7e**, **7f**, **7l**, and **7n**, and the standard reference CIP, utilizing MDR *S. aureus* (ATCC33591), was assayed by the standard technique with the microplate assay using 96-well polystyrene microtiter plates [50].

The tested isolates were grown in tryptic soy broth (TSB) at 37 °C for 24 h. The bacterial culture was adjusted to the turbidity of a 0.5 McFarland standard, then diluted 1:50 in freshly prepared TSB supplemented with 0.25% glucose. For the negative control, 200 μL of TSB without bacteria was used. Aliquots of the prepared bacterial suspension (200 μL) were added to wells of sterile polystyrene microtiter plates and incubated at 37 °C for 24 h. Next, the bacterial culture was removed, and the wells were washed three times with 250 μL phosphate-buffered saline (PBS) to remove planktonic bacteria. The biofilm was then fixed with 200 μL methanol per well for 15 minutes The attached biofilms were stained with 200 μL crystal violet (0.1%) for 10 min. at room temperature, the excess dye was discarded, and the wells were washed with distilled water and air dried. Finally, the dye bound to the adherent cells was dissolved in 200 μL of 95% ethanol.

The optical density (OD) at 570 nm (OD_570_), an index of bacterial biofilm formation, was measured using an ELISA microtiter plate reader. Each assay was performed in triplicate, and the mean OD and standard error of the mean (SEM) for each isolate were calculated.

### 4.5. Anti-Efflux Properties

The promising agents observed **7a**, **7e**, **7f**, **7l**, and **7n**, and the standard reference CIP utilizing MDR *S. aureus* (ATCC33591) was assayed by the standard technique for anti-efflux pumping properties [54]. Strains were cultured overnight at 37 °C and used to inoculate fresh medium, which was incubated for a further 5 h at 37 °C until they reached mid-log phase (OD_600_ = 0.6). Bacterial cells were collected by centrifugation at 4000× *g* for 4 min and resuspended in 1 M PBS. The optical density of all suspensions was adjusted to 0.3 at λ = 600 nm, and 176 µL of cell suspension with 4 µL of efflux inhibitors (CCCP) was transferred to wells of a 96-flat-bottomed, black-well plate. The heat-inactivated control isolate (10 min, 90 °C) was used as a positive control. The plate was transferred to a Varioskan LUX Multimode Microplate Reader, incubated at 37 °C, and 20 µL of EtBr (25 µM) was added to each well, giving a final concentration of 2.5 µM. Fluorescence was read from the top of the wells for over 45 min using excitation and emission filters of λ = 515 and 600 nm, respectively.

Differences in accumulation in the presence of efflux pump inhibitors compared with the absence of efflux pump inhibitors were analyzed. The steady-state accumulation level was defined as the level at which maximum fluorescence was achieved and remained constant over the time period. The heat-killed bacterial isolate with the highest fluorescence was used as the positive control.

### 4.6. Antiproliferation Properties

The cell lines used in the current study were kindly provided by Prof. Stig Linder, Karolinska Institute, Stockholm, Sweden, and were originally purchased from ATCC. The synthesized compounds **7a**–**n** were screened for their antiproliferation properties against HCT116 (colon), and MCF7 (breast) cancer cell lines by the standard mitochondrial-dependent reduction in yellow MTT [3-(4,5-dimethylthiazol-2-yl)-2,5-diphenyl-tetrazolium bromide] to purple formazan technique [55]. 5-Fluorouracil and sunitinib were used as standard reference drugs. Cells were suspended in DMEM medium for MCF7 and McCoy’s 5A for HCT116 in addition to 1% antibiotic–antimycotic mixture (10,000 μg mL^−1^ potassium penicillin, 10,000 μg mL^−1^ streptomycin sulfate, and 25 μg mL^−1^ amphotericin B), 10% fetal bovine serum and 1% L-glutamine at 37 °C, under 5% CO_2_ and 95% humidity. Cells were seeded at a concentration of 30,000 cells per well in fresh complete growth medium in 96-well tissue culture microtiter plates for 24 h. Media was aspirated, fresh complete medium was added and cells were incubated with different concentrations of the tested compound to give a final concentration of 10, 5, 2.5, and 1.25 μM. 0.5% DMSO was used as a negative control. Triplicate wells were prepared for each individual dose. After 72 h of incubation, the medium was aspirated, 40 μL of MTT salt (2.5 mg mL^−1^) was added to each well, and the plate was incubated for a further 4 h at 37 °C. To stop the reaction and dissolve the crystals formed, 150 μL of 10% sodium dodecyl sulfate (SDS) in deionized water was added to each well, and the wells were incubated overnight at 37 °C. The absorbance was then measured at 570 nm and a reference wavelength of 595 nm.

Data were recorded as mean values from experiments performed in triplicate for each individual dose, measured by the MTT assay. Control experiments showed no significant change compared to the DMSO vehicle. The cell survival fraction was calculated using the following equation.

(2) $$\mathrm{Surviving}\;\mathrm{fraction}=\frac{Opticaldensity\left(O.D.\right)\;of\;treated\;cells}{O.D.\;of\;control\;cells}$$

The synthesized agents were also tested against RPE1 (a normal human immortalized retinal pigment epithelial cell line) in DMEM-F12 medium to determine their toxicity/selectivity relative to the cancer cell lines used.

The IC_50_ (concentration required to inhibit cell growth by 50% relative to the control) was determined using GraphPad Prism version 5. Statistical calculations for the determination of the standard error mean (SEM) values were performed using SPSS 16 software.

### 4.7. QSAR Studies

The geometry of the tested compounds was optimized using the molecular mechanics force field (MM+) and the semi-empirical AM1 method implemented in HyperChem 8.0 (http://www.hyper.com, 18 April 2026). The structures were fully optimized without constraining any parameters, thus bringing all geometric variables to their equilibrium values. The energy minimization protocol employed the Polak–Ribiere conjugate gradient algorithm [59]. Convergence to a local minimum was achieved when the energy gradient was ≤ 0.01 kcal mol^−1^. The RHF (Restricted Hartree–Fock) method was used for spin pairing in the semi-empirical tool.

2D-QSAR studies were undertaken using the comprehensive descriptors for structural and statistical analyses (CODESSA-Pro) software [61]. The optimized structures of the tested compounds were uploaded to CODESSA-Pro, which includes MOPAC capability for the final geometry optimization. CODESSA-Pro calculated 673 molecular descriptors, including constitutional, topological, geometrical, charge-related, semi-empirical, molecular-type, atomic-type, and bond-type descriptors for the exported compounds. Different mathematical transformations [including property (MIC), 1/property, log(property), and 1/log(property)] of the experimentally observed activity of the set of compounds were used to search for the best QSAR model. The best multi-linear regression (BMLR) technique was utilized which is a stepwise search for the best *n* parameter regression equations (where *n* stands for the number of descriptors used), based on the highest *R*^2^ (squared correlation coefficient), *R*^2^cvOO (squared cross-validation “leave-one-out, LOO” coefficient), *R*^2^cvMO (squared cross-validation “leave-many-out, LMO” coefficient), *F* (Fisher statistical significance criteria) values, and *s* (standard deviation). The QSAR models were generated (obeying the thumb rule that establishes an appropriate ratio between the data points and the number of QSAR descriptors) (Supplementary Tables S1–S3, Figure 6).

The descriptors mentioned in the QSAR model can be calculated by the following equations [62].

(3) $$E_{ne}\left(AB\right)=\sum_{B}\sum_{\mu ,\nu \in A}P_{\mu \nu }\left\langle \mu |\frac{Z_{B}}{R_{iB}}|\nu \right\rangle$$

Since, *A* and *B* are two different atoms, $P_{\mu \nu }$ is the density matrix elements over the atomic basis $\left\{\mu \nu \right\}$, $Z_{B}$ is the charge of the atomic nucleus *B*, $R_{iB}$ is the distance between the electron and the atomic nucleus *B*, and $\left\langle \mu |\frac{Z_{B}}{R_{iB}}|\nu \right\rangle$ is the electron–nuclear attraction integrals on an atomic basis $\left\{\mu \nu \right\}$.

(4) ICk=−∑i=1knin log2 nin

Since, *n_i_* is the number of atoms in the *i*th class, *n* is the total number of atoms in the molecule, and *k* is the number of atomic layers in the coordination sphere around a given atom that are accounted for.

### 4.8. Molecular Docking Studies

This analysis used the protein structure retrieved from the Protein Data Bank (PDB ID: 3TTZ) [64]. Molecular docking study was carried out using the CDOCKER protocol in Discovery Studio 4.1 software suite (Accelrys Inc.), employing the standard CDOCKER technique (RMS gradient = 0.091; re-docking of the co-crystallized ligand allowed docking validation, active site sphere, radius = 9.208 Å; X, Y, Z = 0.220, 3.716, 24.113 Å). The X-ray crystallographic structure with PDB ID 3TTZ was downloaded, and the unneeded water molecules were removed. Protein structure was checked for any incomplete residues, followed by the addition of hydrogen atoms at the appropriate position(s) of amino acids. Optimized by the standard protocol (force field: CHARMm, and partial charge: MMFF94) with identification of the active/binding site (a sphere) to be utilized in the following docking steps. The chemical structure of the tested compounds should also be optimized (force field: CHARMm, Partial charge: MMFF94) then, the binding mode of interaction of the synthesized compounds (**7a, 7e, 7f, 7l,** and **7n**) in the protein ATPase site was determined/studied (CDOCKER protocol) for determining the bonding/non-bonding interactions with the active site/pocket protein amino acid(s).

### 4.9. Molecular Dynamics Simulations

Molecular dynamic simulations using Discovery Studio 4.1 Software were carried out for the protein backbone of PDB ID: 3TTZ, and the best pose was detected in molecular docking studies for compounds **7a** and **7l**, utilizing the standard protocol (standard dynamic cascade). For the tested agents, the poses that revealed the best docking interactions were dragged and dropped onto the protein of PDB ID: 3TTZ, optimized using the standard protocol (force field: CHARMm, partial charge: MMFF94), and then the standard MD protocol was applied [55].

Minimization 1

Algorithm: Steepest Descent, Maximum Steps: 2000, RMS Gradient: 1.0.

Minimization 2

Algorithm: Conjugate Gradient, Max. Steps: 2000, RMS Gradient: 0.1.

Heating

Simulation Time (ps): 4, Time Steps (fs): 2, Initial Temperature: 50, Target Temperature: 300, Adjust Velocity Frequency: 1000, Save Results Interval (ps): 2.

Equilibration

Simulation Time (ps): 20, Time Step (fs): 2, Target Temperature: 300, Adjust Velocity Frequency: 1000, Save Results Intervals (ps): 2.

Production

Simulation Time (ps): 200, Time Steps (fs): 2, Target Temperature: 300, Type: NVT.

Implicit Solvent Model

Generalized Born with a simple Switching (GBSW). RMSD and RMSF were obtained upon applying trajectory analysis on the output files obtained from the molecular dynamics simulations.

## 5. Conclusions

In summary, this study describes the design, synthesis, and multidisciplinary evaluation of a series of curcumin-inspired 3,5-diarylidene-4-piperidone derivatives functionalized through 1,2,3-triazole conjugation. The modular click-chemistry-based synthetic strategy enabled efficient preparation and diversification of the target scaffold in yields ranging from good to excellent.

Biological evaluation demonstrated that several derivatives exhibit selective antibacterial activity against *S. aureus*, including multidrug-resistant strains, while showing limited or no activity against the Gram-negative bacterium *E. coli*. Among the evaluated compounds, **7l** emerged as a particularly promising lead by combining potent antibacterial activity with strong inhibition of *S. aureus* DNA gyrase. Additional compounds displayed measurable antibiofilm and anti-efflux activity, supporting a multifunctional antibacterial profile across the series.

Evaluation of antiproliferative properties identified distinct structure-dependent responses, while the most promising antibacterial derivatives exhibited minimal cytotoxicity toward normal RPE1 cells, supporting favorable preliminary selectivity. Collectively, these findings indicate that antibacterial and antiproliferative activities can be modulated independently within this scaffold.

Computational analyses, including QSAR modeling, molecular docking, MD simulations, and physicochemical assessment, complemented the experimental observations and provided additional insight into structure–activity relationships, binding behavior, and developability characteristics. Computational findings support DNA gyrase B as an important molecular target for selected compounds and suggest that antibacterial activity may arise from contributions of multiple mechanisms.

Overall, this work identifies triazole-conjugated 3,5-diarylidene-4-piperidones as promising multifunctional scaffolds for the development of new antibacterial agents and lays the foundation for future optimization guided by integrated biological and computational approaches. Future studies will focus on expanded mechanistic validation, pharmacological characterization, and in vivo evaluation of the most promising lead compounds.

## Figures and Tables

**Table 1:** Optimization of reaction conditions for the CuAAC (Click reaction) in the synthesis of compounds **7a**–**n**.

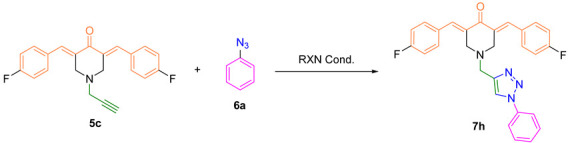						
**Entry**	**Solvent**	**Catalyst/Reagent System**	**Heating Method**	**Temperature (°C)**	**Time (h)**	**Yield (%)**
1	DCM/H_2_O (1:1)	CuSO_4_·5H_2_O/Sodium ascorbate	Conventional stirring	rt	24	0
2	*^t^*BuOH/H_2_O (1:1)	CuSO_4_·5H_2_O/Sodium ascorbate	Conventional heating	60	18	10
3	MeOH	CuSO_4_·5H_2_O/Sodium ascorbate	Reflux	65	12	0
4	CH_3_CN	CuI/DIPEA	Conventional heating	70	10	0
5	DMF	CuSO_4_·5H_2_O/Sodium D-isoascorbate	Conventional heating	70	8	31
6	DMF	CuSO_4_·5H_2_O/Sodium D-isoascorbate	Microwave irradiation	70	5	58
7	DMF	CuSO_4_·5H_2_O Sodium D-isoascorbate	Microwave irradiation	70	4	89

**Table 2:** Antimicrobial properties (MIC ^a^) of the tested compounds.

Compd.	*Staphylococcus aureus*		*Enterococcus faecalis*	
	μg/mL ± SEM ^b^	μM	μg/mL ± SEM ^b^	μM
**7a**	8 ± 0.23	17.9	256 ± 7.54	573.3
**7b**	256 ± 5.16	496.7	1024 ± 9.89	1986.7
**7c**	256 ± 3.27	530.5	1024 ± 6.24	2122.1
**7d**	128 ± 6.22	269.7	512 ± 9.21	1078.8
**7e**	16 ± 0.15	31.6	256 ± 8.44	505.3
**7f**	8 ± 0.04	12.8	256 ± 5.63	408.5
**7g**	64 ± 0.68	127.6	256 ± 3.76	510.6
**7h**	128 ± 2.33	273.2	256 ± 7.44	546.4
**7i**	64 ± 1.54	139.0	256 ± 3.99	555.8
**7j**	128 ± 6.21	259.9	512 ± 6.22	1039.4
**7k**	512 ± 2.99	985.8	1024 ± 8.47	1971.5
**7l**	4 ± 0.42	8.2	256 ± 5.28	526.2
**7m**	128 ± 1.73	267.5	512 ± 4.26	1069.9
**7n**	4 ± 0.74	7.8	128 ± 5.21	250.7
**CIP** ^c^	1024 ± 4.35	3090.4	512 ± 3.81	1545.2

^a^ MIC, minimum inhibitory concentration; ^b^ SEM, standard error mean; ^c^ CIP, ciprofloxacin.

**Table 3:** Inhibitory properties (IC_50_, μM) of the tested compounds against *S. aureus* DNA gyrase supercoiling.

Compd.	IC_50_ (μM) ± SEM
CIP	2.93 ± 0.04
**7a**	4.56 ± 0.21
**7e**	9.85 ± 0.15
**7f**	>10.00 ± 0.93
**7l**	3.20 ± 0.01
**7n**	>10.00 ± 0.04

**Table 4:** Biofilm properties of the tested agents against MDR *S. aureus* (ATCC 33591).

Compd.	MIC, μg/mL ± SEM	Fold Change ^a^	% Biofilm Detected
CIP	15.6 ± 0.47	31.3	23.6
**7a**	31.3 ± 0.23	62.5	72.5
**7e**	31.3 ± 0.10	62.5	20.2
**7f**	125 ± 1.92	250	62.7
**7l**	31.3 ± 0.99	62.5	39.1
**7n**	62.5 ± 1.00	125	54.4

^a^ Fold change, double MIC value.

**Table 5:** Anti-efflux pumping properties (% activity values) of the tested compounds against MDR *S. aureus* (ATCC 33591).

Compd.	MIC, μg/mL ± SEM	½ MIC, μg/mL	% Efflux Pumping Inhibitory Activity
CCCP	10.0 ± 0.27	5.0	98.9
**7a**	31.3 ± 0.23	15.6	90.7
**7e**	31.3 ± 0.10	15.6	52.2
**7f**	125 ± 1.92	62.5	39.1
**7l**	31.3 ± 0.99	15.6	42.8
**7n**	62.5 ± 1.00	31.3	83.7

**Table 6:** Antiproliferation properties of the synthesized compounds **7a**–**n** and reference drugs (sunitinib and 5-fluorouracil).

Compd.	IC_50_ (μM ± SEM)		
	HCT116	MCF7	RPE1
**7a**	>10.00 ± 0.64	>10.00 ± 0.19	>10.00 ± 0.35
**7b**	9.59 ± 0.18	8.70 ± 0.20	>10.00 ± 0.56
**7c**	6.01 ± 0.04	7.73 ± 0.17	>10.00 ± 0.44
**7d**	>10.00 ± 0.02	>10.00 ± 0.30	>10.00 ± 0.65
**7e**	>10.00 ± 0.04	>10.00 ± 0.34	>10.00 ± 0.21
**7f**	>10.00 ± 0.05	>10.00 ± 0.56	>10.00 ± 0.37
**7g**	8.60 ± 0.31	>10.00 ± 0.32	>10.00 ± 0.47
**7h**	6.06 ± 0.09	>10.00 ± 0.37	>10.00 ± 0.25
**7i**	>10.00 ± 0.16	>10.00 ± 0.31	>10.00 ± 0.19
**7j**	>10.00 ± 0.71	>10.00 ± 0.16	>10.00 ± 0.66
**7k**	5.26 ± 0.19	>10.00 ± 0.58	>10.00 ± 0.58
**7l**	>10.00 ± 0.39	>10.00 ± 0.36	>10.00 ± 0.53
**7m**	>10.00 ± 0.25	>10.00 ± 0.57	>10.00 ± 0.66
**7n**	>10.00 ± 0.09	>10.00 ± 0.46	>10.00 ± 0.74
Sunitinib ^a^	9.67 ± 0.22	3.97 ± 0.32	NT
5-Fluorouracil ^a^	20.43 ± 1.99	3.15 ± 0.44	NT

^a^ Reference [55]; NT: Not Tested.

**Table 7:** Docking interactions of the tested compounds in the active site of PDB ID: 3TTZ.

Compd.	Docking Score (kcal mol^−1^)	Hydrogen Bonding	Non-Bonding Interaction
**7a**	−46.033	---	π-cation interaction: phenyl … Arg84 π-alkyl interactions: triazole … Pro87, ylidene Ph … Ile86
**7e**	−55.881	triazolyl N … Arg144	π-cation interactions: triazol … Arg84, piperidinyl N … Glu58 amide π-stacked interaction: ylidene Ph … Asn54 π-alkyl interactions: triazole … Pro87, ylidene Ph … Pro87, ylidene Ph … Ile86
**7f**	−64.416	OMe … Arg144, piperidinyl C=O … Gly85	π-cation interaction: ylidene Ph … Arg84 π-alkyl interactions: 2 ylidene Ph … Pro87, ylidene Ph … Ile86
**7l**	−56.578	F … Asn54, piperidinyl C=O … Gly85, piperidinyl N … Glu58	π-alkyl interactions: Ph … Ala61, ylidene Ph … Pro87, ylidene Ph … Ile86
**7n**	−53.653	piperidinyl C=O … Gly85	π-alkyl interactions: ylidenr Ph … Ile86, ylidene Ph … Pro87, triazole … Ile102
**Co-crystallized ligand**	−53.434	thienyl S … Arg144, carboxylic O … Arg144	amide π-stacked interaction: pyrrolidinyl … Asn54 π-alkyl & alkyl interactions: thienyl … Pro87, pyrrolidinyl … Ile86, Cl … Ile86, Cl … Ile102, Cl … Ile175, Cl … Ile51

**Table 8:** In silico pharmacokinetic properties of curcumin mimic hybrids.

Compound	MW (g/mol)	HBA	HBD	Rotatable Bonds	logP	TPSA (Å^2^)	MR	BBB
Ideal	<500.00	<10	<5	<10	2–5	<140	40–130	−
Curcumin	368.38	6	2	8	3.20	93.06	102.80	−
**7a**	446.54	4	0	5	4.95	51.02	139.45	+
**7b**	515.43	4	0	5	6.21	51.02	149.47	+
**7c**	482.52	6	0	5	5.15	51.02	139.36	+
**7d**	474.60	4	0	5	4.99	51.02	149.38	+
**7e**	506.59	6	0	7	4.39	69.48	152.43	−
**7f**	626.70	10	0	11	4.43	106.40	178.40	−
**7g**	501.41	4	0	5	5.38	51.02	144.50	+
**7h**	468.50	6	0	5	5.19	51.02	134.40	+
**7i**	460.57	4	0	5	4.69	51.02	144.41	+
**7j**	492.57	6	0	7	4.09	69.48	147.46	−
**7k**	519.40	5	0	5	5.95	51.02	144.46	−
**7l**	486.49	7	0	5	5.75	51.02	134.35	+
**7m**	478.56	5	0	5	5.24	51.02	144.37	+
**7n**	510.56	7	0	7	4.65	69.48	147.42	−

**Table 9:** Physicochemical Space of **7a**, **7e**, and **7l**. The pink region indicates the ideal physicochemical space for oral bioavailability.

7a	7e	7l
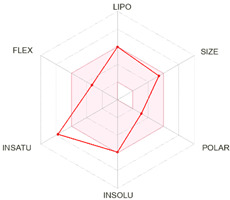	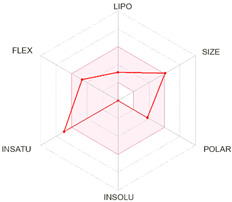	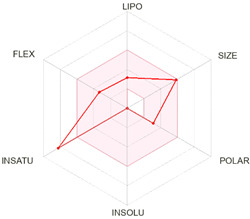

**Table 10:** Predicted toxicity data.

Compound	Probability				LD_50_ (mg/kg)
	Hepatotoxicity	Neurotoxicity	Nephrotoxicity	Cardiotoxicity	
**7a**	0.61 (−ve)	0.79 (+ve)	0.68 (−ve)	0.85 (−ve)	500
**7b**	0.58 (−ve)	0.84 (+ve)	0.66 (−ve)	0.85 (−ve)	500
**7c**	0.52 (−ve)	0.83 (+ve)	0.67 (−ve)	0.84 (−ve)	500
**7d**	0.61 (−ve)	0.78 (+ve)	0.66 (−ve)	0.87 (−ve)	500
**7e**	0.61 (−ve)	0.61 (+ve)	0.50 (+ve)	0.78 (−ve)	500
**7f**	0.53 (−ve)	0.53 (+ve)	0.50 (−ve)	0.75 (−ve)	500
**7g**	0.59 (−ve)	0.85 (+ve)	0.66 (−ve)	0.85 (−ve)	500
**7h**	0.53 (−ve)	0.84 (+ve)	0.67 (−ve)	0.84 (−ve)	500
**7i**	0.61 (−ve)	0.78 (+ve)	0.67 (−ve)	0.86 (−ve)	500
**7j**	0.61 (−ve)	0.63 (+ve)	0.50 (−ve)	0.77 (−ve)	500
**7k**	0.54 (−ve)	0.86 (+ve)	0.67 (−ve)	0.84 (−ve)	500
**7l**	0.53 (−ve)	0.84 (+ve)	0.67 (−ve)	0.84 (−ve)	500
**7m**	0.52 (−ve)	0.83 (+ve)	0.66 (−ve)	0.85 (−ve)	500
**7m**	0.50 (−ve)	0.71 (+ve)	0.51 (+ve)	0.76 (−ve)	500

## Data Availability

The original contributions presented in this study are included in the article/Supplementary Material. Further inquiries can be directed to the corresponding author.

## Supplementary Materials

The following supporting information can be downloaded at: https://www.mdpi.com/article/10.3390/ph19060935/s1. Figures S1–S76: IR, ^1^H NMR, ^13^C NMR, and HRMS spectra of compounds **5a**–**c** and **7a**–**n**; Figures S77–S79: Dose–response curves of the synthesized compounds against HCT116, MCF7, and RPE1 cell lines; Figure S80: 2D-, and 3D-docking poses of the tested compounds and co-crystallized ligand in the active site of PDB ID: 3TTZ; Tables S1–S3: QSAR descriptors, observed and predicted MIC values, and molecular descriptor values used in QSAR model development; Tables S4–S9: Molecular dynamics simulation data, including RMSD and RMSF values for the protein backbone and selected docked compounds (**7a** and **7l**) in the active site of DNA gyrase B (PDB ID: 3TTZ); Table S10: Physicochemical space analysis of curcumin-inspired hybrids (**7a**–**n**); the highlighted region represents the preferred physicochemical space associated with oral bioavailability.
