# Supplementary material for: Multifunctional Curcumin-Inspired 3,5-Diarylidene-4-Piperidones: Design, Synthesis, Biological Evaluation and Computational Mechanistic Studies
Source: Pharmaceuticals (Basel). 2026 Jun 13;19(6):935. doi: 10.3390/ph19060935 (PMC13305707; doi:10.3390/ph19060935)
Supplement: Supplementary file 1 [file pharmaceuticals-19-00935-s001.zip › pharmaceuticals-4334772-supplementary.pdf]

Supporting Information

Multifunctional Curcumin-Inspired 3,5-Diarylidene-4-Piperidones: Design, Synthesis, Biological Evaluation, and Computational Mechanistic Studies

Angel Nkosi[1], Adel S. Girgis[2], Ahmed Samir[3], Mohamed A. Morsy[4], Amira M. Shaban[5], Walid Fayad[6], Ahmed A. F. Soliman[6], Christine T. Williams[1], Shogo Mori[1], Leena Khanna[7], Guido F. Verbeck[1], Siva S. Panda [1,8,*]

[1]Department of Chemistry and Biochemistry, Augusta University, Augusta, GA,30912, USA

[2]Department of Pesticide Chemistry, National Research Centre, Dokki, Giza 12622, Egypt

[3]Microbiology Department, Faculty of Veterinary Medicine, Cairo University, Cairo, 12211 Egypt

[4]Al-Azhar Virology Research Center, Faculty of Medicine, Al-Azhar University, Cairo 71524, Egypt

[5]Botany and Microbiology Department, Faculty of Science, Beni-Suef University, Beni-Suef, 62511, Egypt

[6]Drug Bioassay-Cell Culture Laboratory, Pharmacognosy Department, National Research Centre, Dokki, Giza, 12622. Egypt

[7]University School of Basic & Applied Sciences, Guru Gobind Singh Indraprastha University, Dwarka, New Delhi-110078, India

[8]Department of Biochemistry and Molecular Biology, Augusta University, Augusta, GA,30912, USA

* Corresponding author: sipanda@augusta.edu or sspanda12@gmail.com

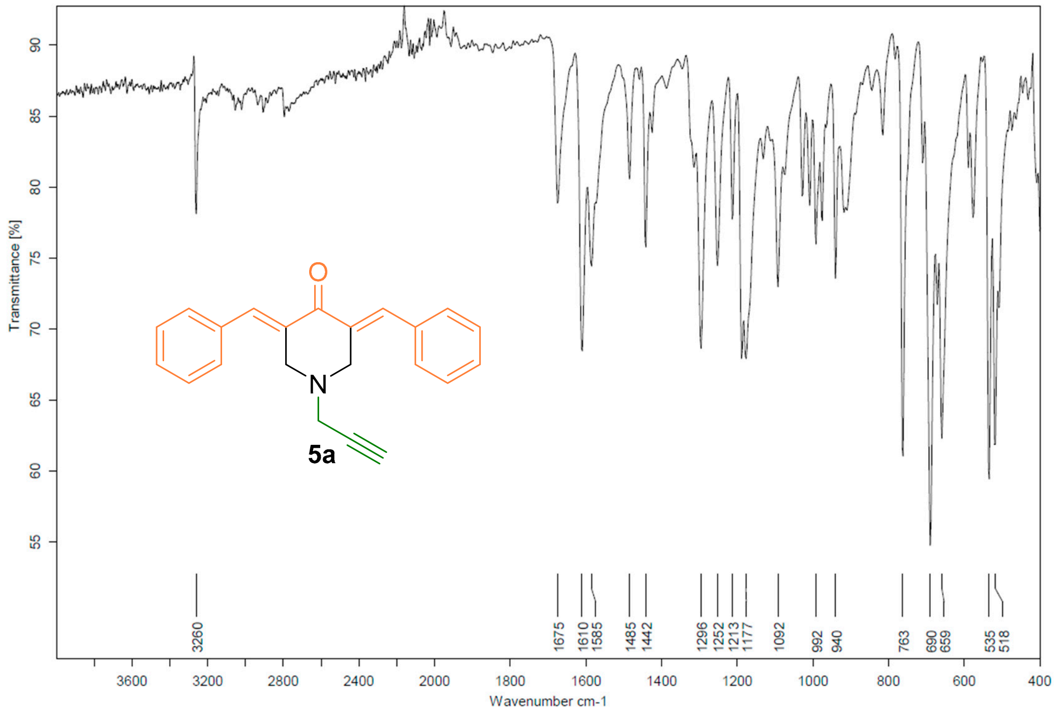

Figure S1. IR spectrum of compound **5a**.

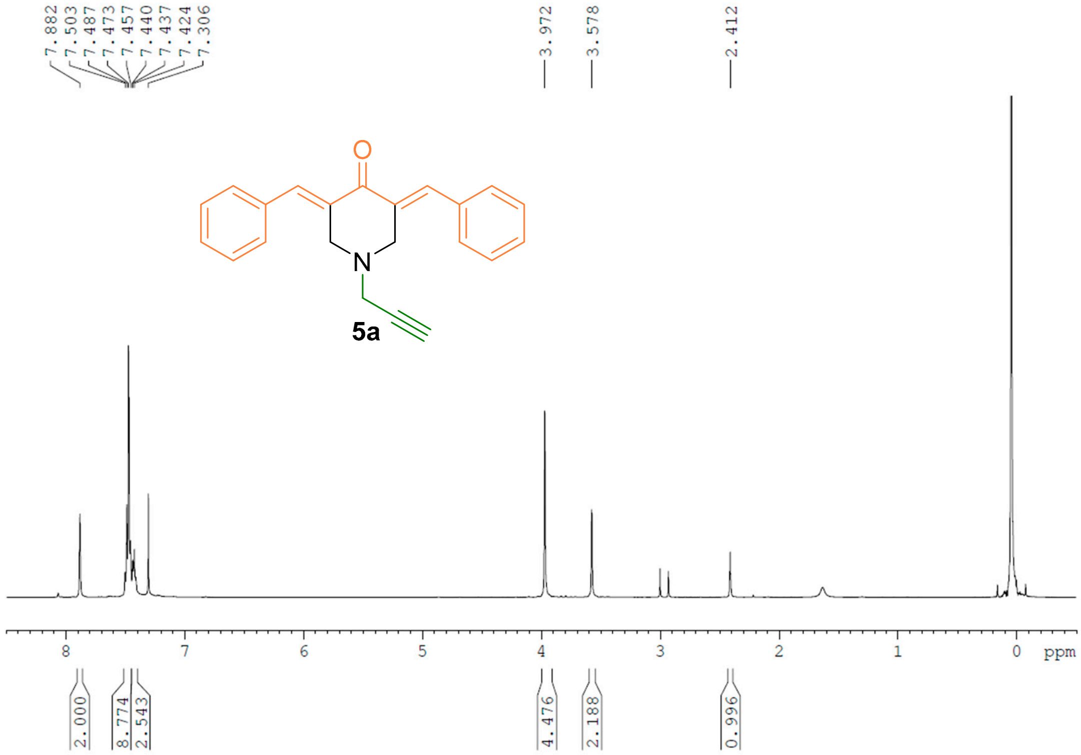

Figure S2. ^{1}H-NMR spectrum of compound **5a**.

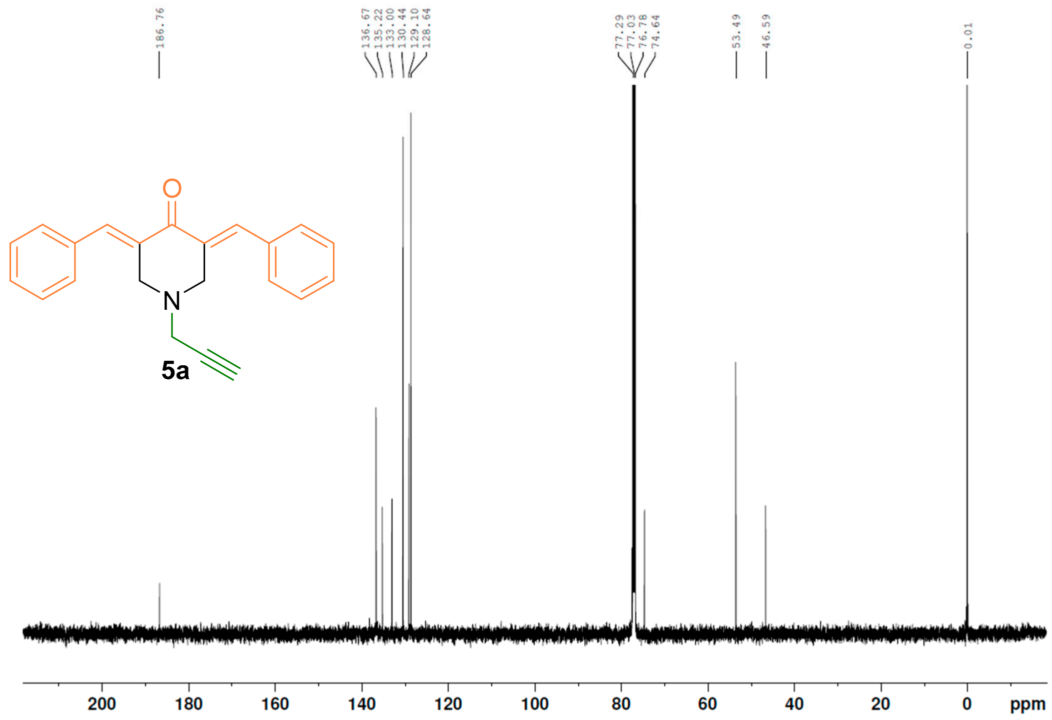

Figure S3. ^{13}C-NMR spectrum of compound **5a**.

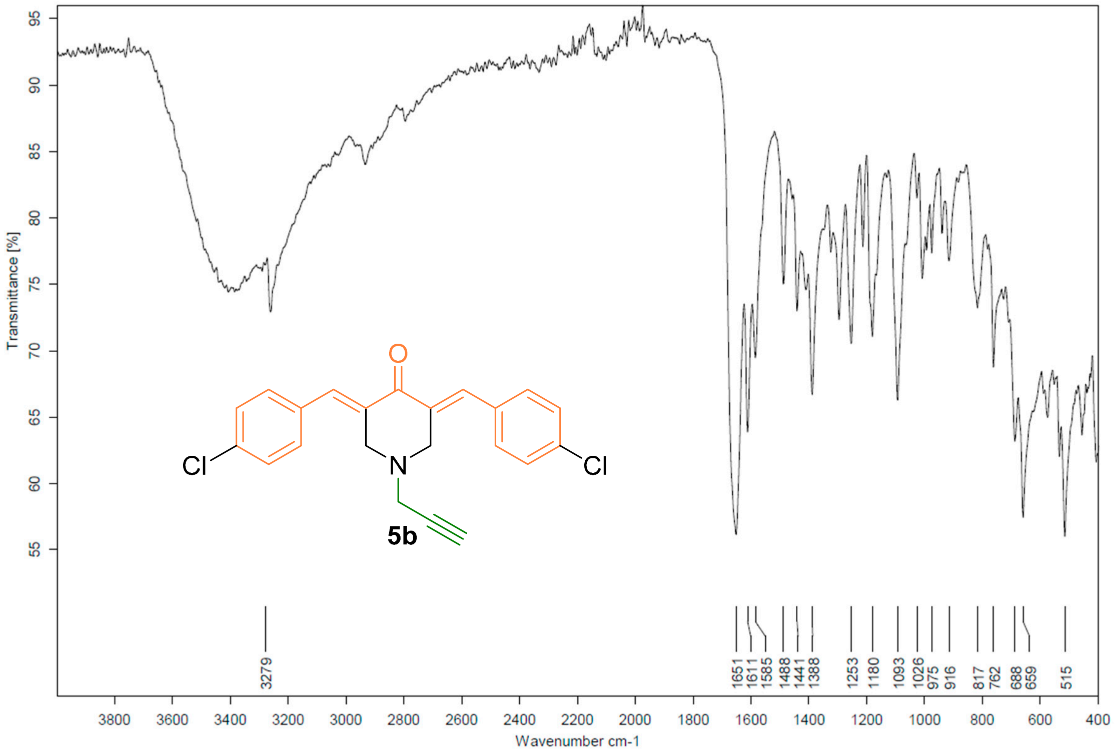

Figure S4. IR spectrum of compound **5b**.

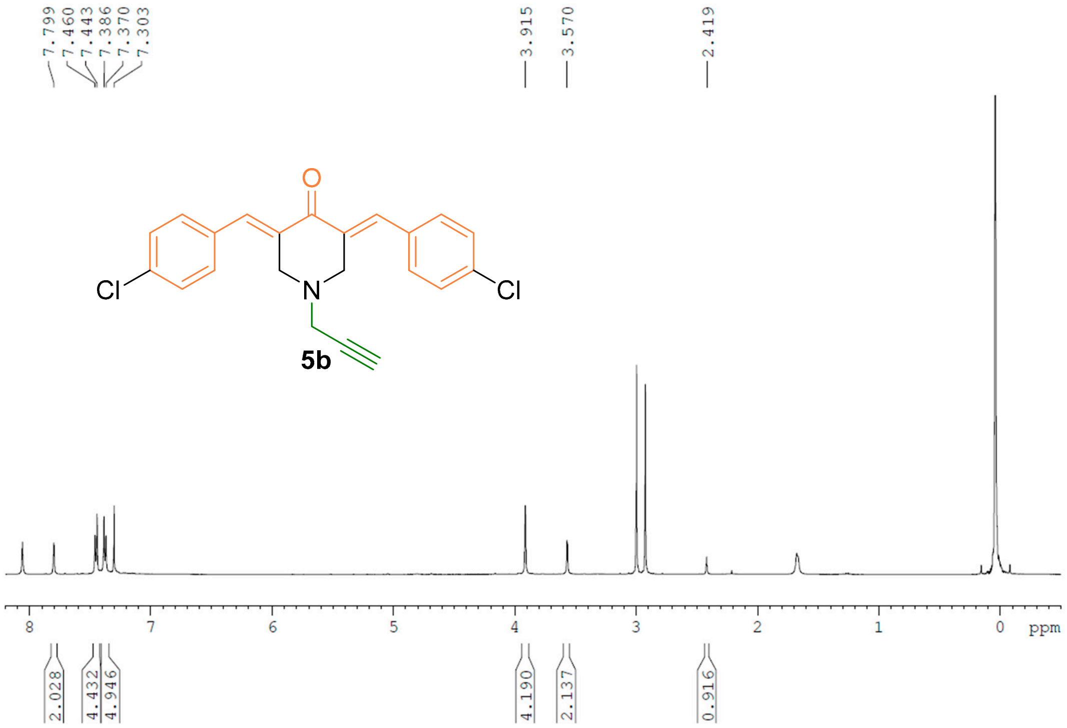

Figure S5. ¹H-NMR spectrum of compound **5b**.

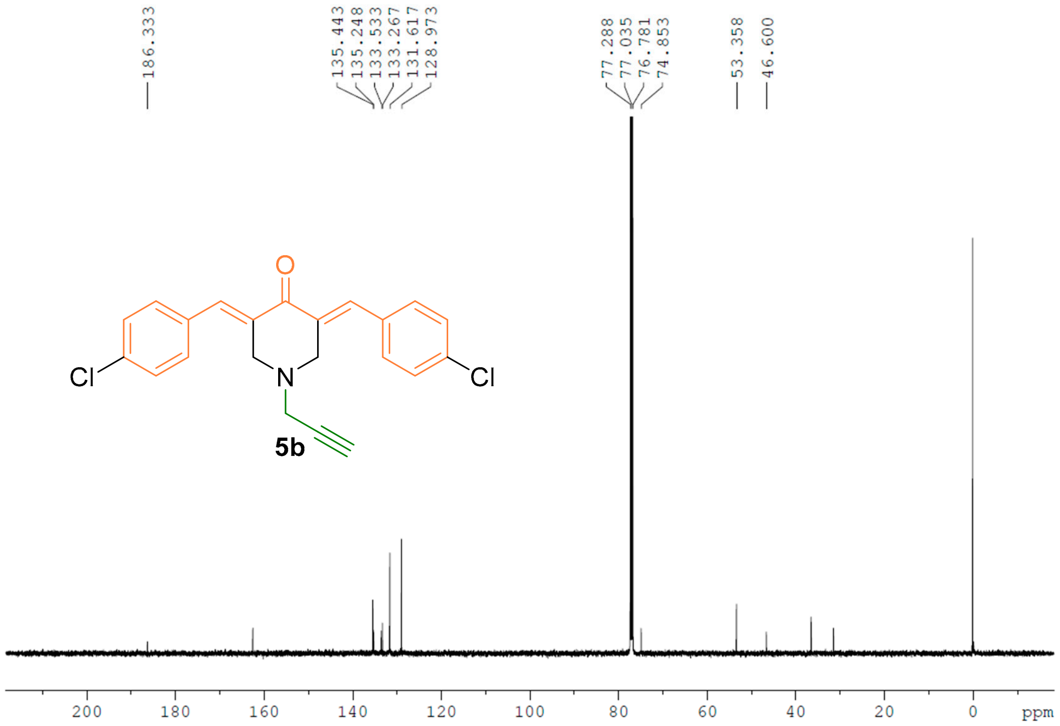

Figure S6. ¹³C-NMR spectrum of compound **5b**.

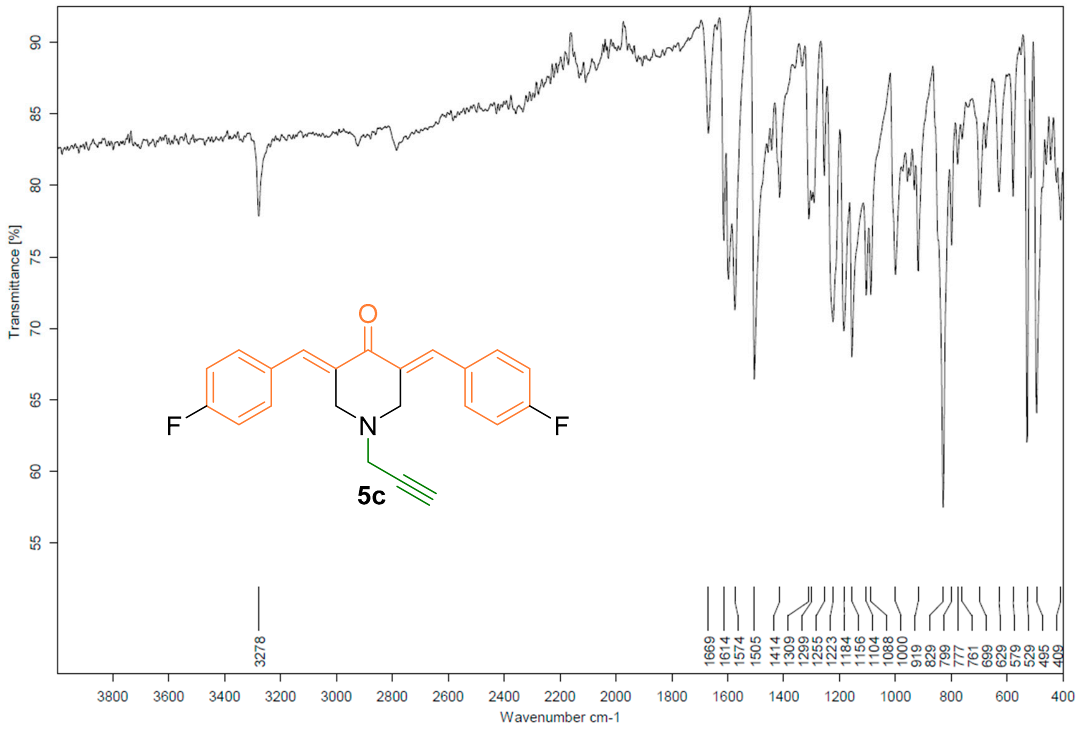

Figure S7. IR spectrum of compound **5c**.

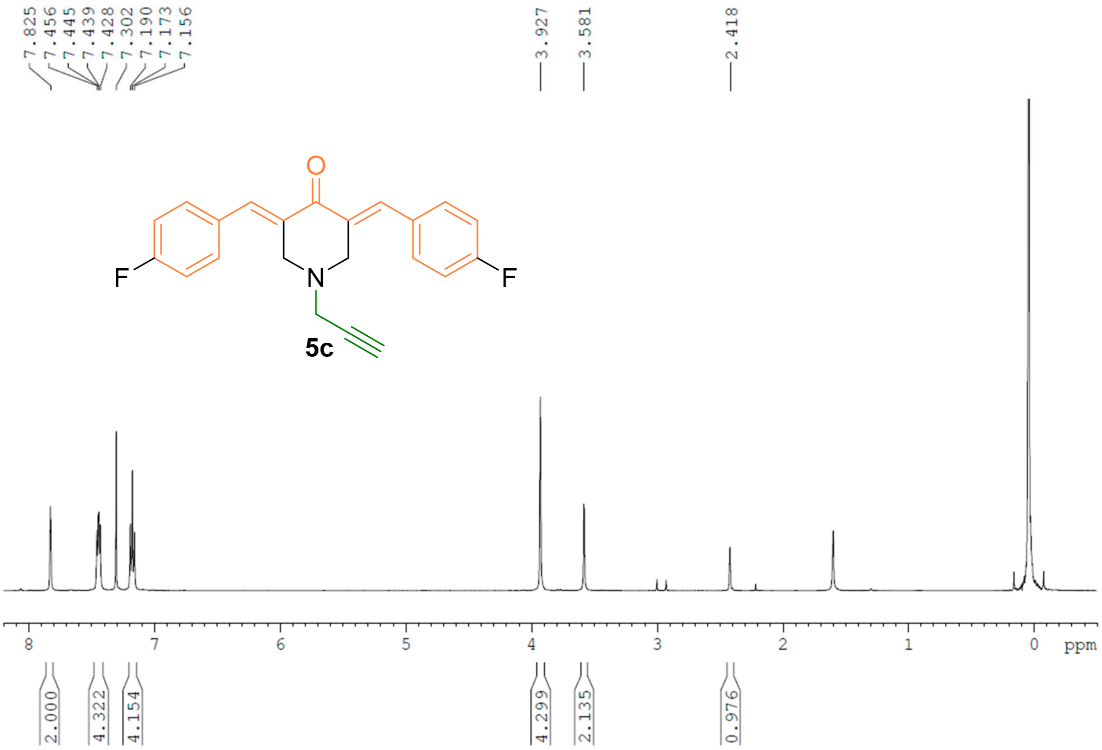

Figure S8. ^{1}H-NMR spectrum of compound **5c**.

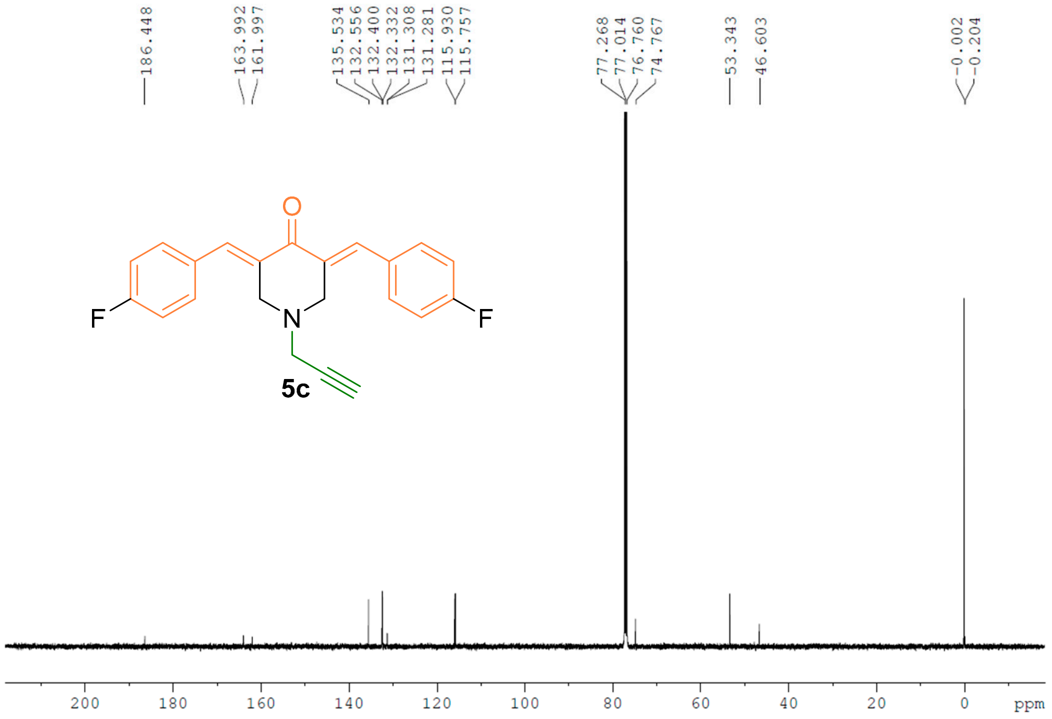

Figure S9. ^{13}C-NMR spectrum of compound **5c**.

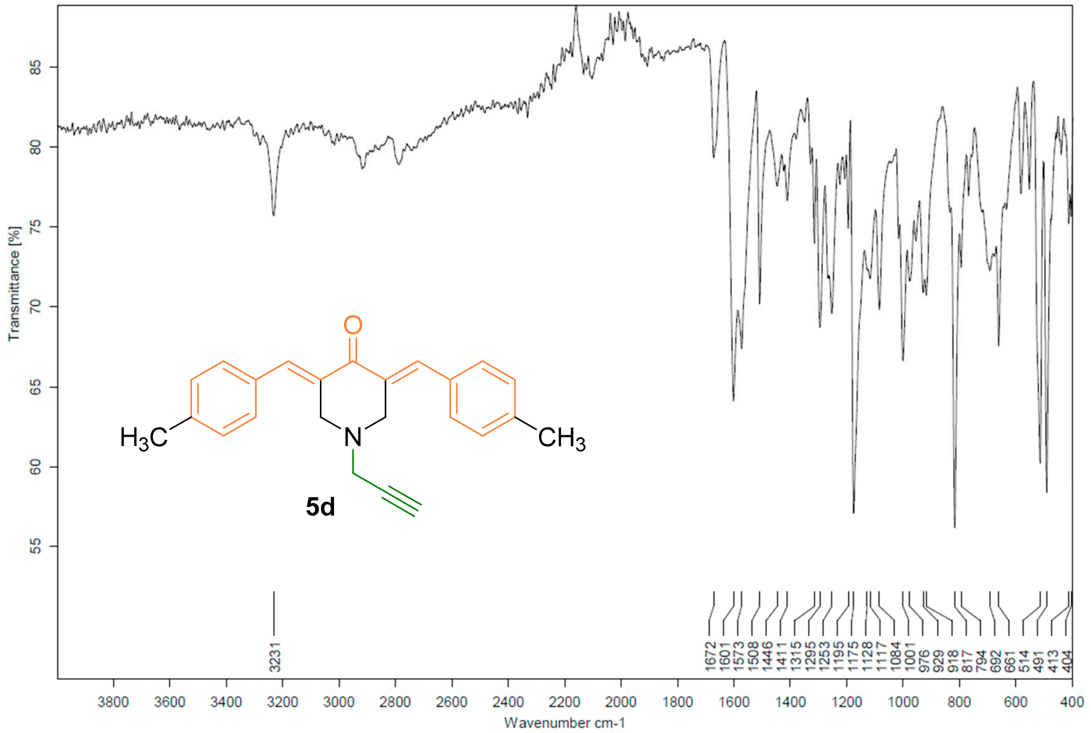

Figure S10. IR spectrum of compound **5d**.

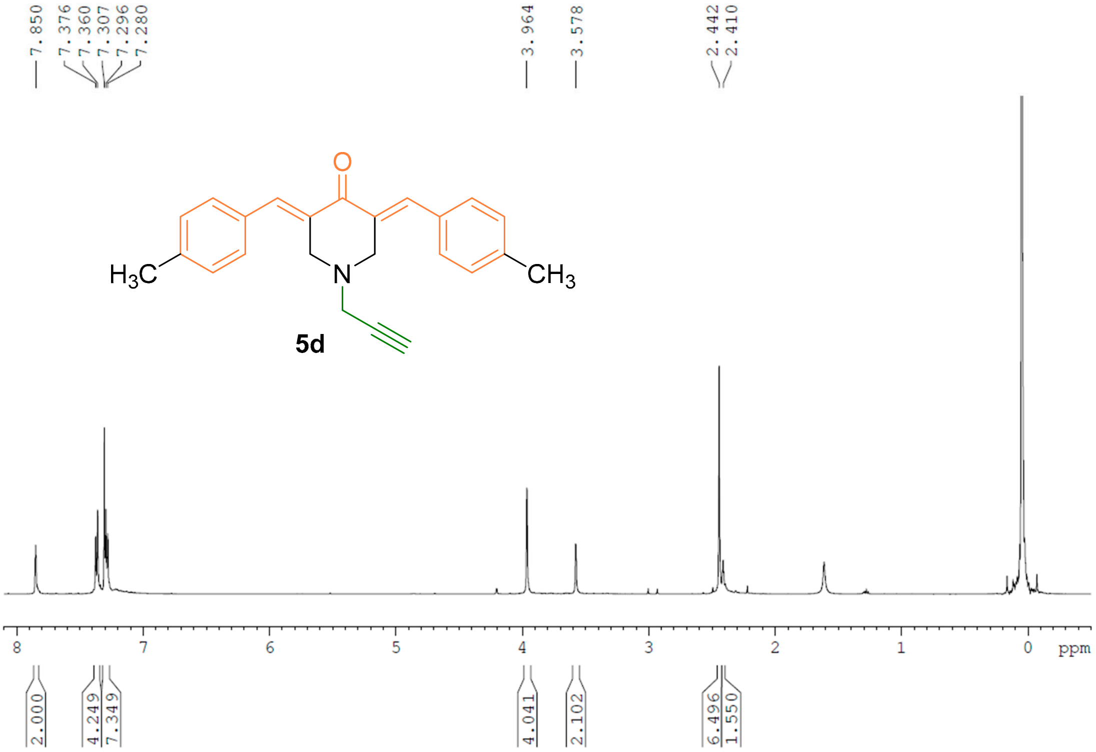

Figure S11. ^{1}H-NMR spectrum of compound **5d**.

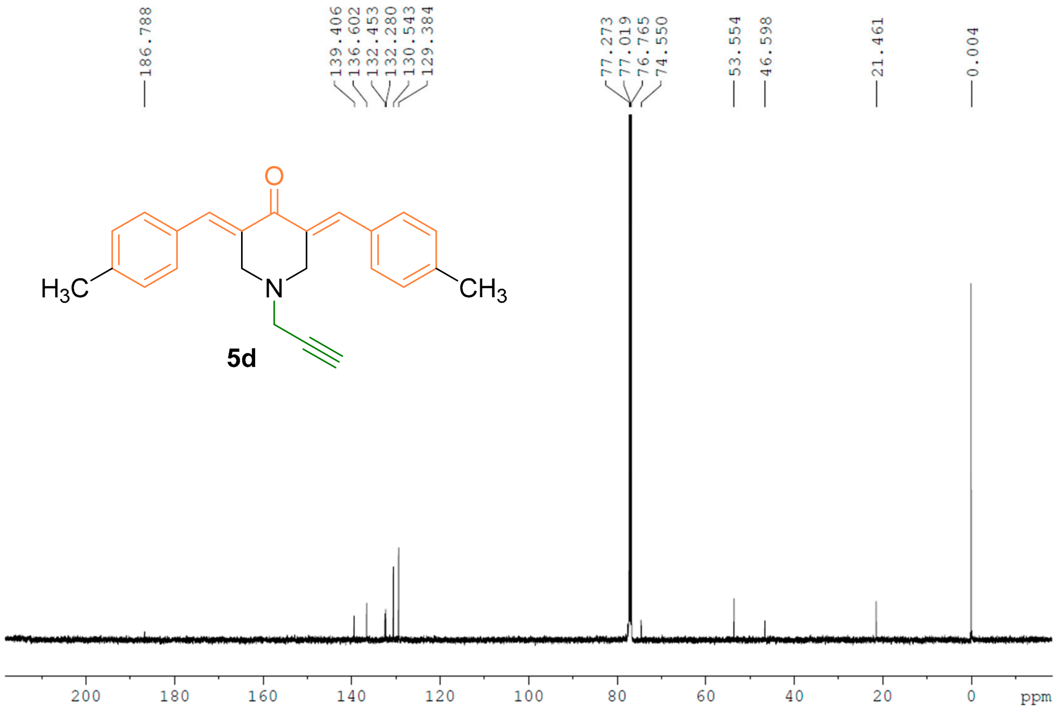

Figure S12. ^{13}C-NMR spectrum of compound **5d**.

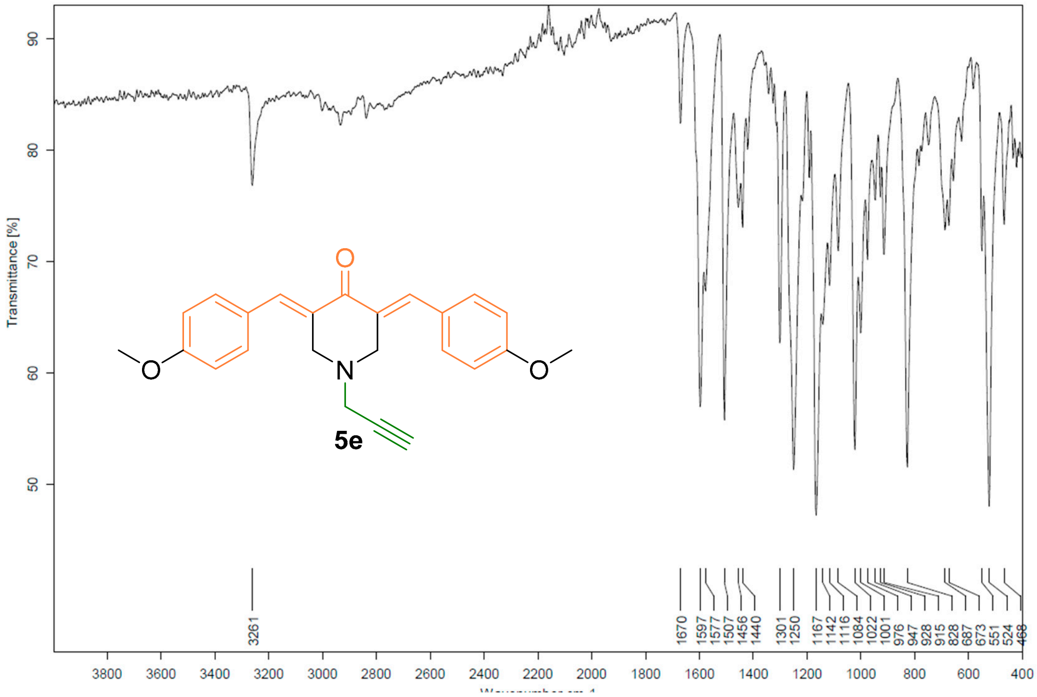

Figure S13. IR spectrum of compound **5e**.

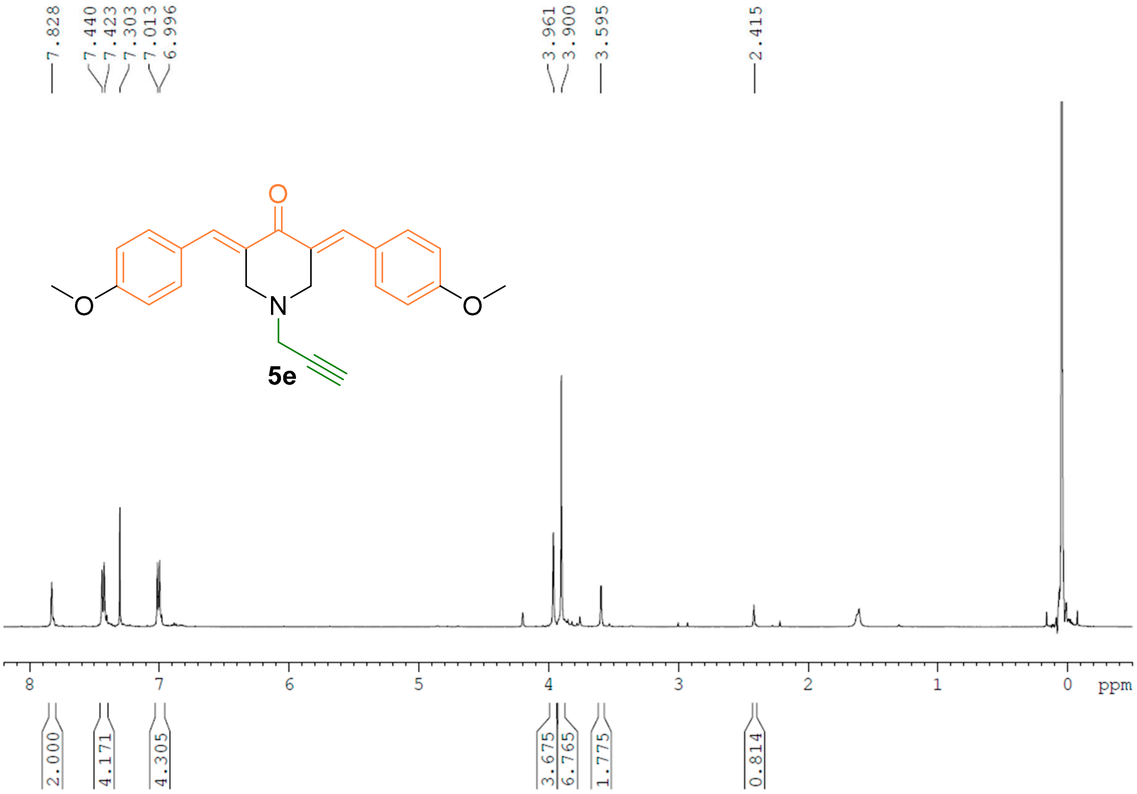

Figure S14. ^{1}H-NMR spectrum of compound **5e**.

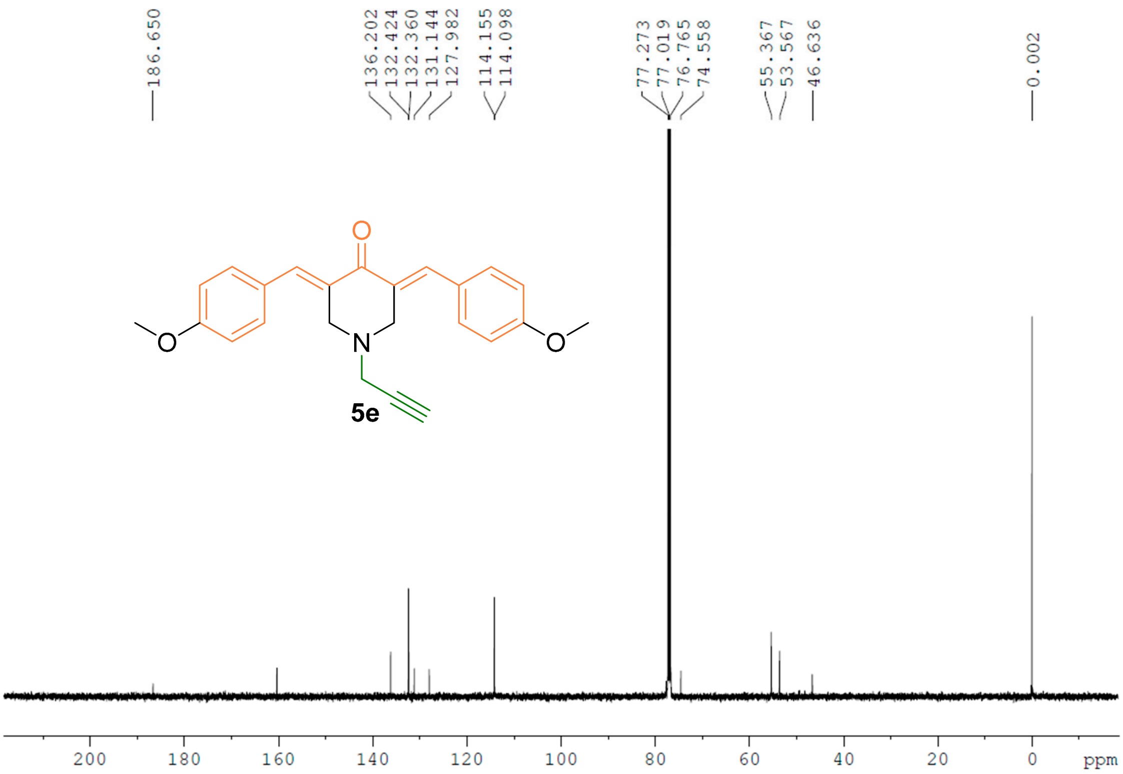

Figure S15. ^{13}C-NMR spectrum of compound **5e**.

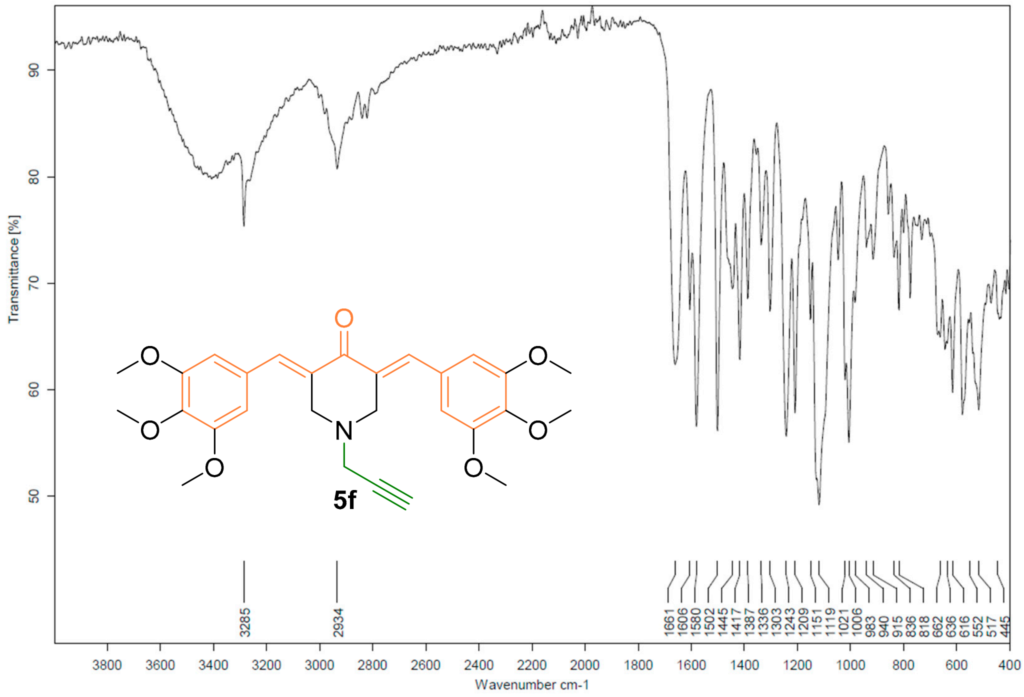

Figure S16. IR spectrum of compound **5f**.

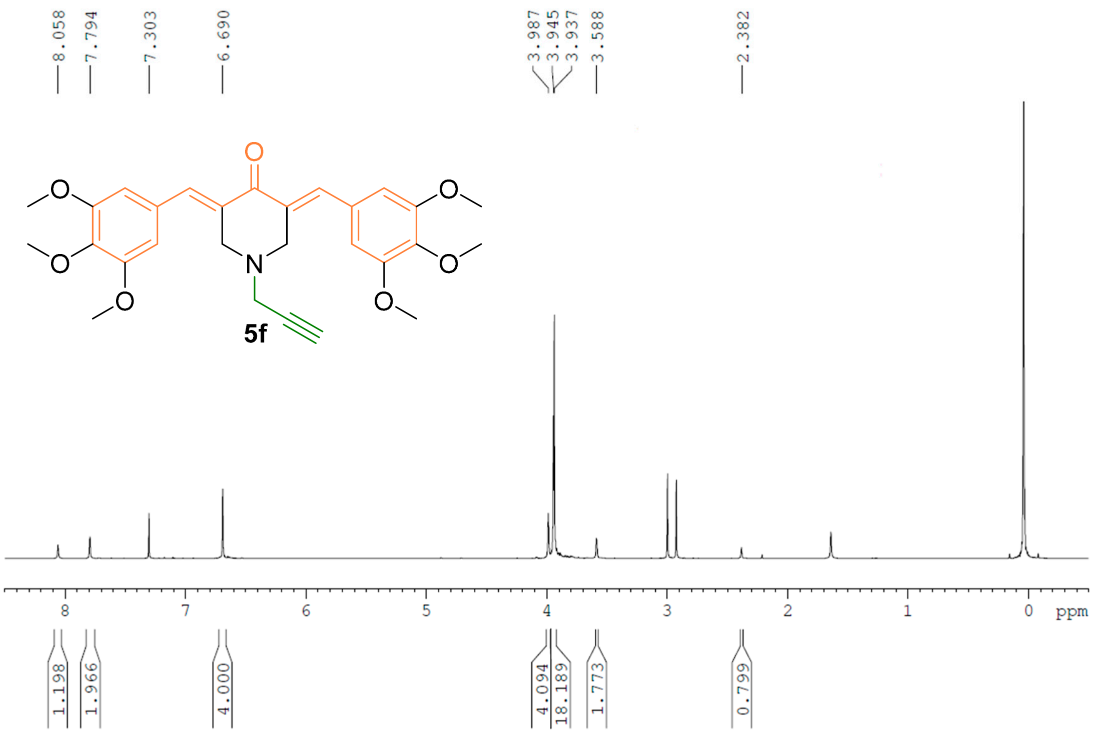

Figure S17. ^{1}H-NMR spectrum of compound **5f**.

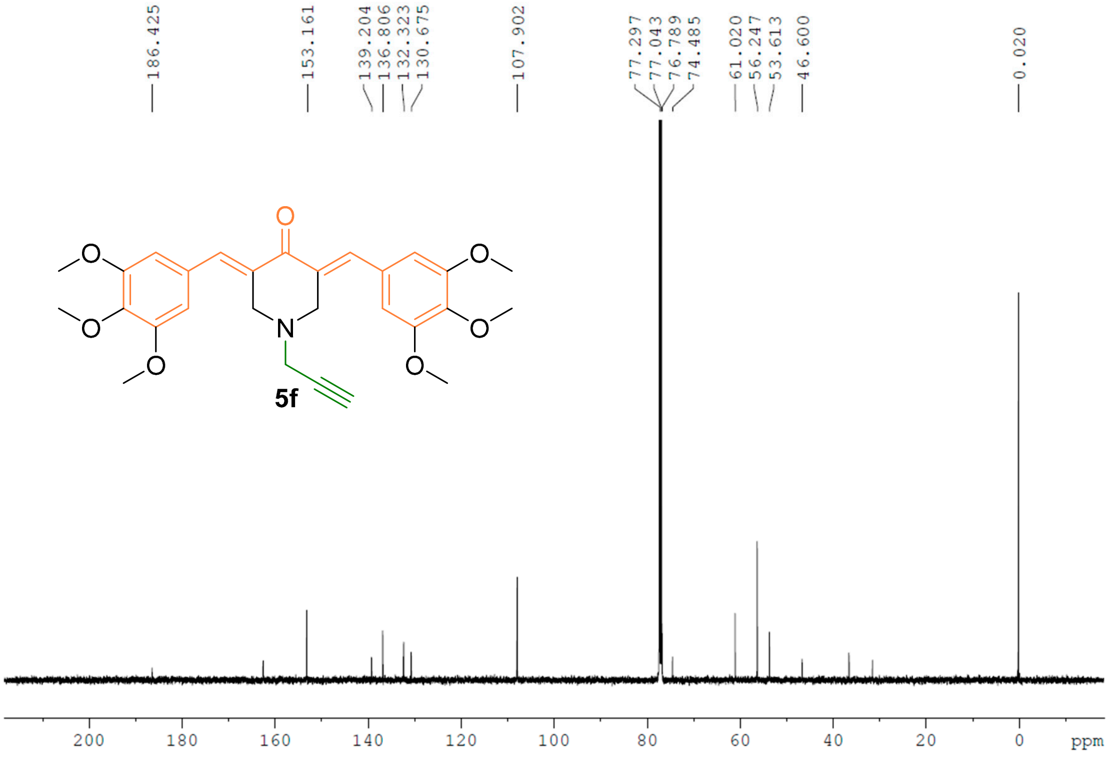

Figure S18. ^{13}C-NMR spectrum of compound **5f**.

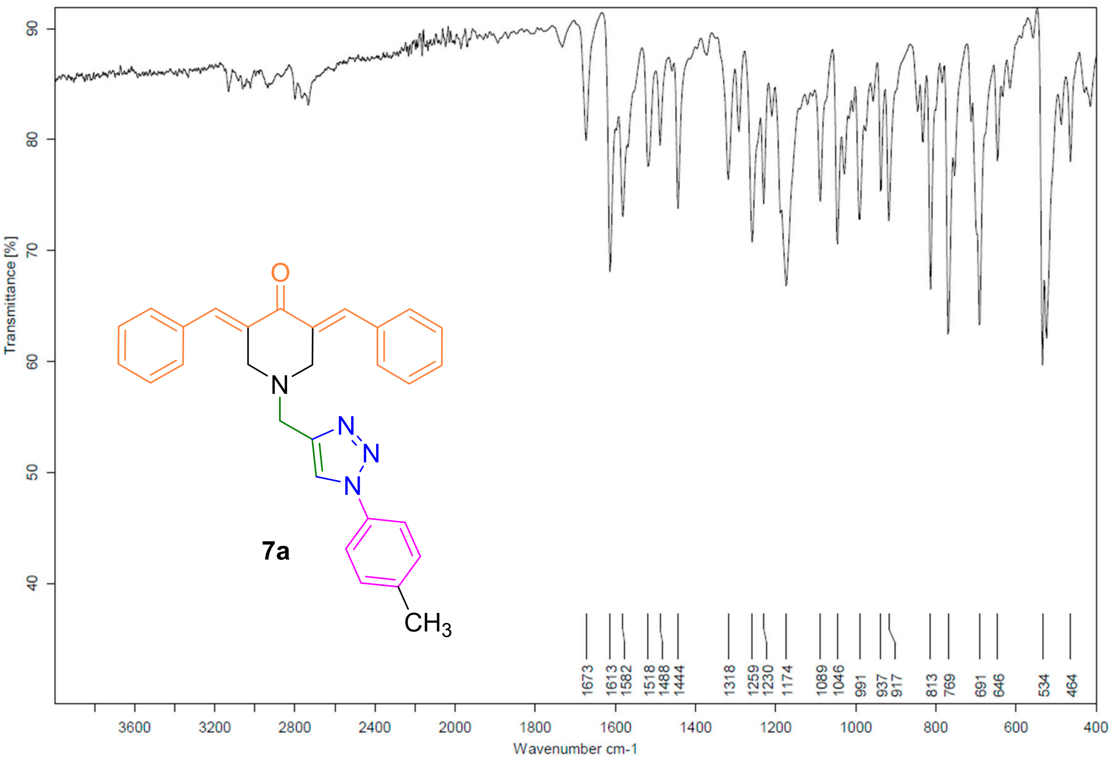

Figure S19. IR spectrum of compound **7a**.

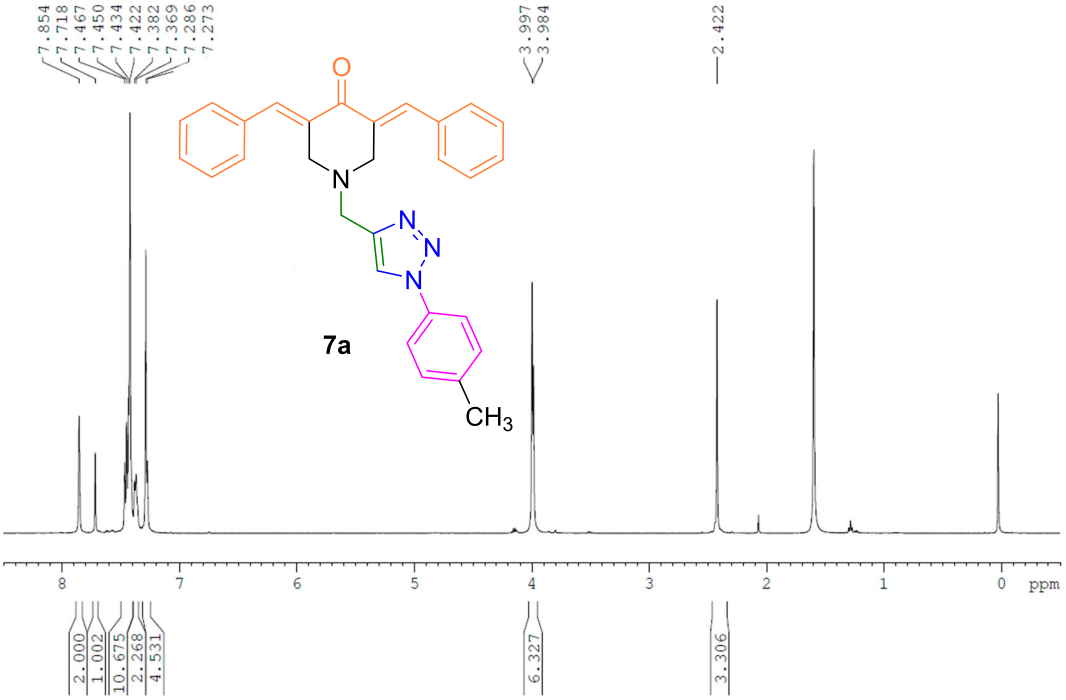

Figure S20. ^{1}H-NMR spectrum of compound **7a**.

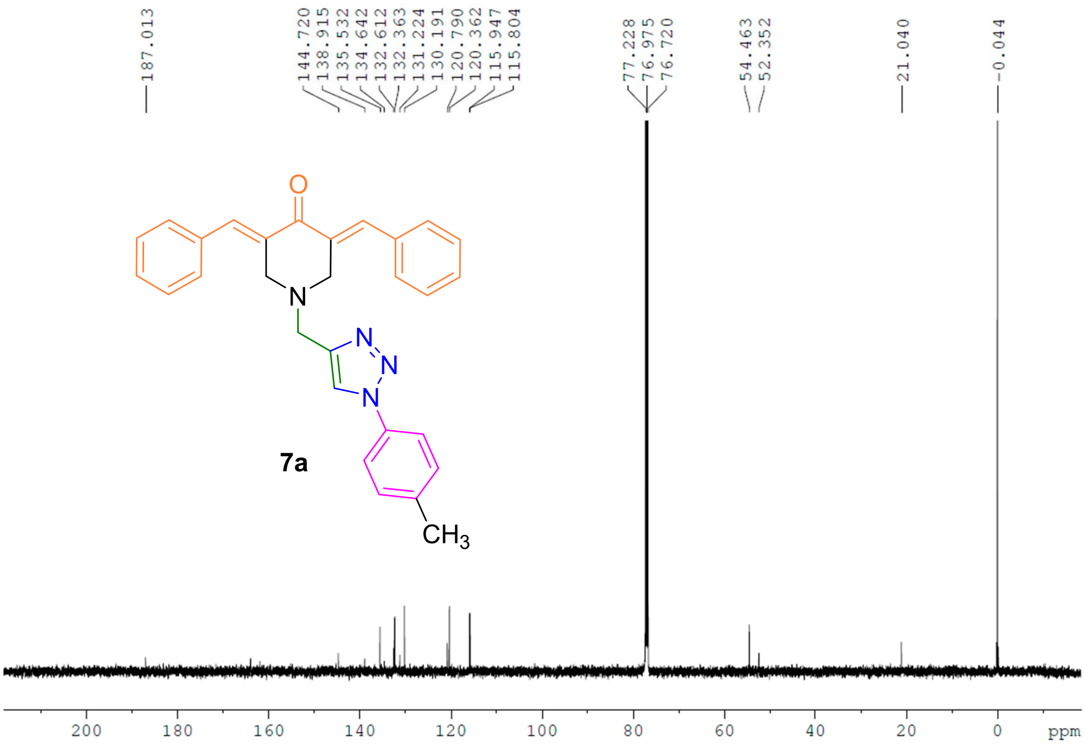

Figure S21. ^{13}C-NMR spectrum of compound **7a**.

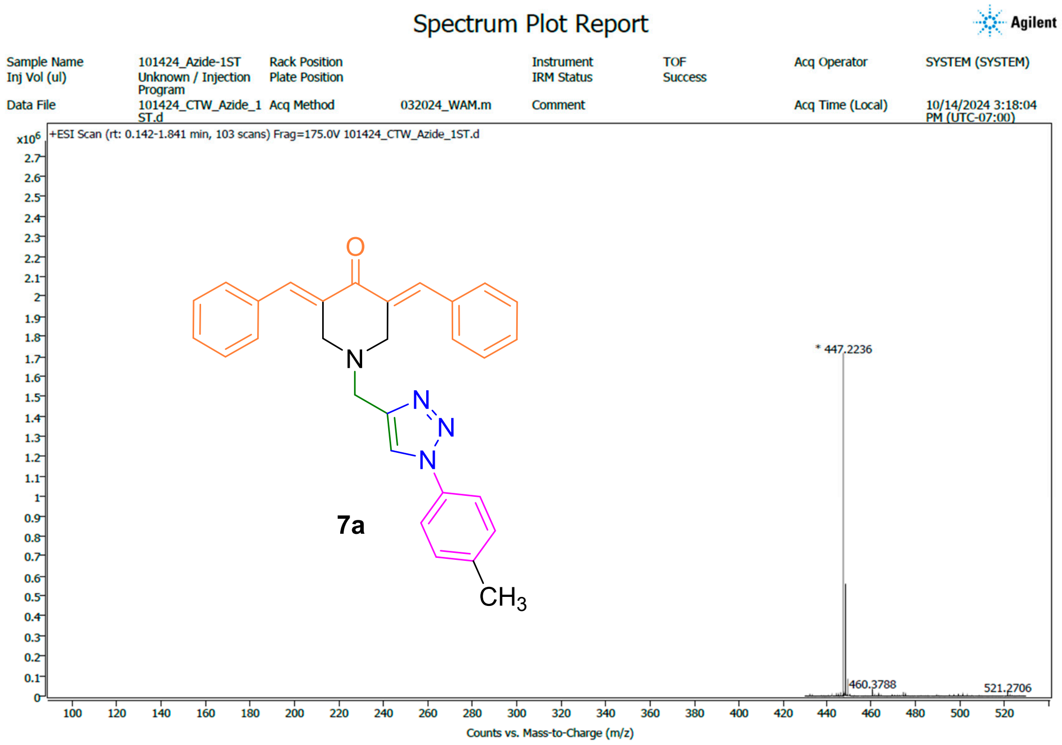

Figure S22. Mass spectrum of compound **7a**.

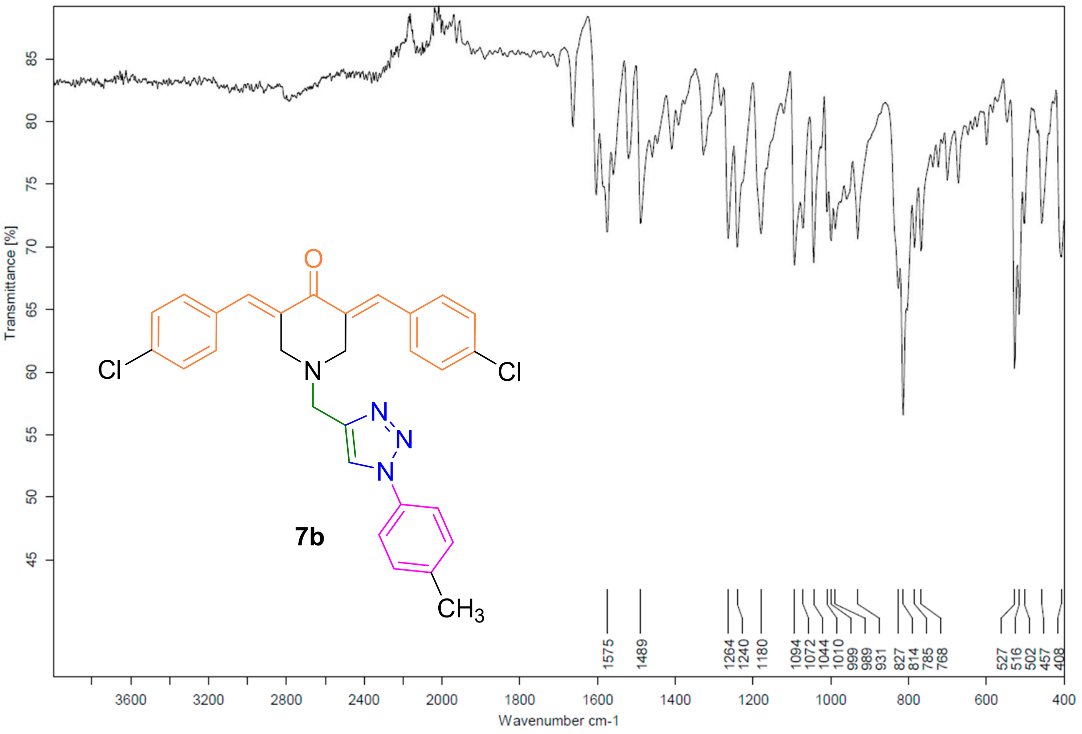

Figure S23. IR spectrum of compound **7b**.

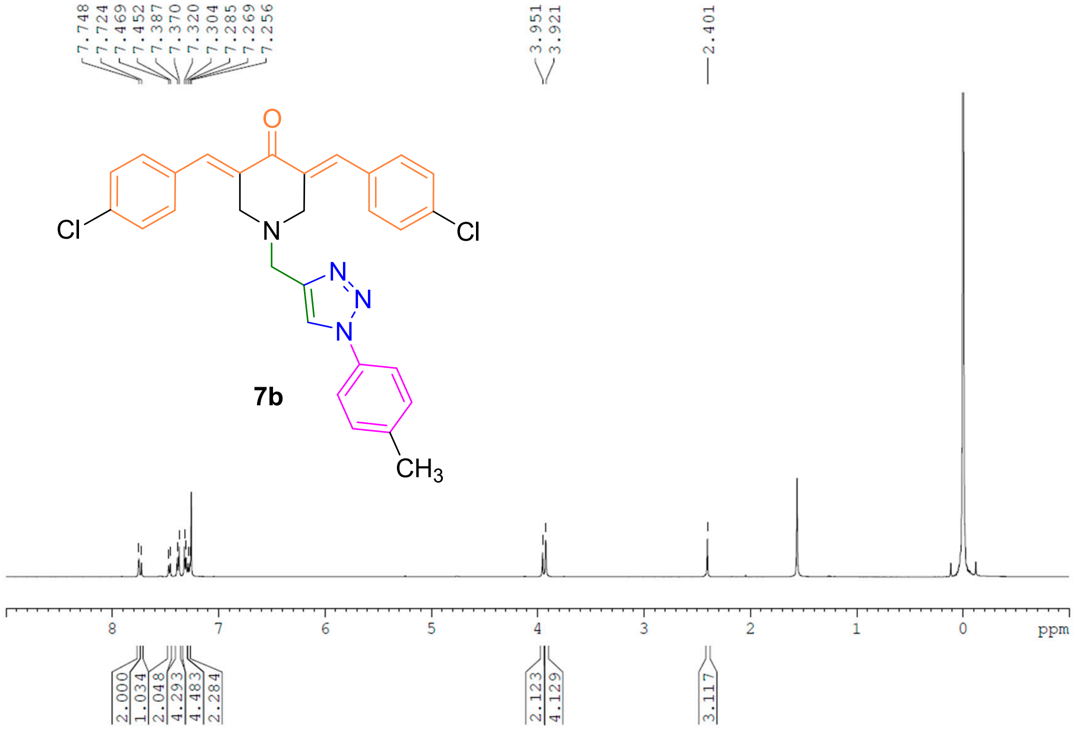

Figure S24. ^{1}H-NMR spectrum of compound **7b**.

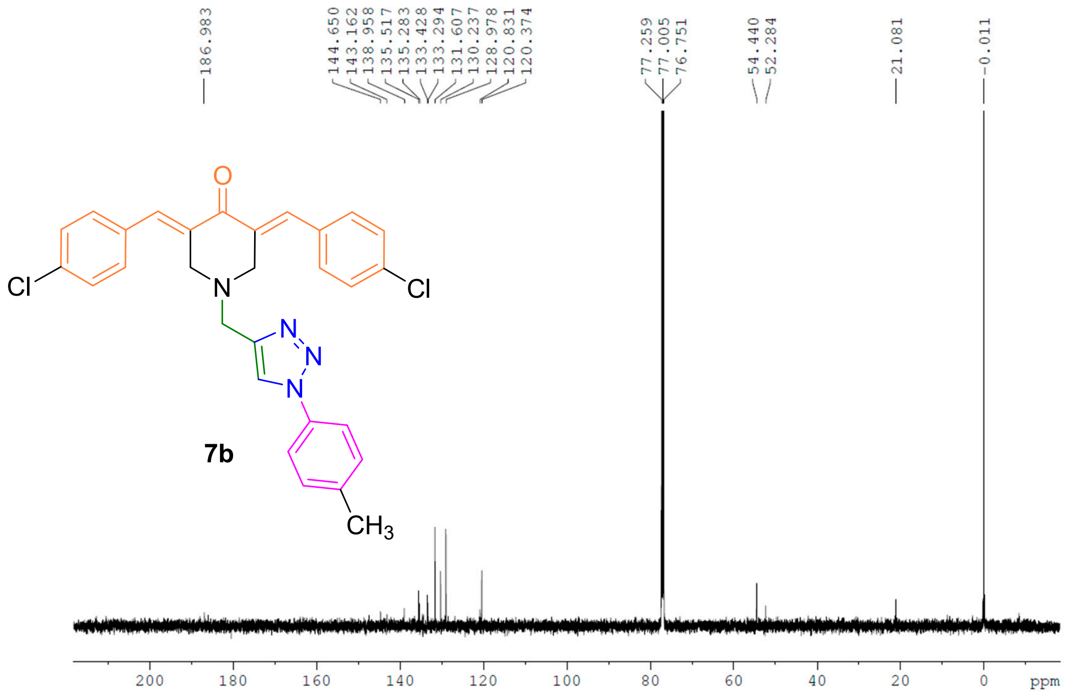

Figure S25. ^{13}C-NMR spectrum of compound **7b**.

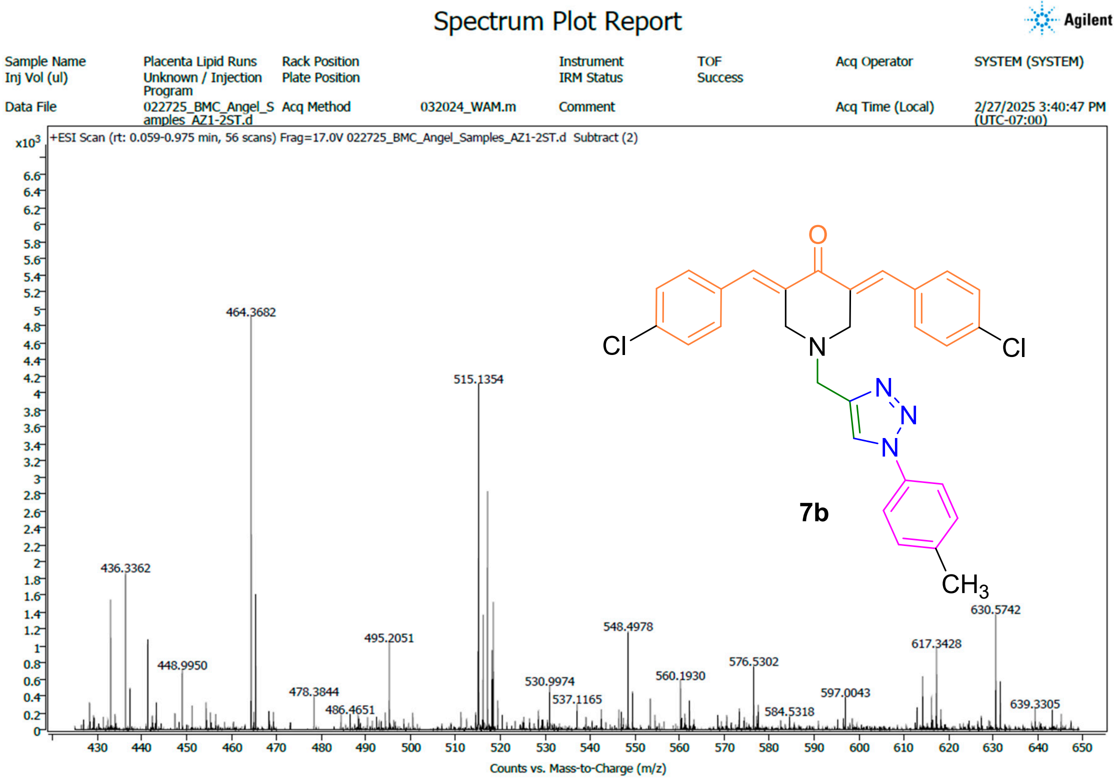

Figure S26. Mass spectrum of compound **7b**.

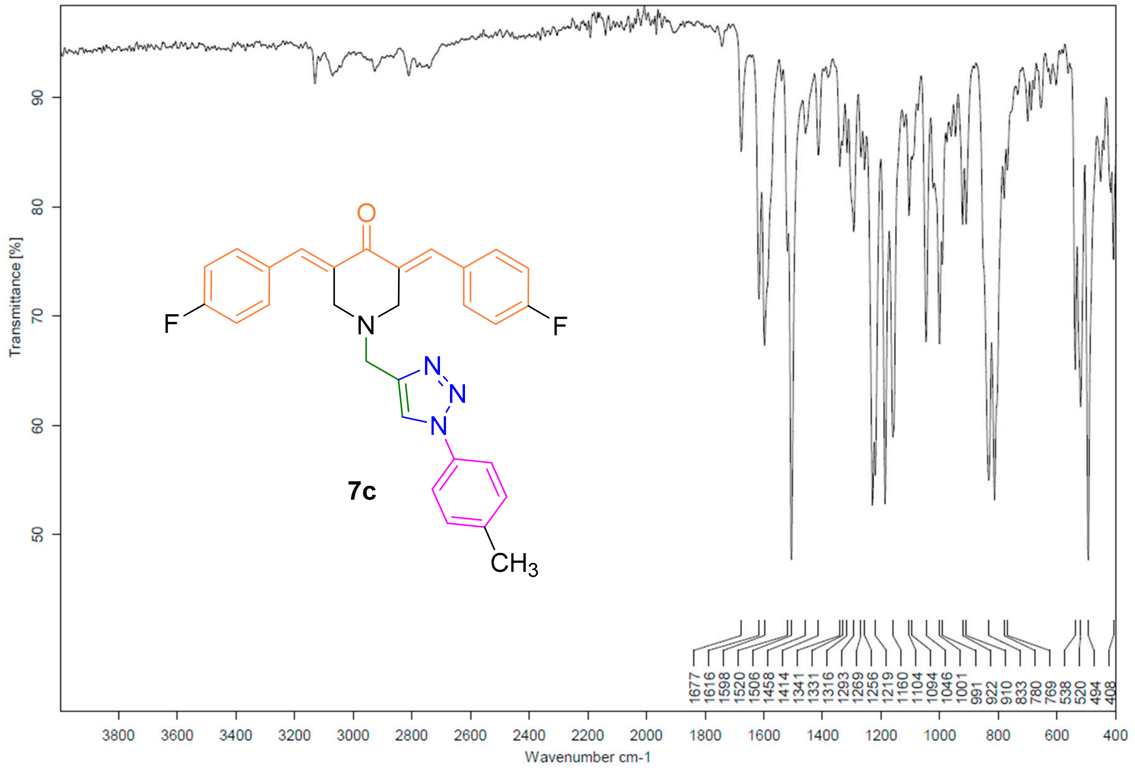

Figure S27. IR spectrum of compound **7c**.

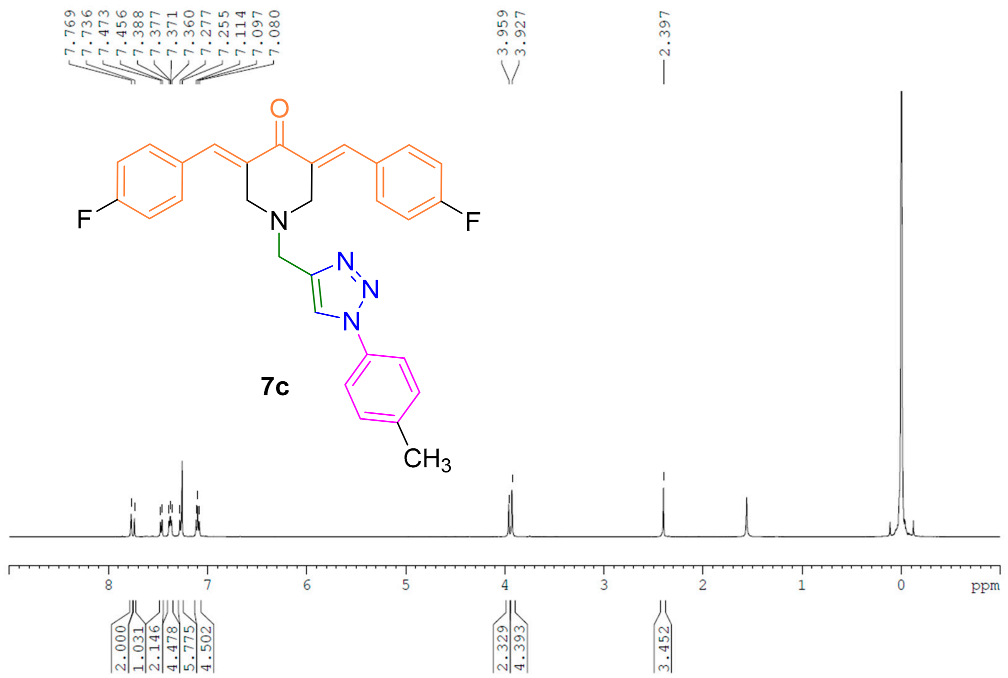

Figure S28. ^{1}H-NMR spectrum of compound **7c**.

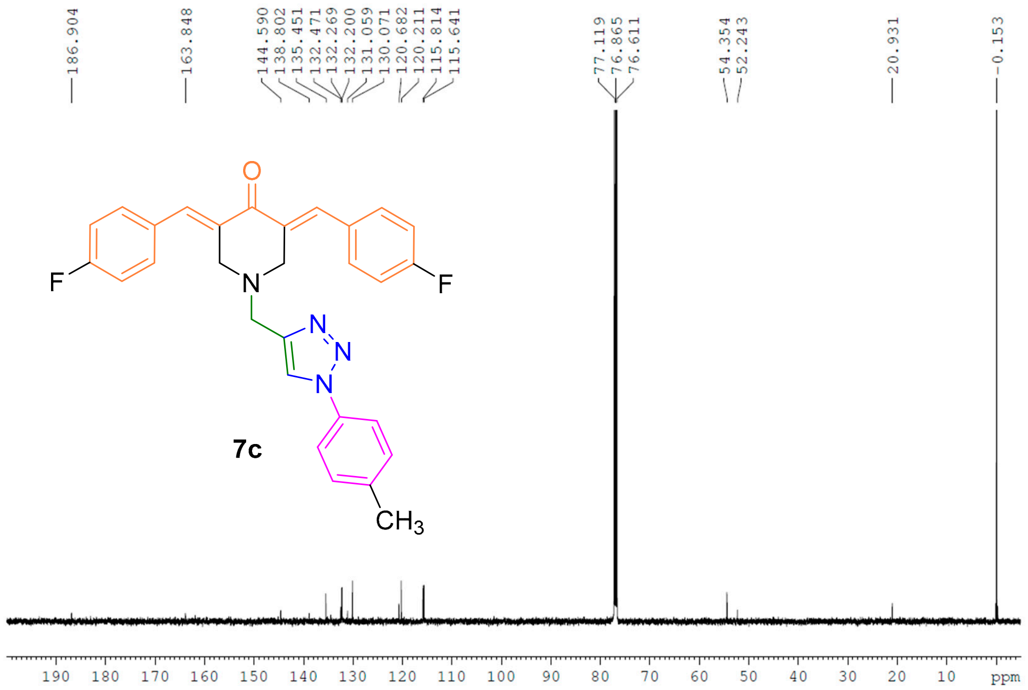

Figure S29. ^{13}C-NMR spectrum of compound **7c**.

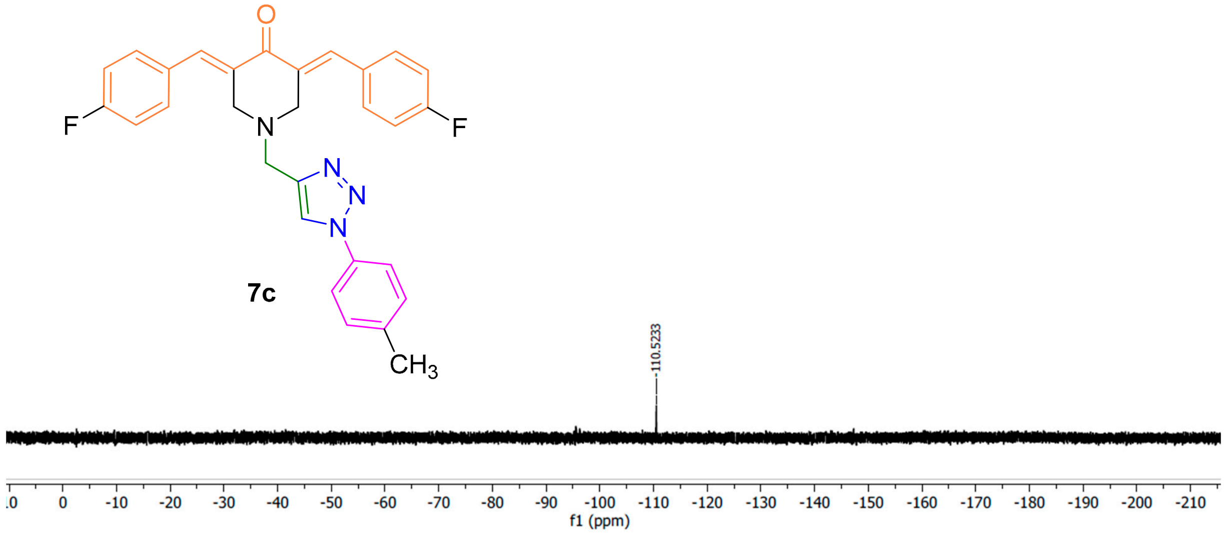

Figure S30. ^{19}F spectrum of compound **7c**.

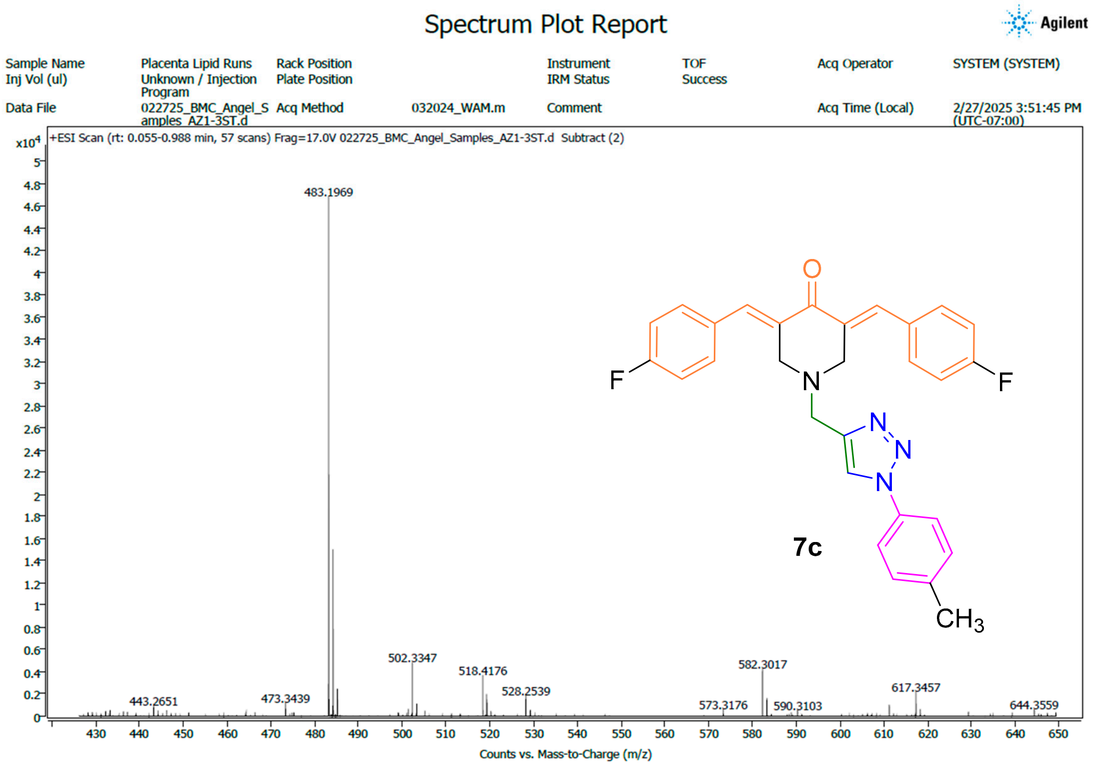

Figure S31. Mass spectrum of compound **7c**.

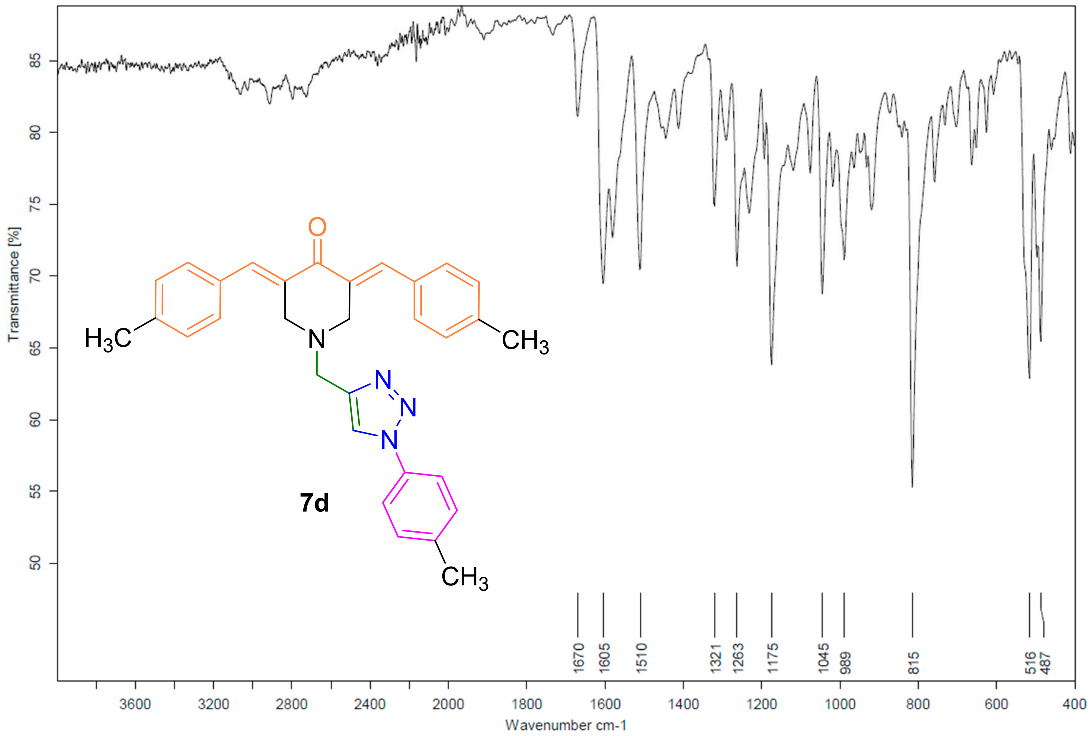

Figure S32. IR spectrum of compound **7d**.

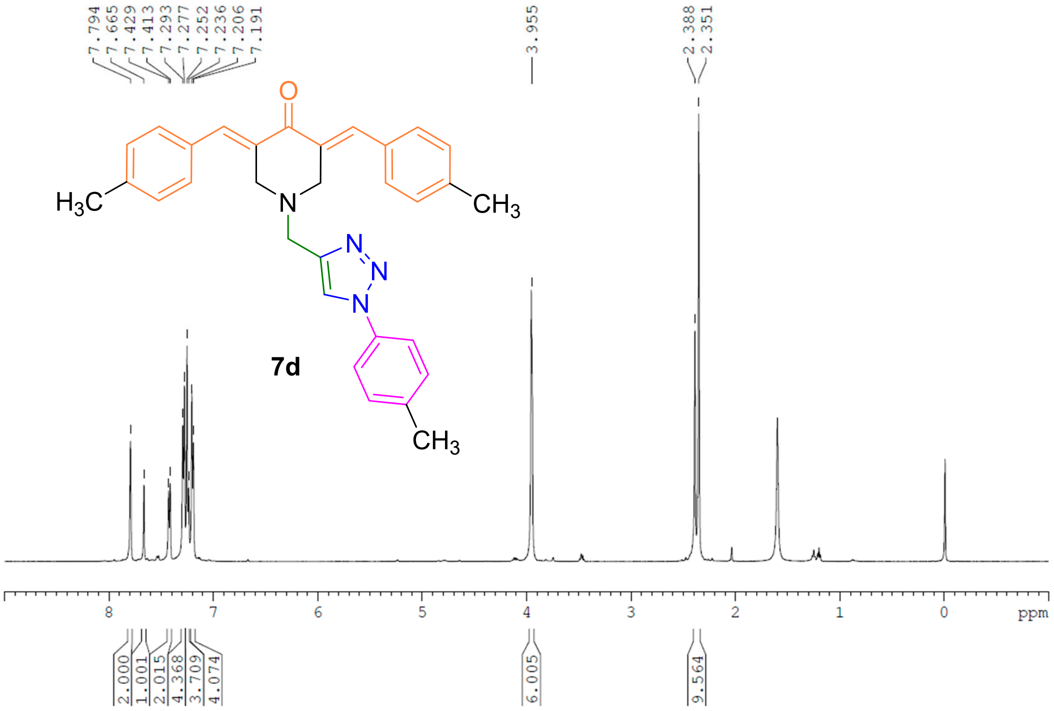

Figure S33. ^{1}H-NMR spectrum of compound **7d**.

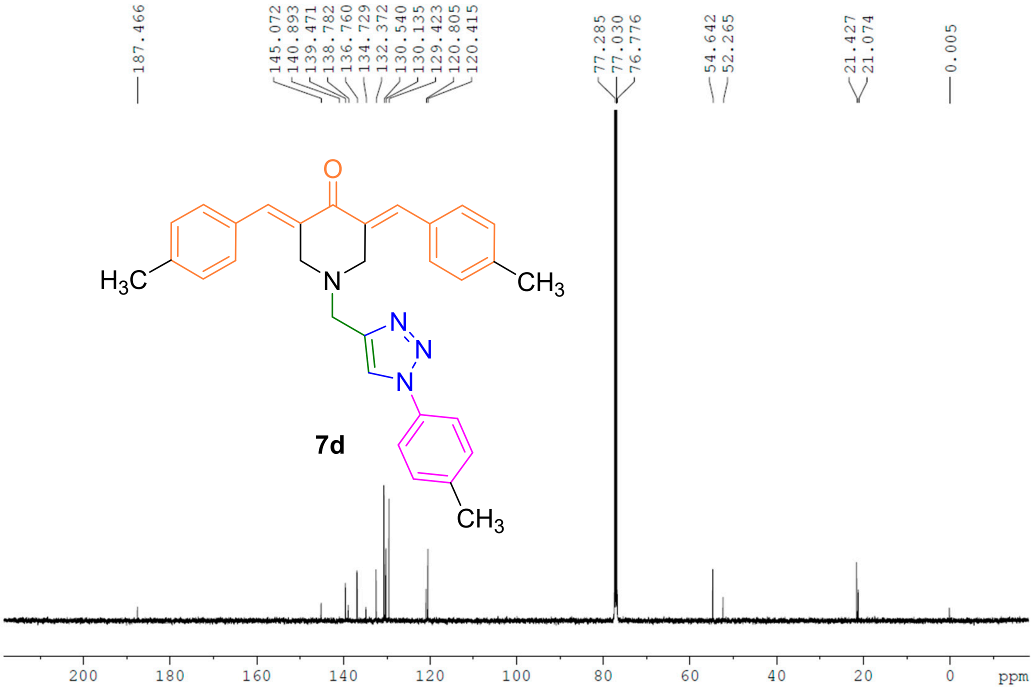

Figure S34. ^{13}C-NMR spectrum of compound **7d**.

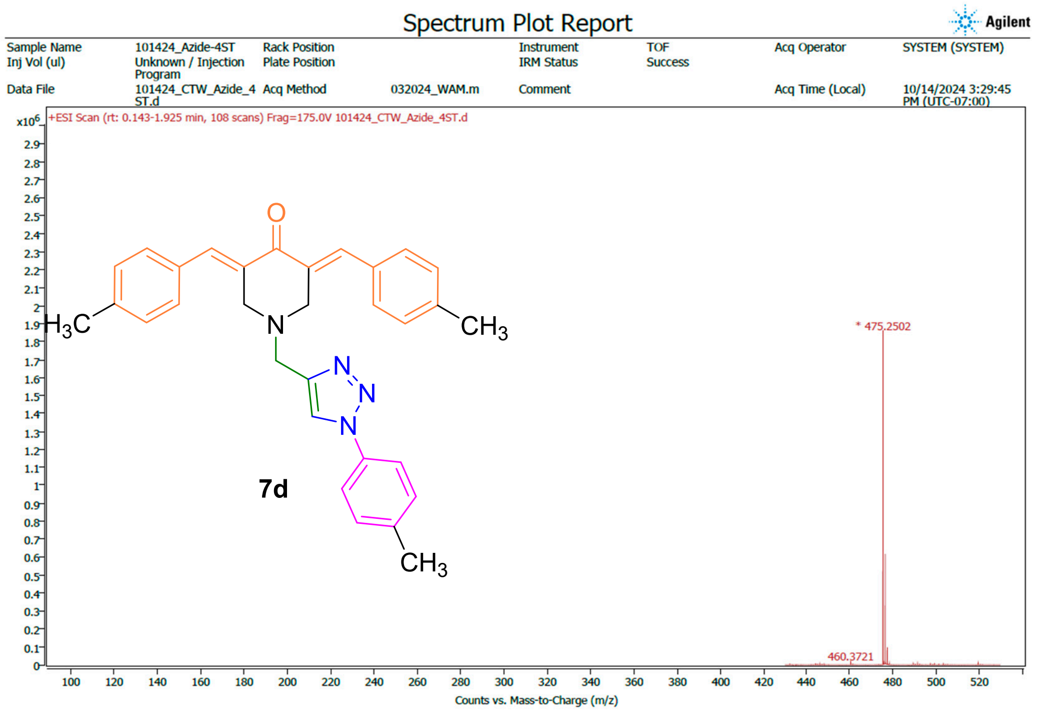

Figure S35. Mass spectrum of compound **7d**.

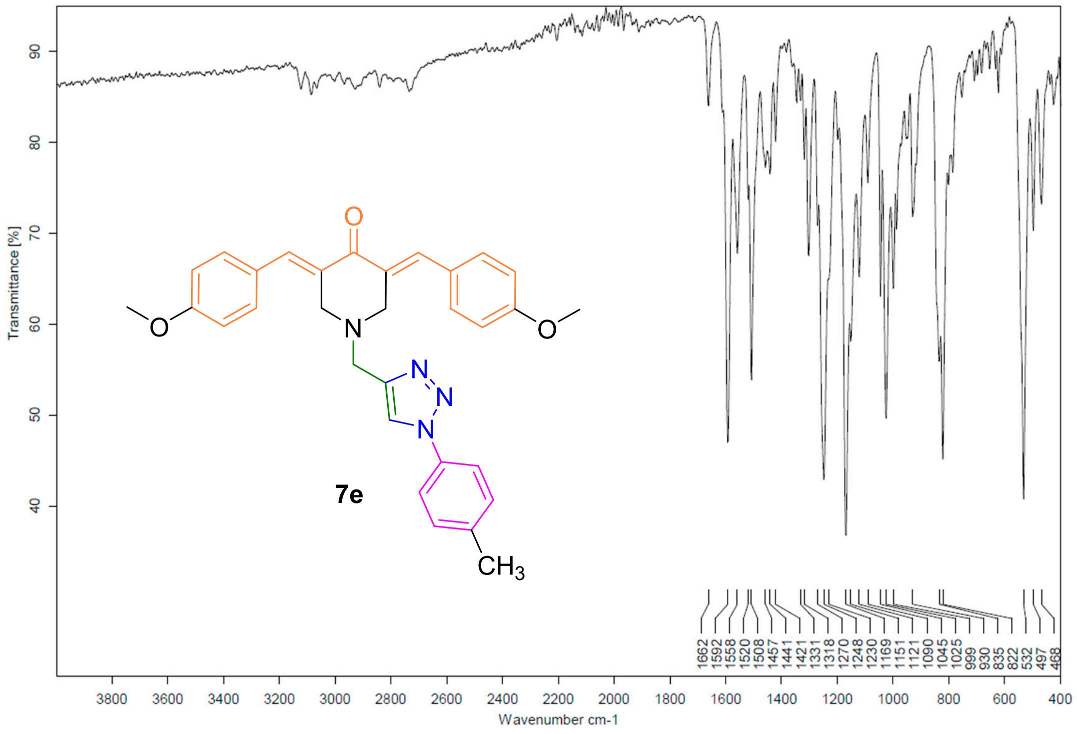

Figure S36. IR spectrum of compound **7e**.

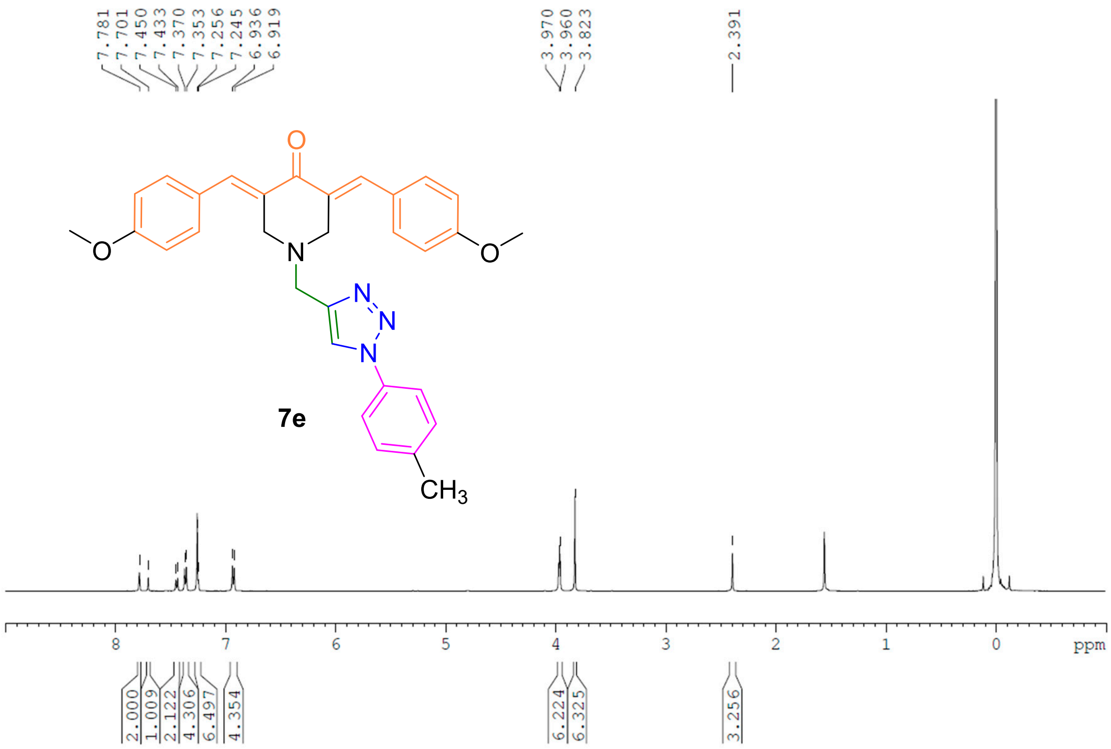

Figure S37. ^{1}H-NMR spectrum of compound **7e**.

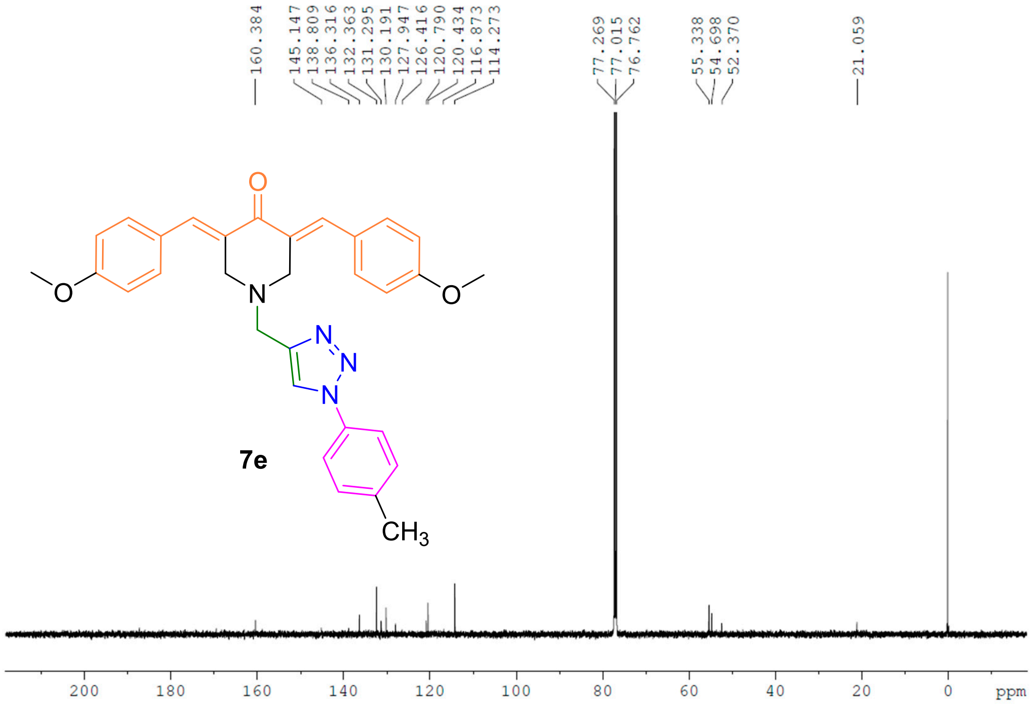

Figure S38. ^{13}C-NMR spectrum of compound **7e**.

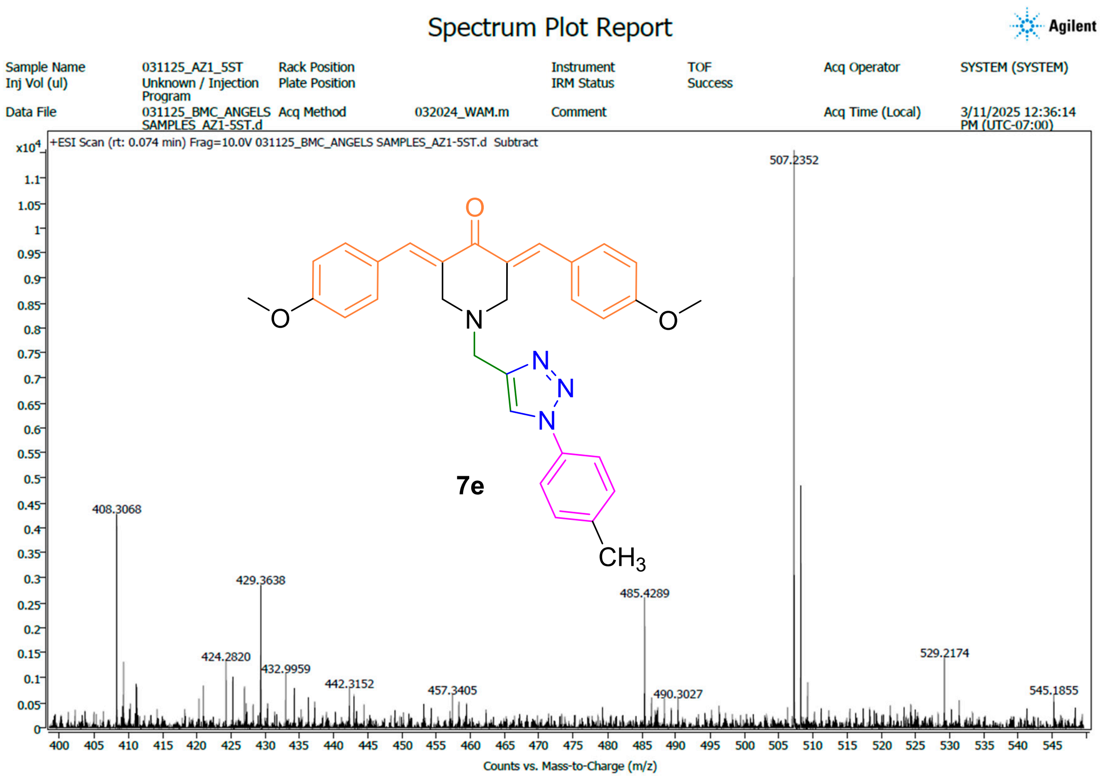

Figure S39. Mass spectrum of compound **7e**.

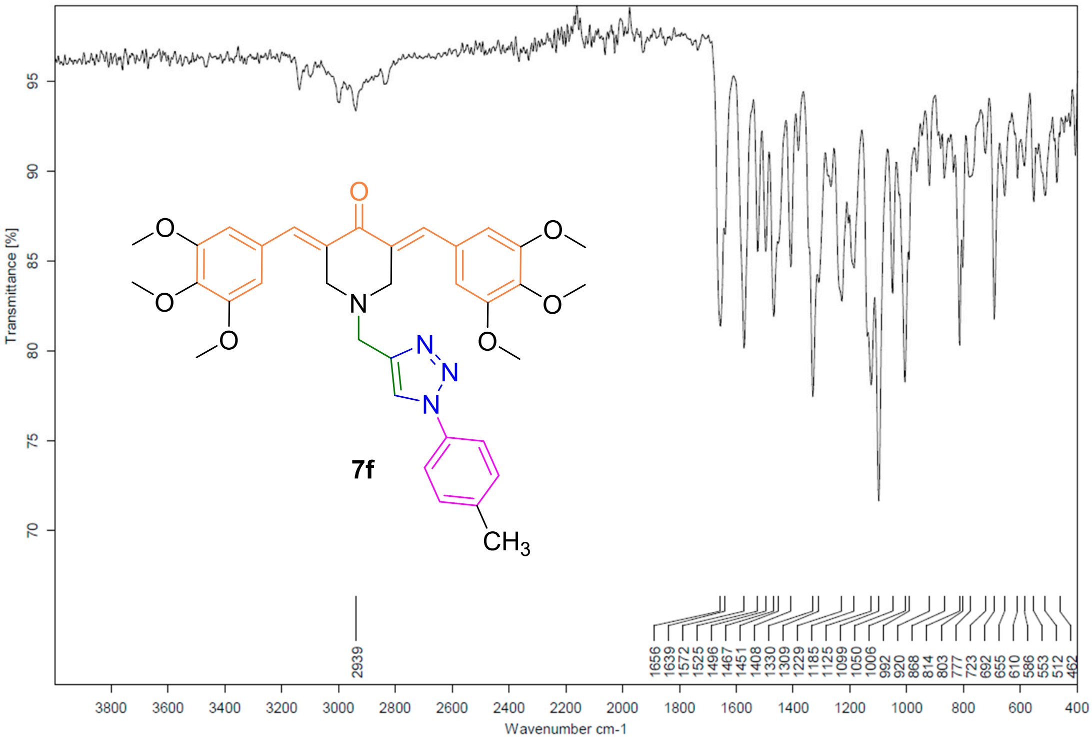

Figure S40. IR spectrum of compound **7f**.

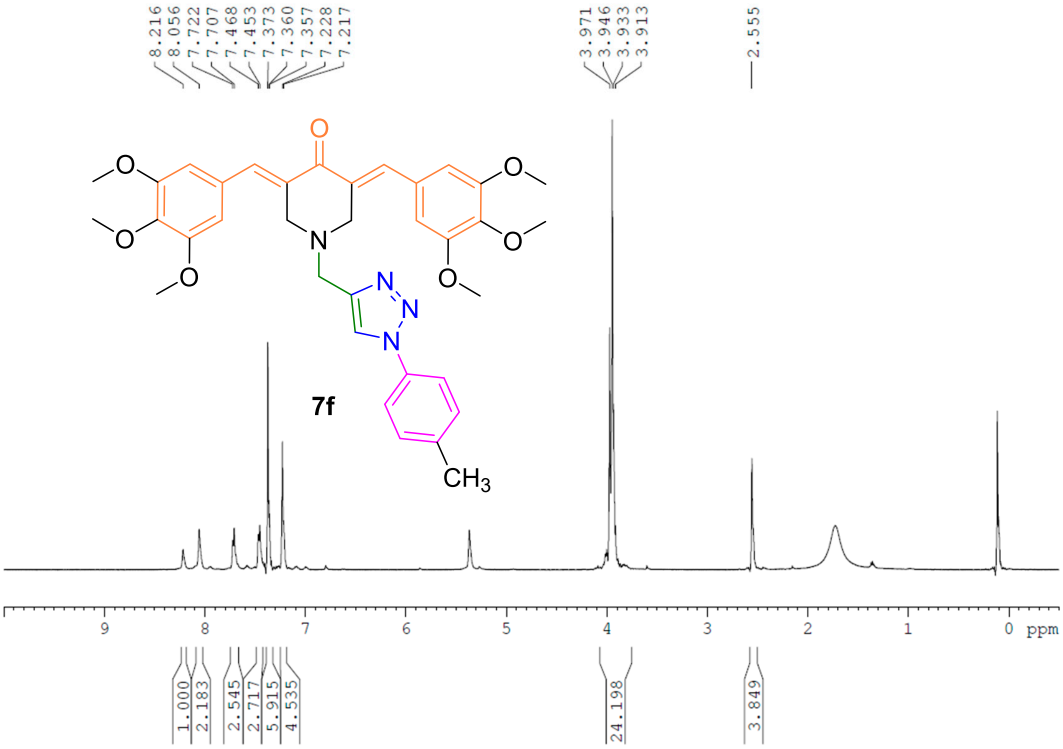

Figure S41. ^{1}H-NMR spectrum of compound **7f**.

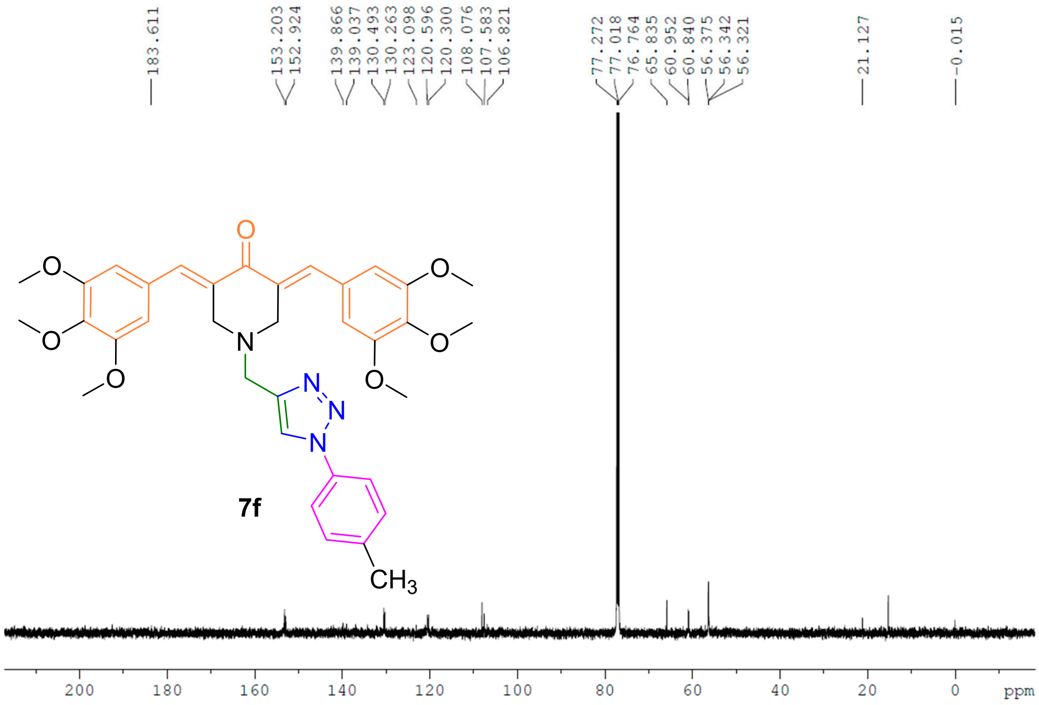

Figure S42. ^{13}C-NMR spectrum of compound **7f**.

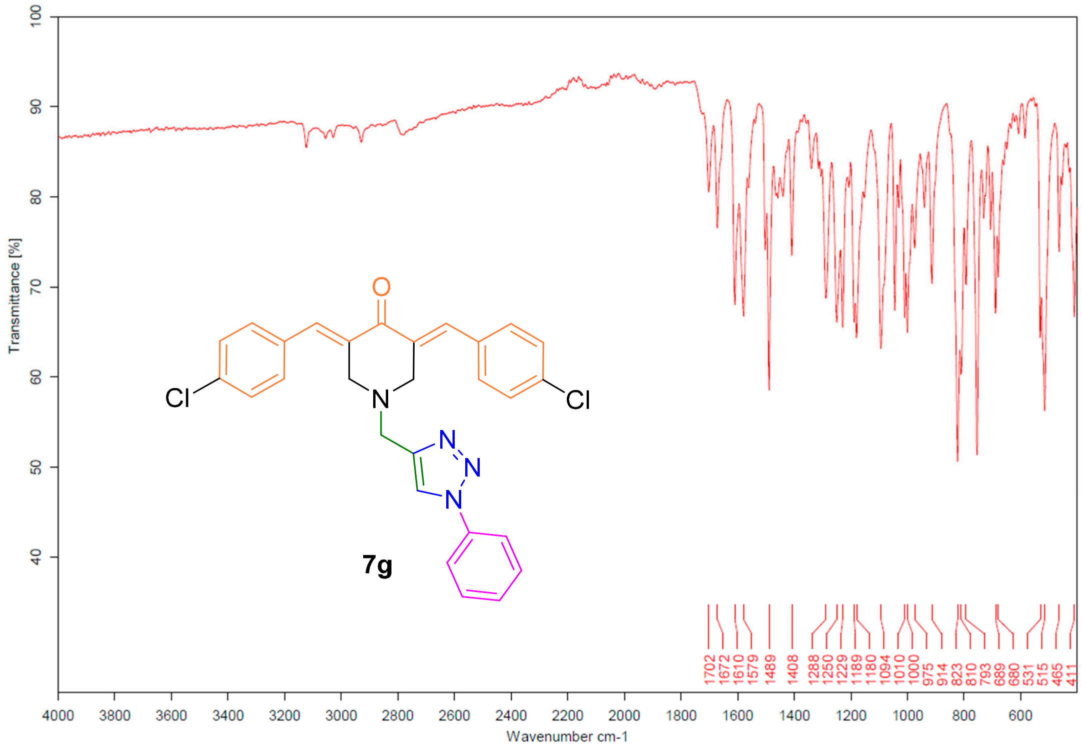

Figure S43. IR spectrum of compound **7g**.

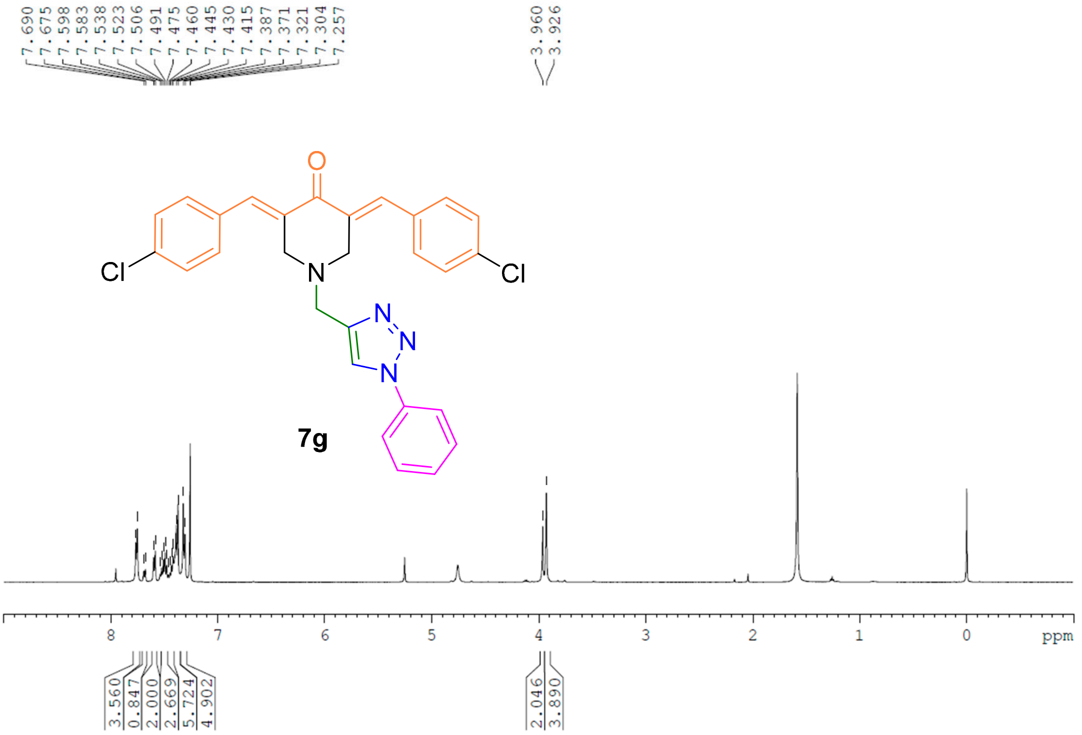

Figure S44. ^{1}H-NMR spectrum of compound **7g**.

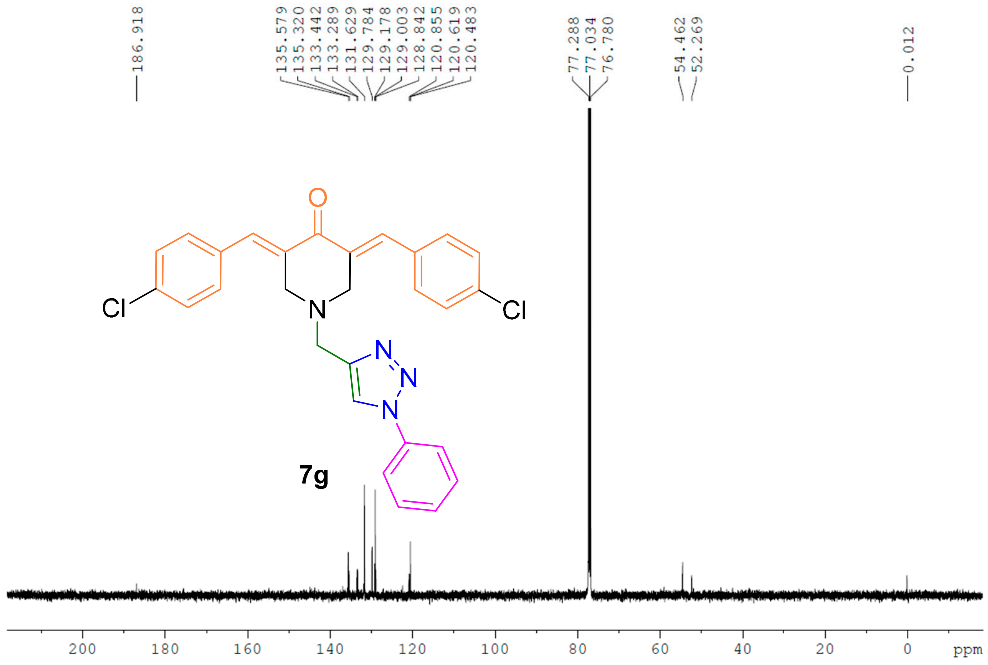

Figure S45. ^{13}C-NMR spectrum of compound **7g**.

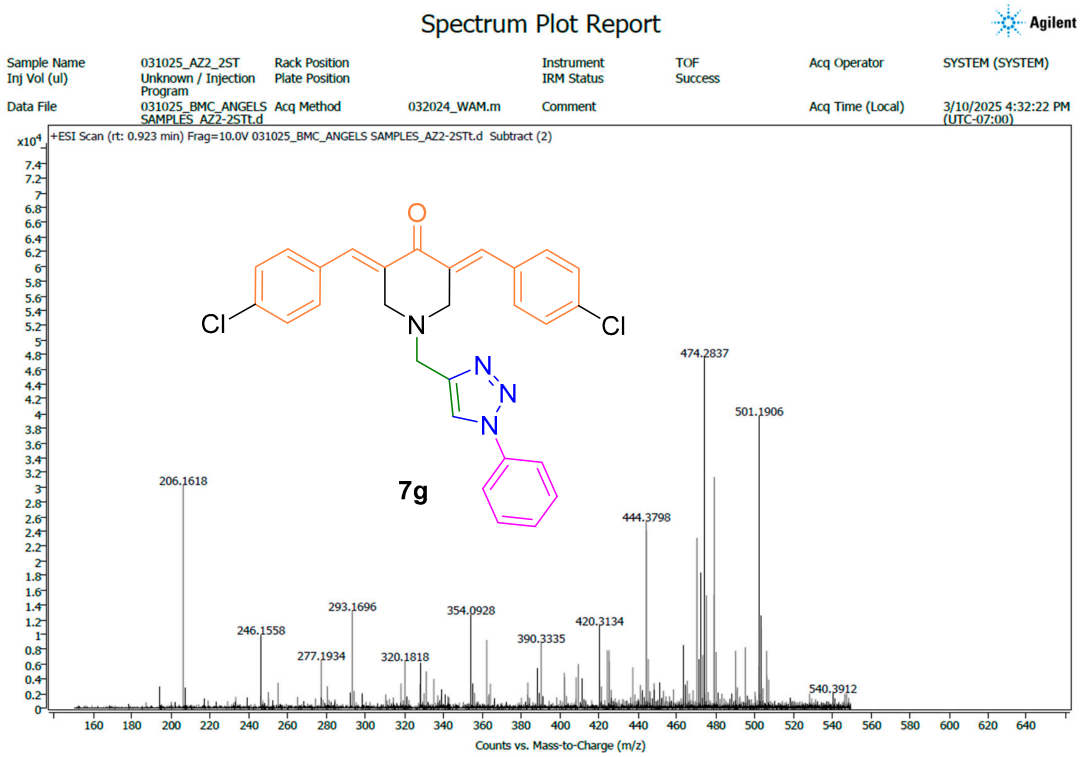

Figure S46. Mass spectrum of compound **7g**.

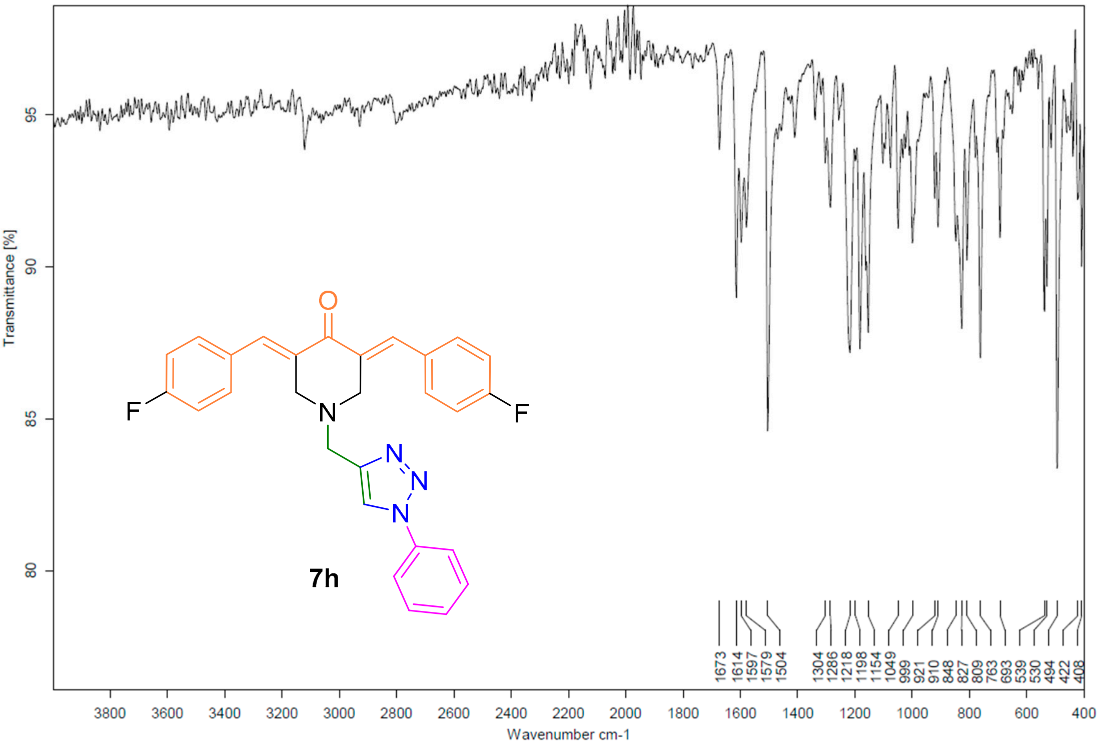

Figure S47. IR spectrum of compound **7h**.

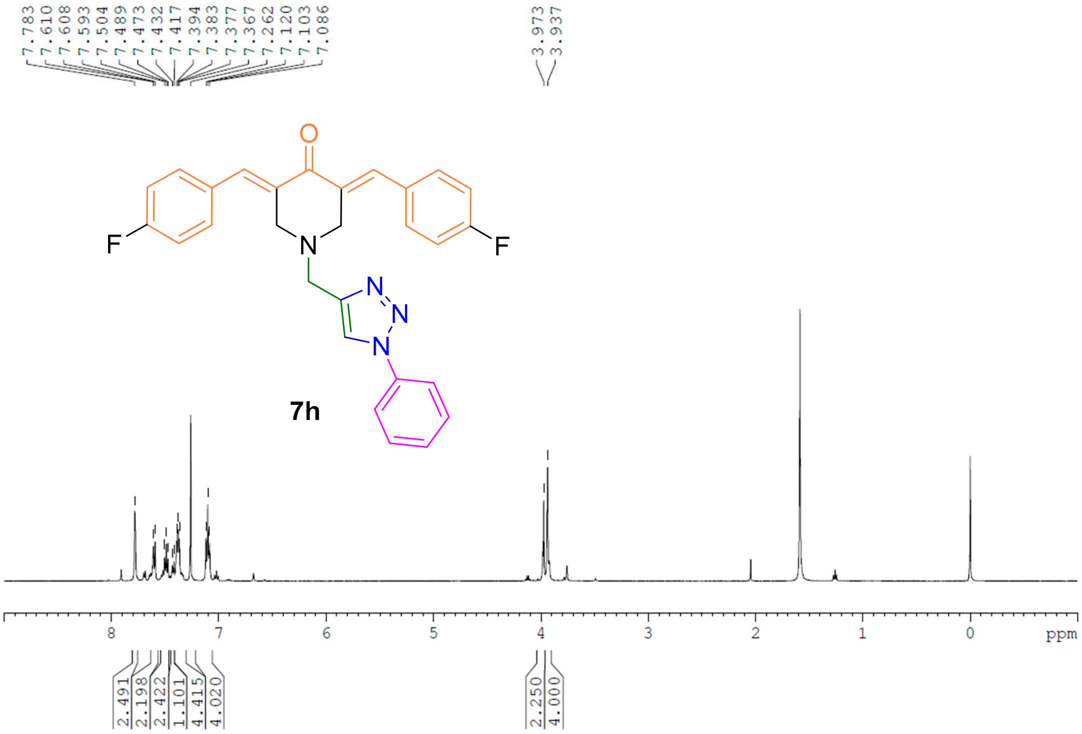

Figure S48. ^{1}H-NMR spectrum of compound **7h**.

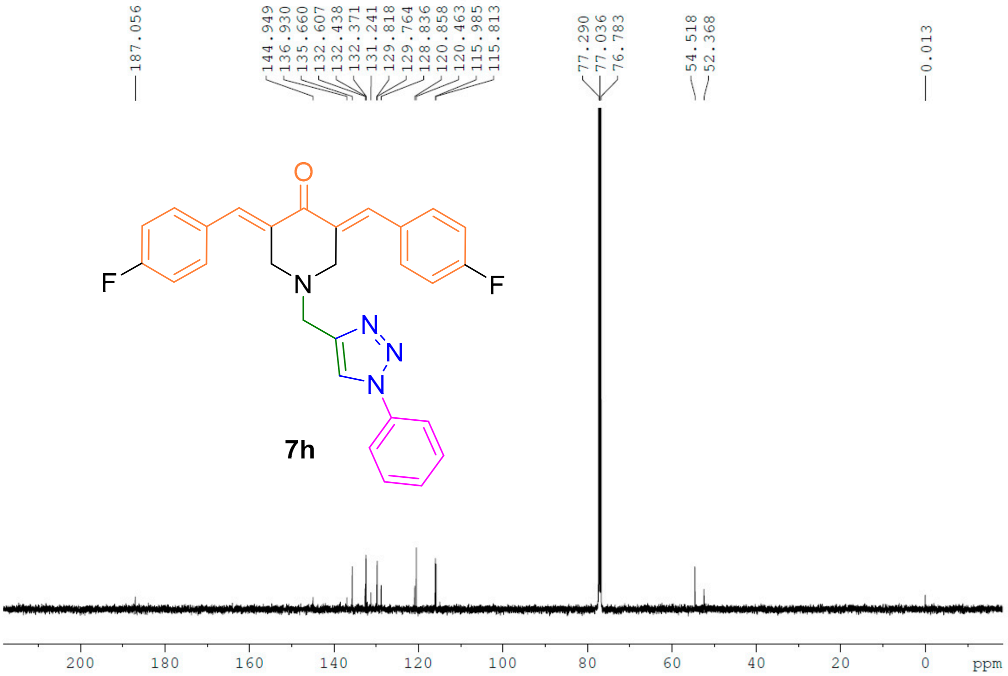

Figure S49. ^{13}C-NMR spectrum of compound **7h**.

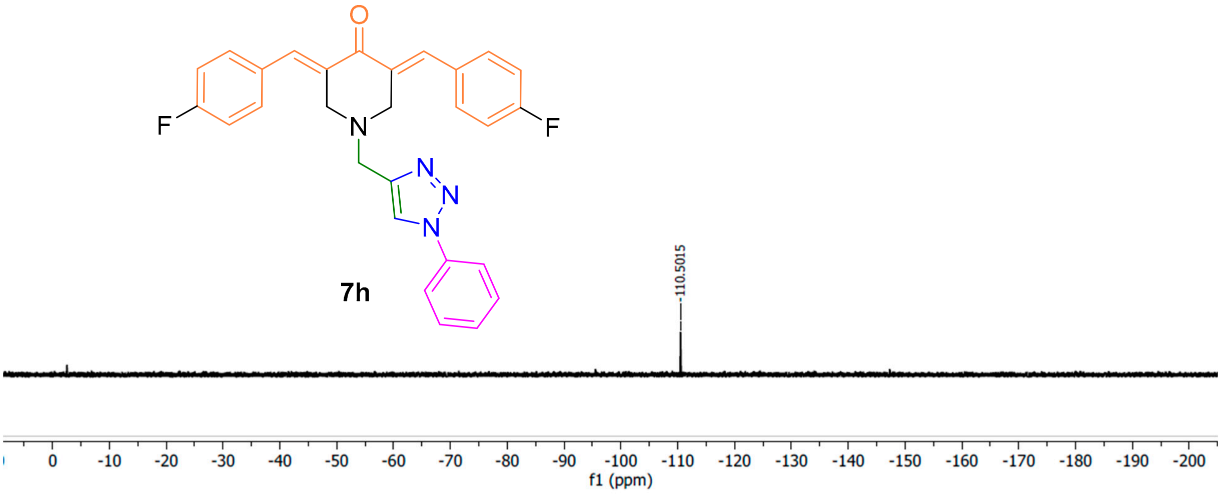

Figure S50. ^{19}F spectrum of compound **7h**.

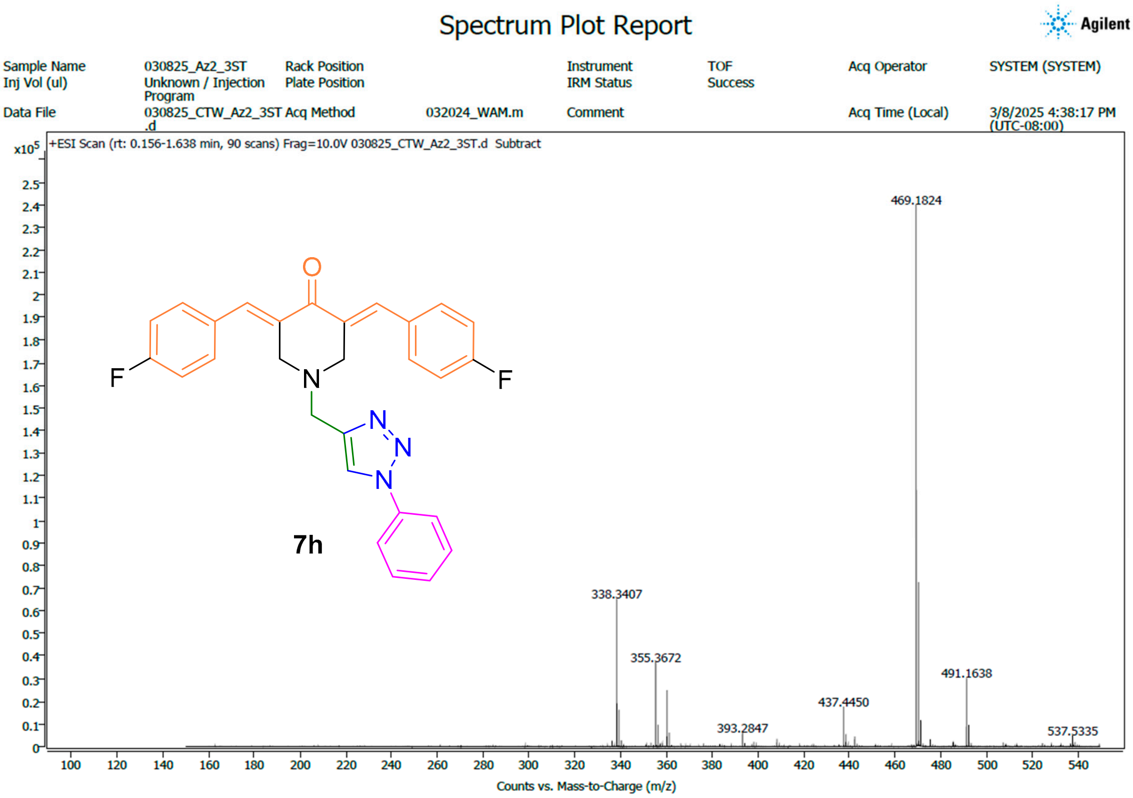

Figure S51. Mass spectrum of compound **7h**.

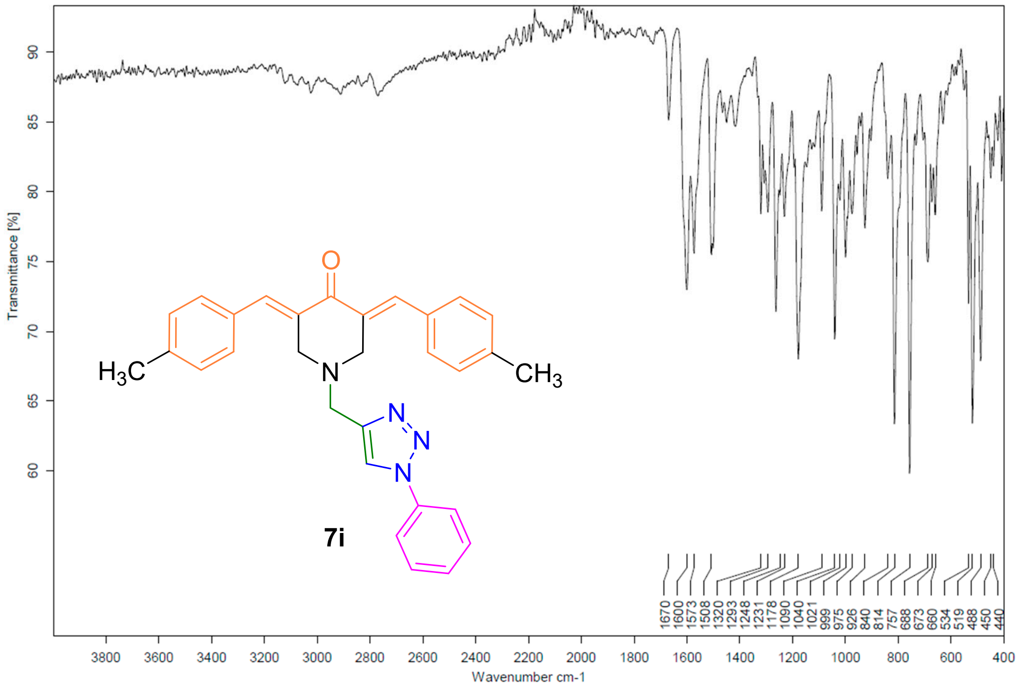

Figure S52. IR spectrum of compound **7i**.

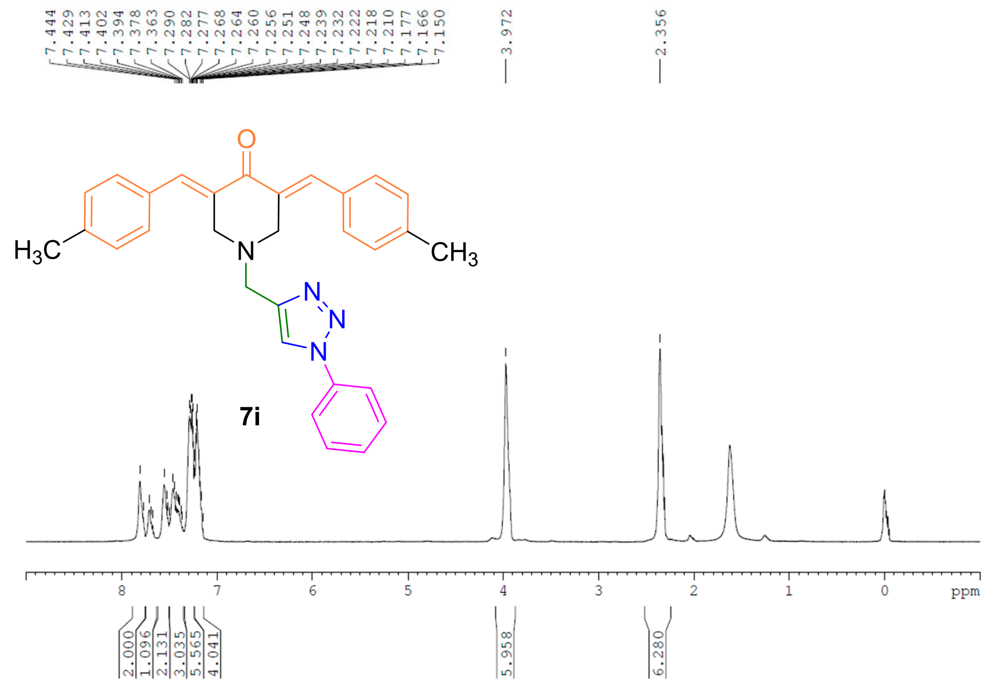

Figure S53. ^{1}H-NMR spectrum of compound **7i**.

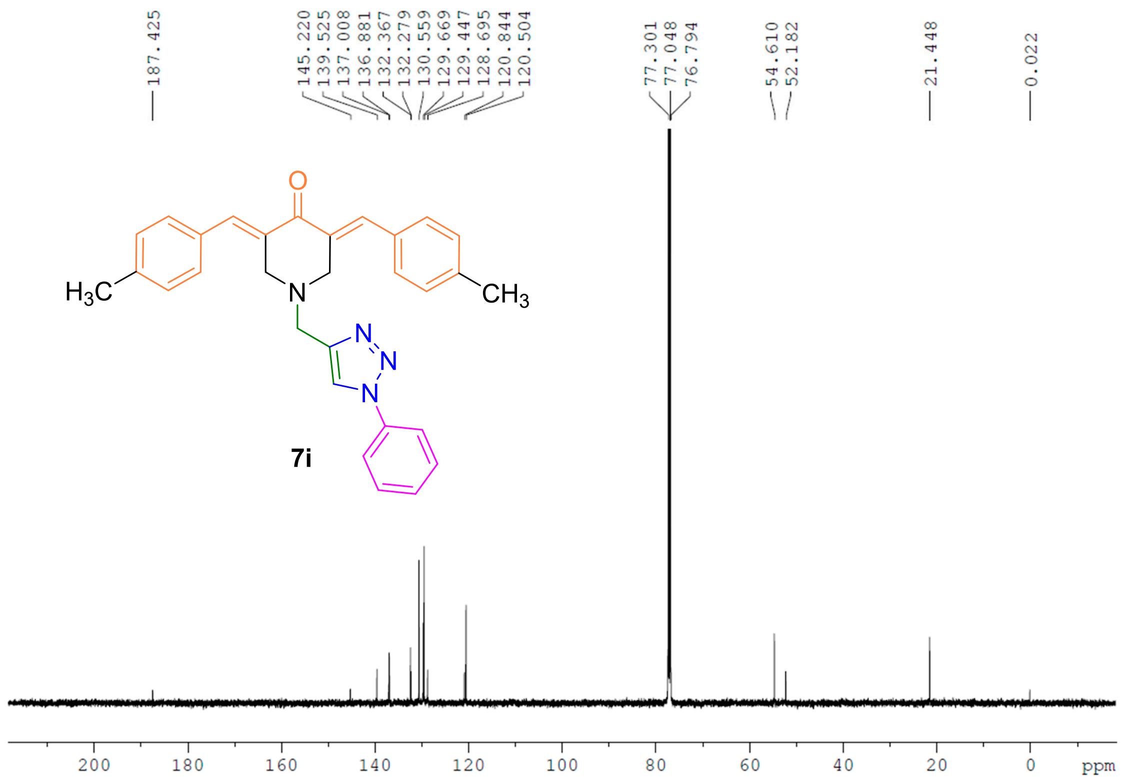

Figure S54. ^{13}C-NMR spectrum of Compound **7i**.

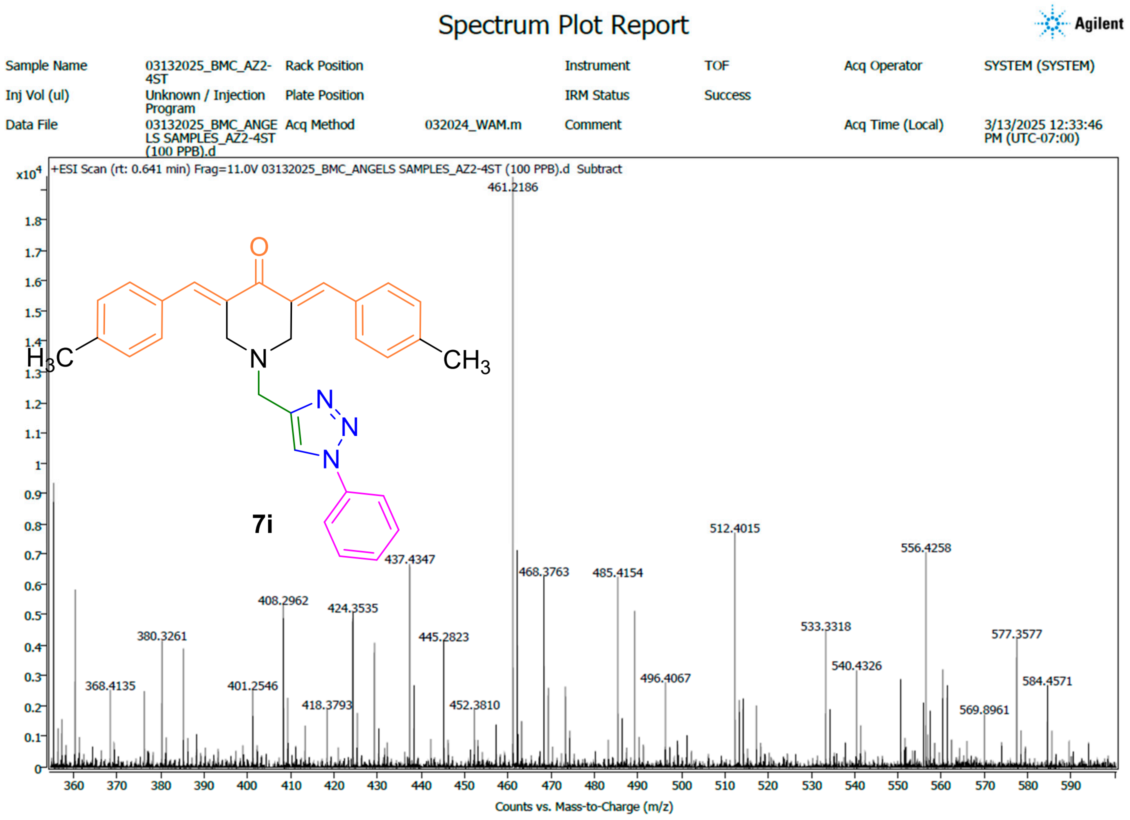

Figure S55. Mass spectrum of compound **7i**.

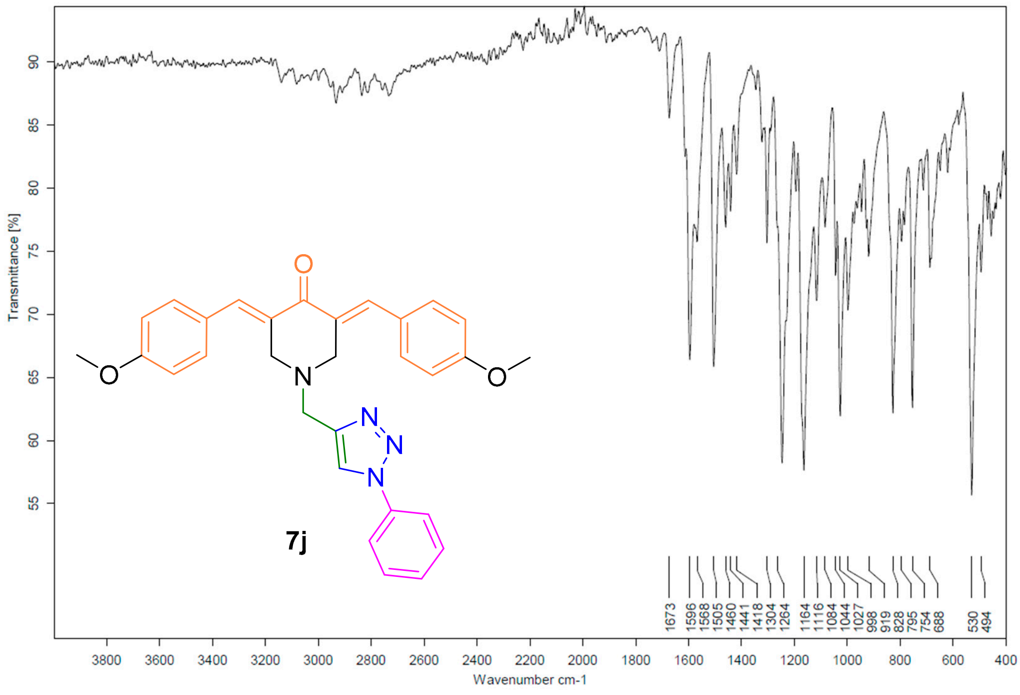

Figure S56. IR spectrum of compound **7j**.

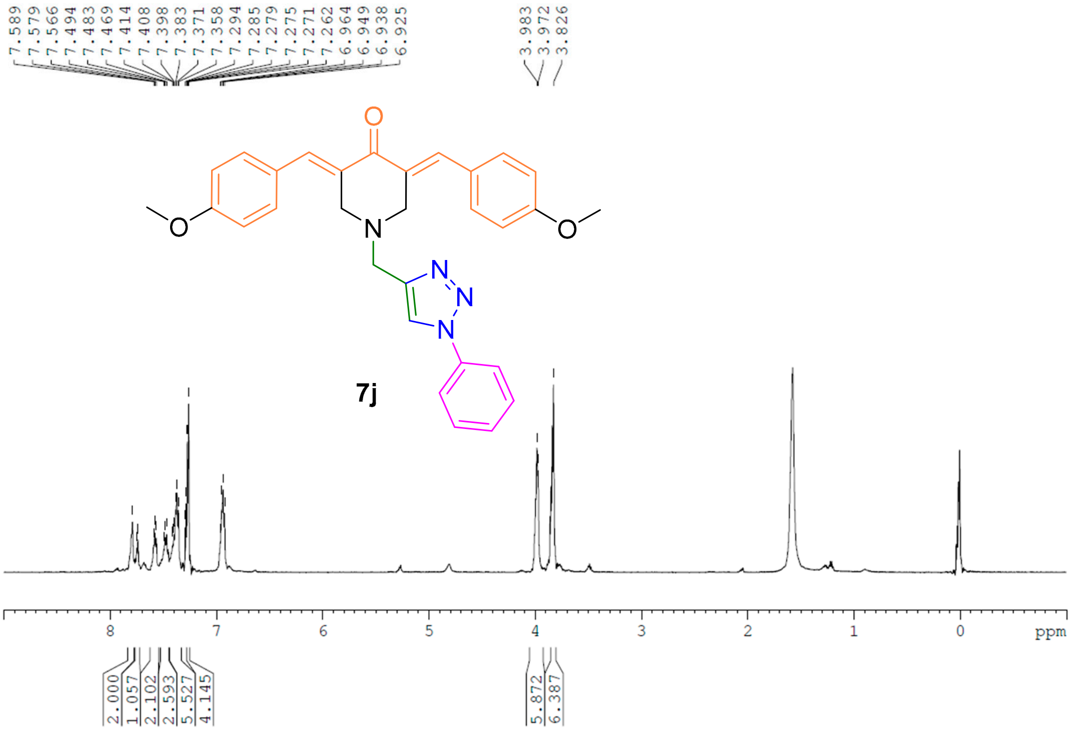

Figure S57. ^{1}H-NMR spectrum of compound **7j**.

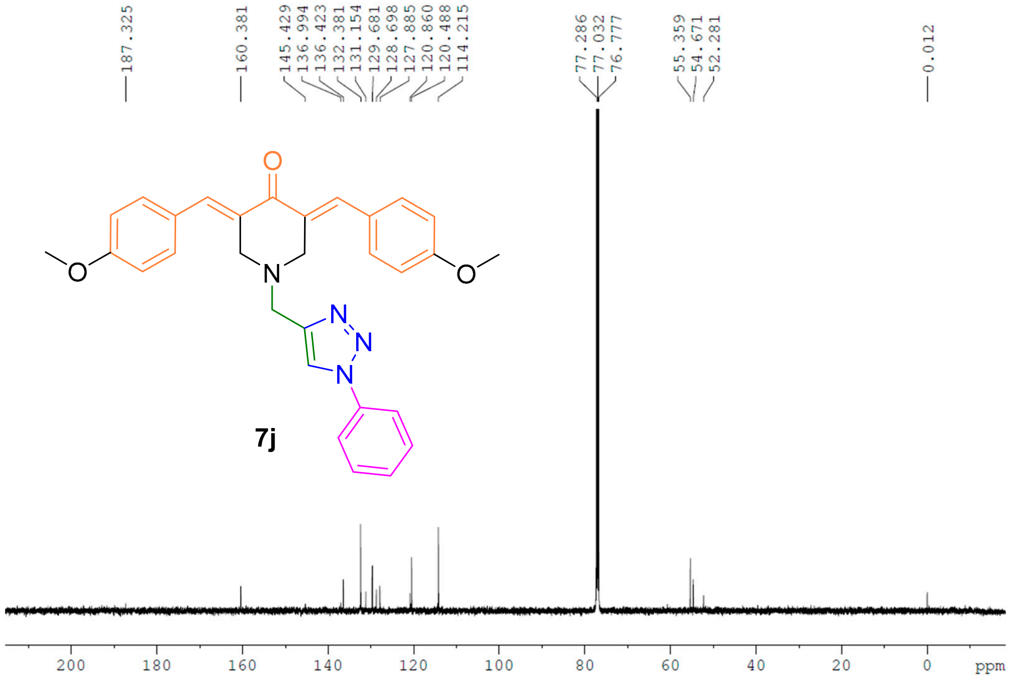

Figure S58. ^{13}C-NMR spectrum of compound **7j**.

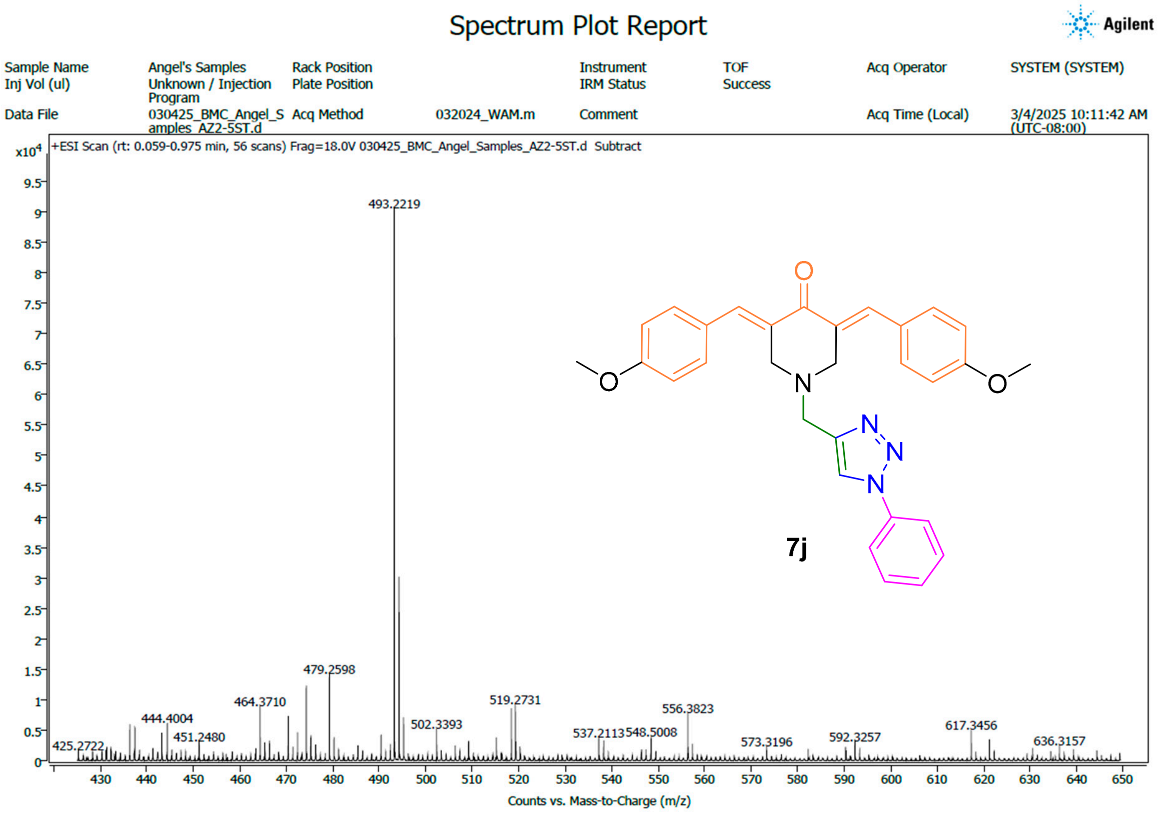

Figure S59. Mass spectrum of compound **7j**.

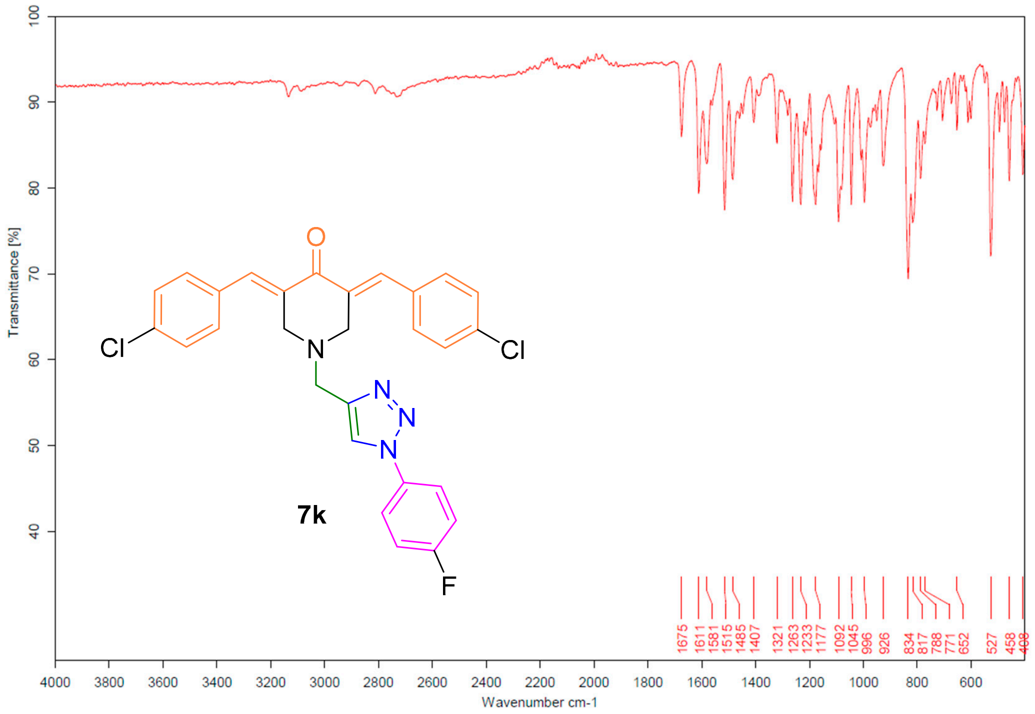

Figure S60. IR spectrum of compound **7k**.

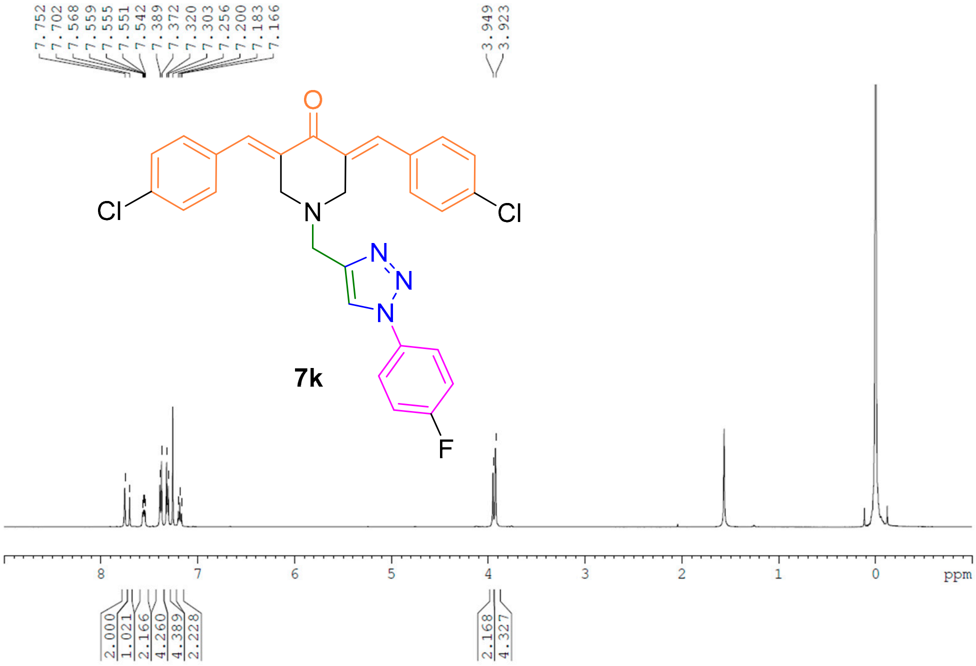

Figure S61. ^{1}H-NMR spectrum of compound **7k**.

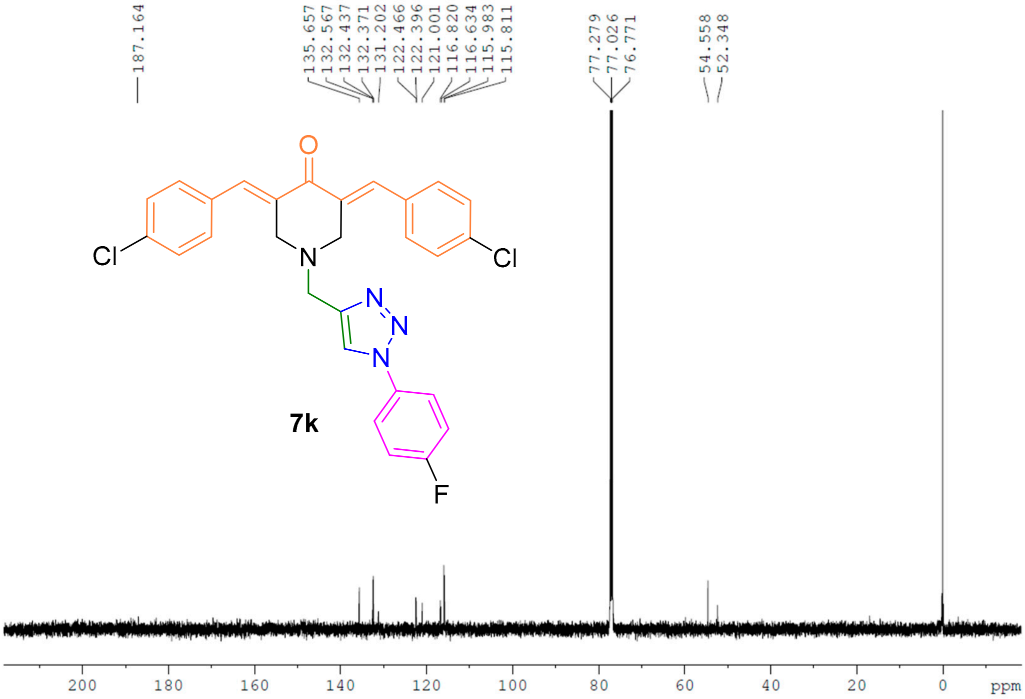

Figure S62. ^{13}C-NMR spectrum of compound **7k**.

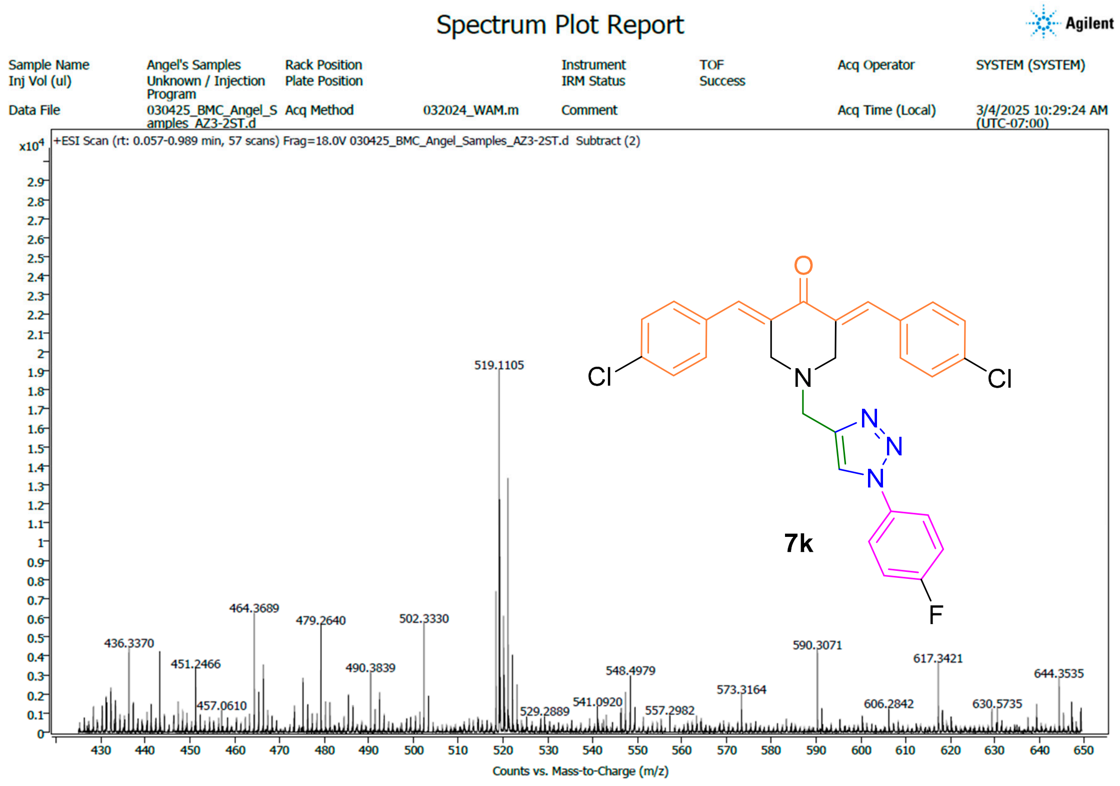

Figure S63. Mass spectrum of compound **7k**.

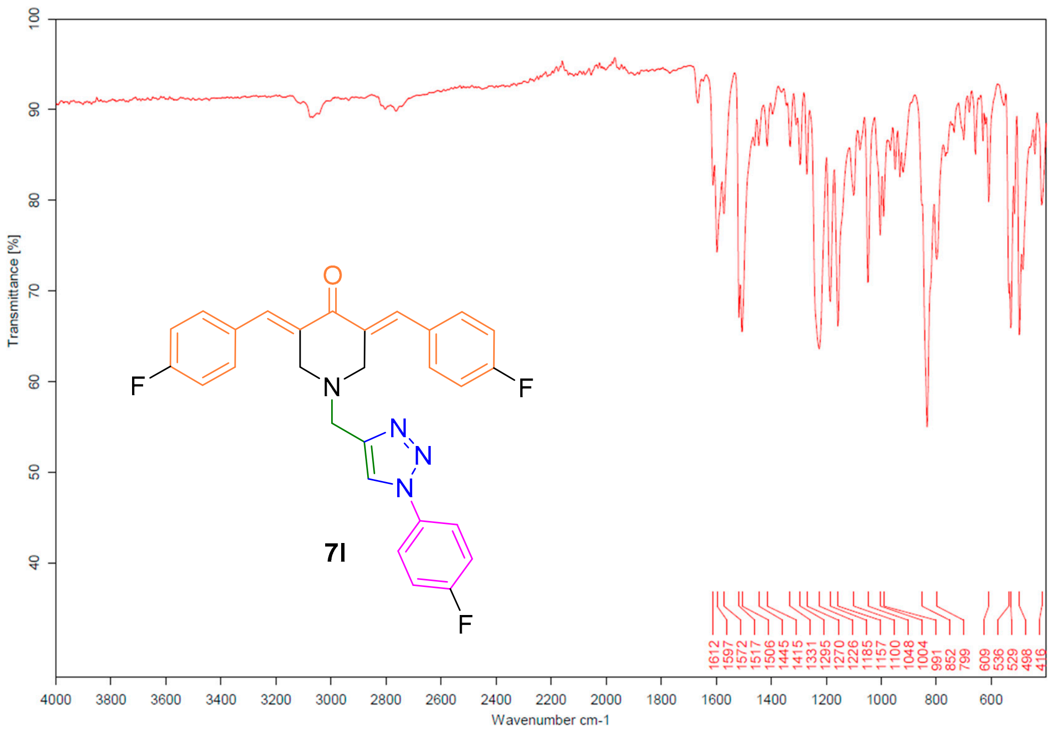

Figure S64. IR spectrum of compound **7l**.

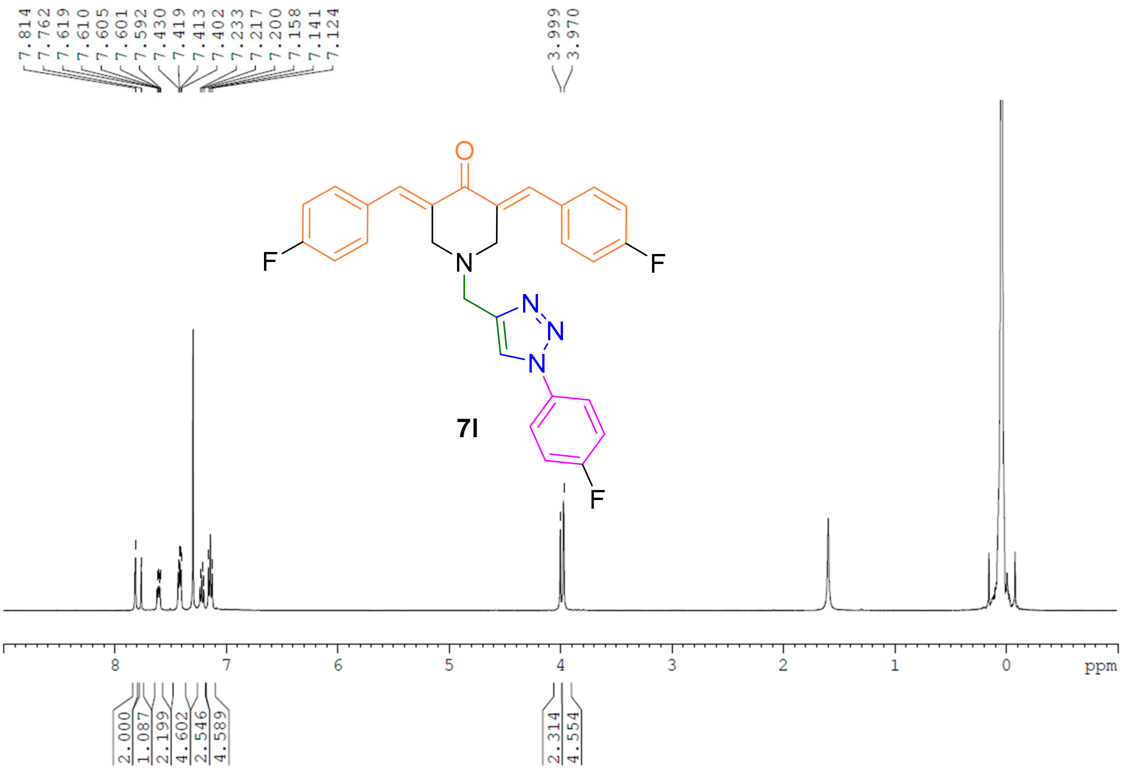

Figure S65. ^{1}H-NMR spectrum of compound **7l**.

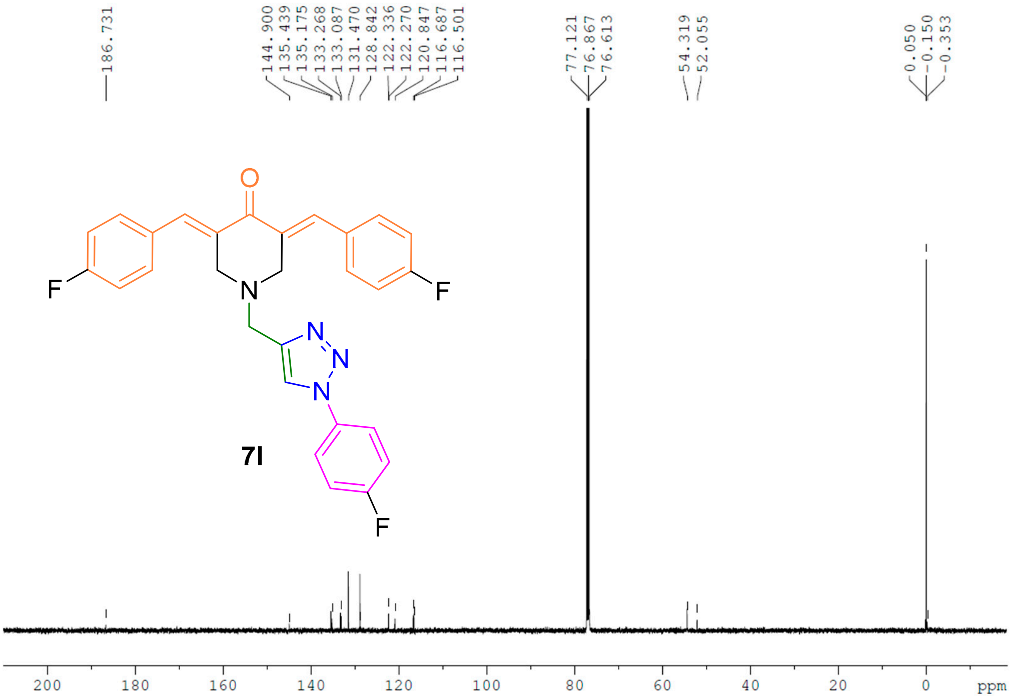

Figure S66. ^{13}C-NMR spectrum of compound **7l**.

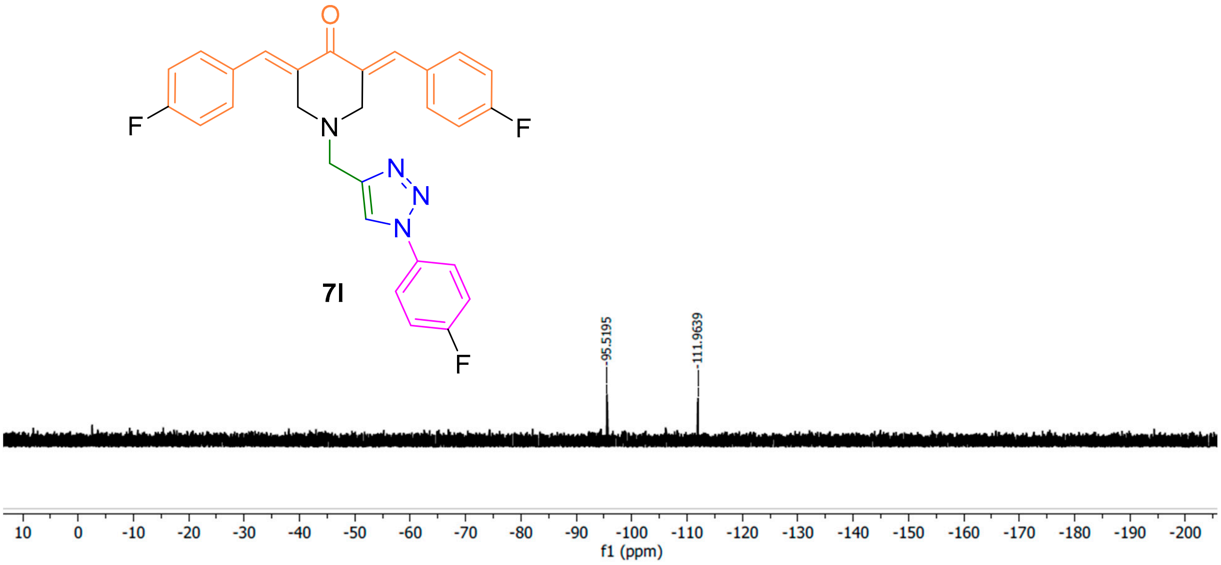

Figure S67. ^{19}F spectrum of compound **7l**.

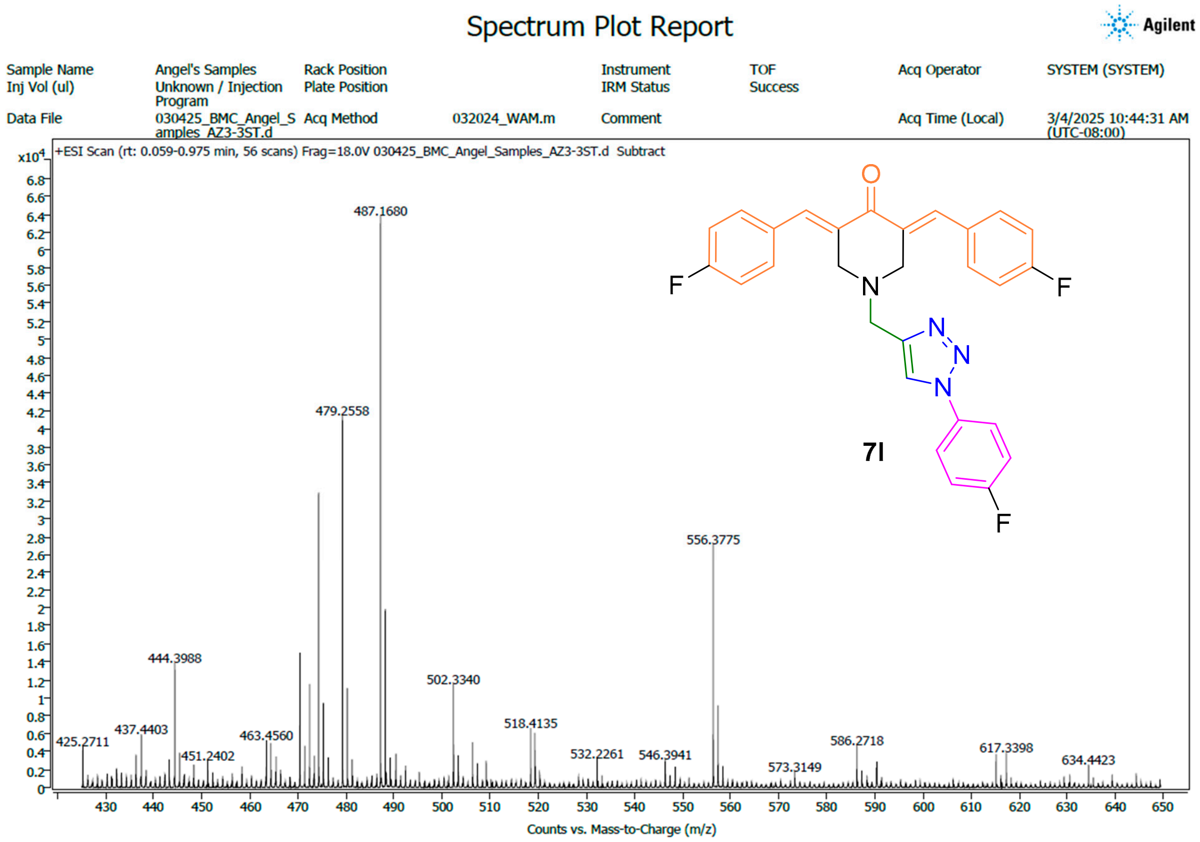

Figure S68. Mass spectrum of compound **7l**.

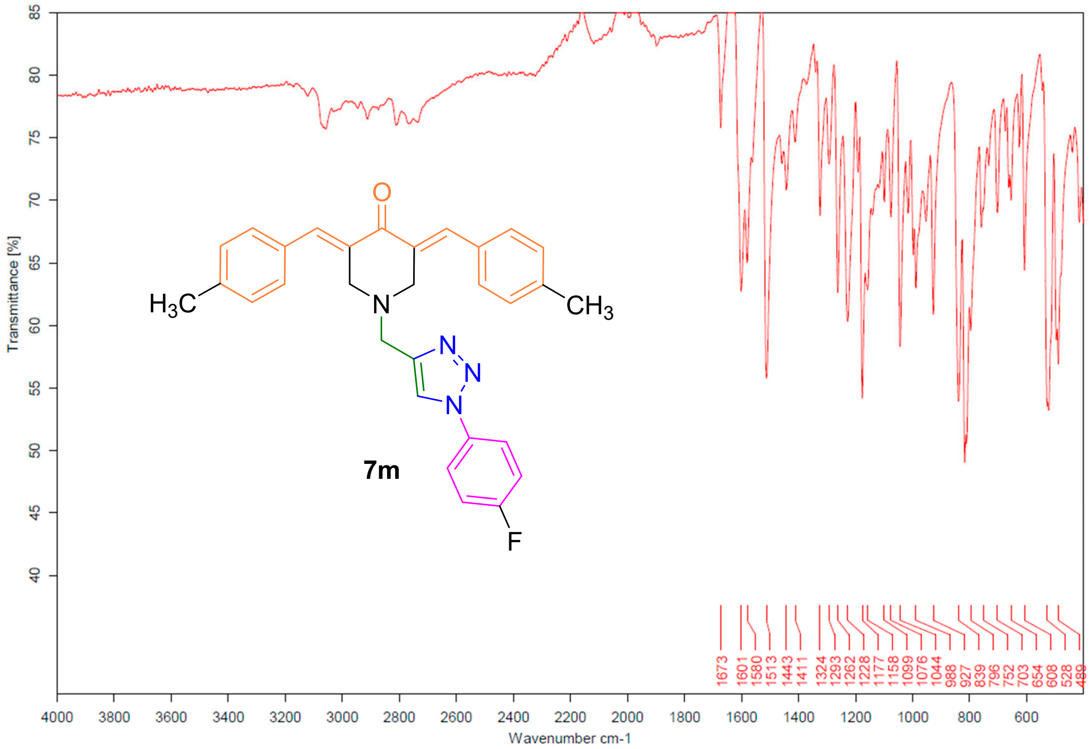

Figure S69. IR spectrum of compound **7m**.

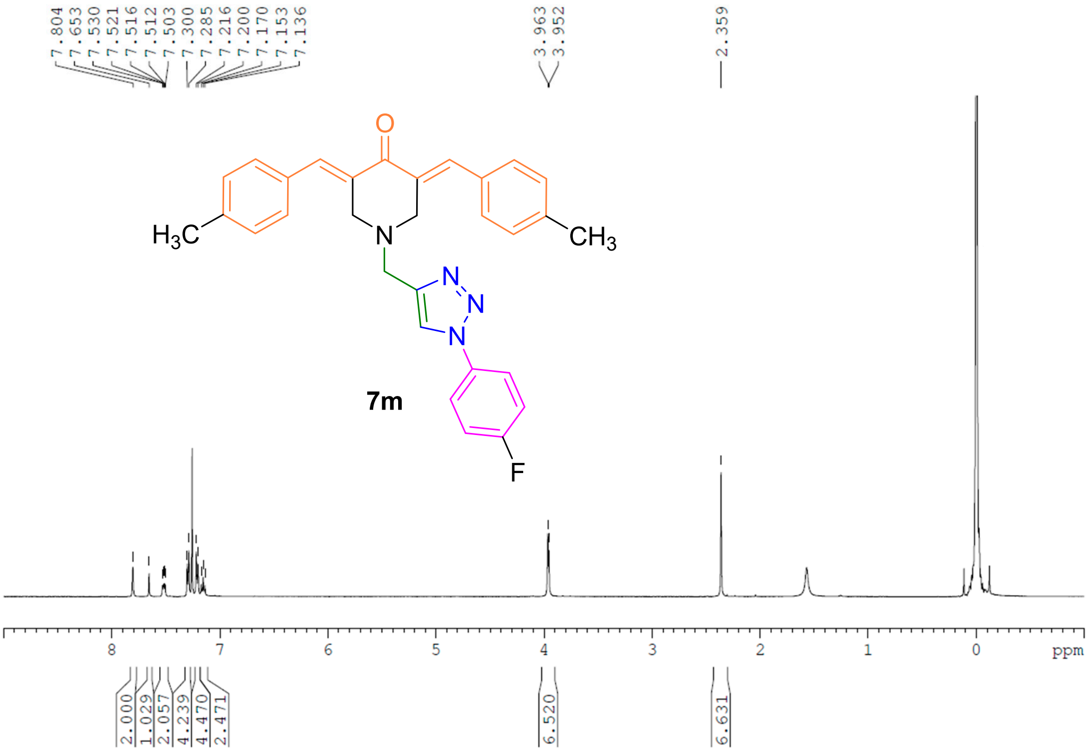

Figure S70. ^{1}H-NMR spectrum of Compound **7m**.

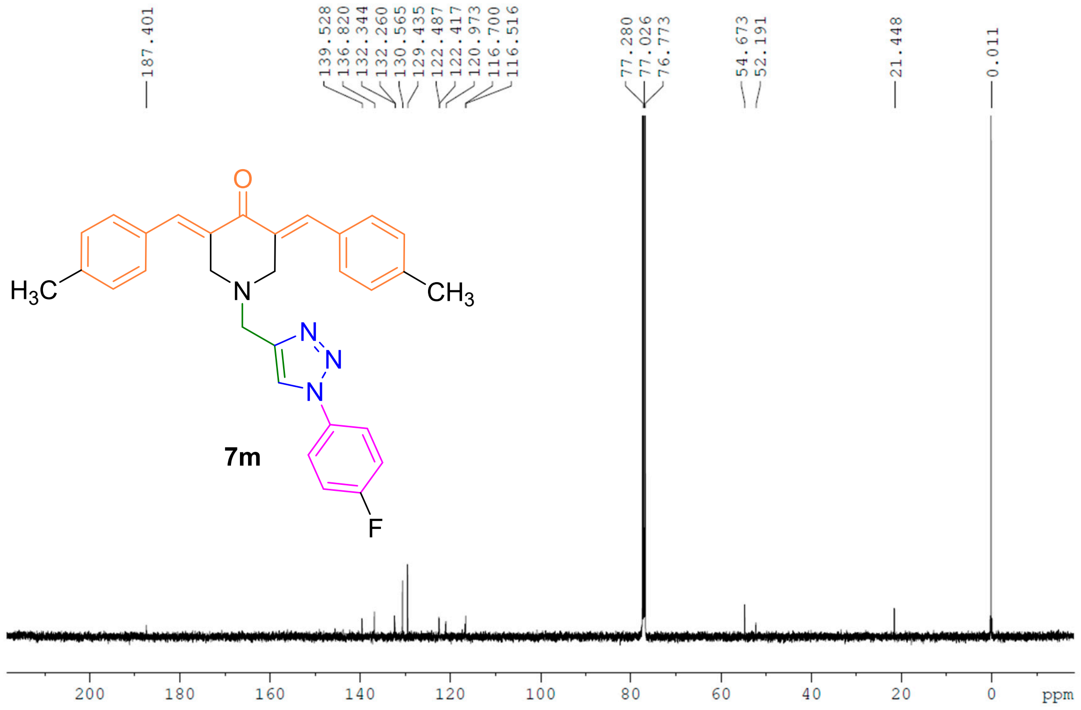

Figure S71. ^{13}C-NMR spectrum of compound **7m**.

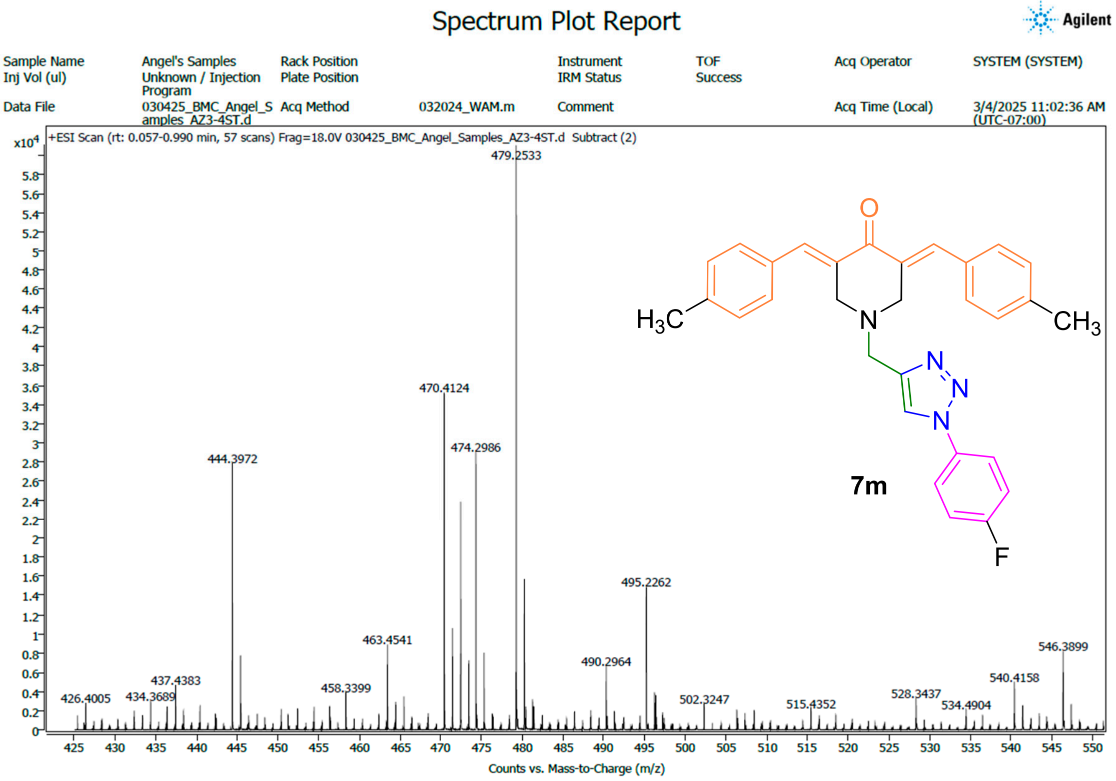

Figure S72. Mass spectrum of compound **7m**.

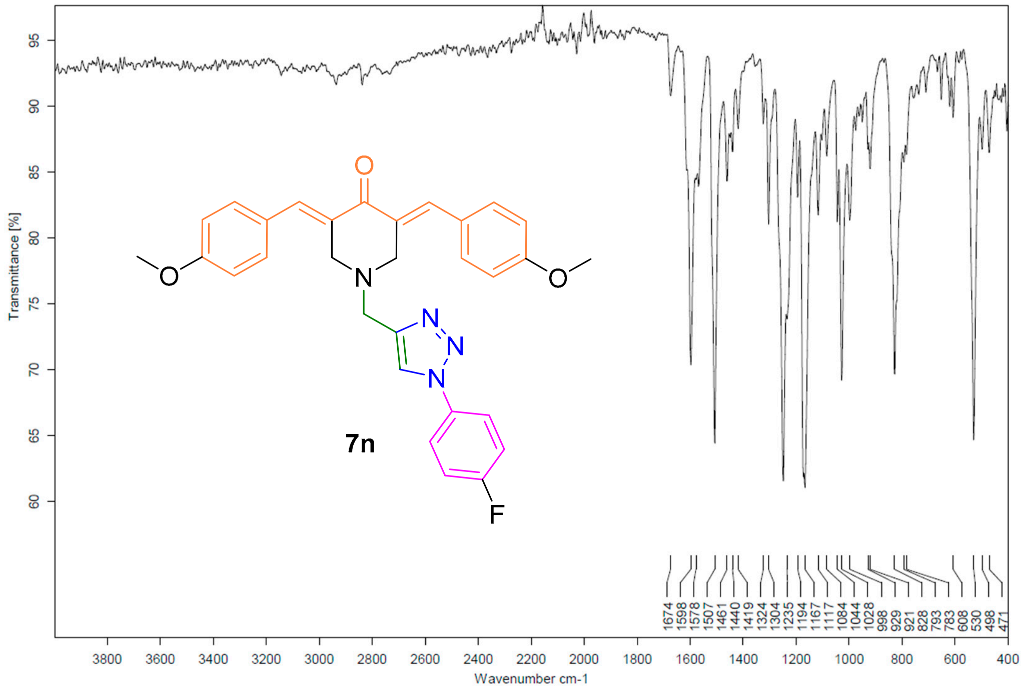

Figure S73. IR spectrum of compound **7n**.

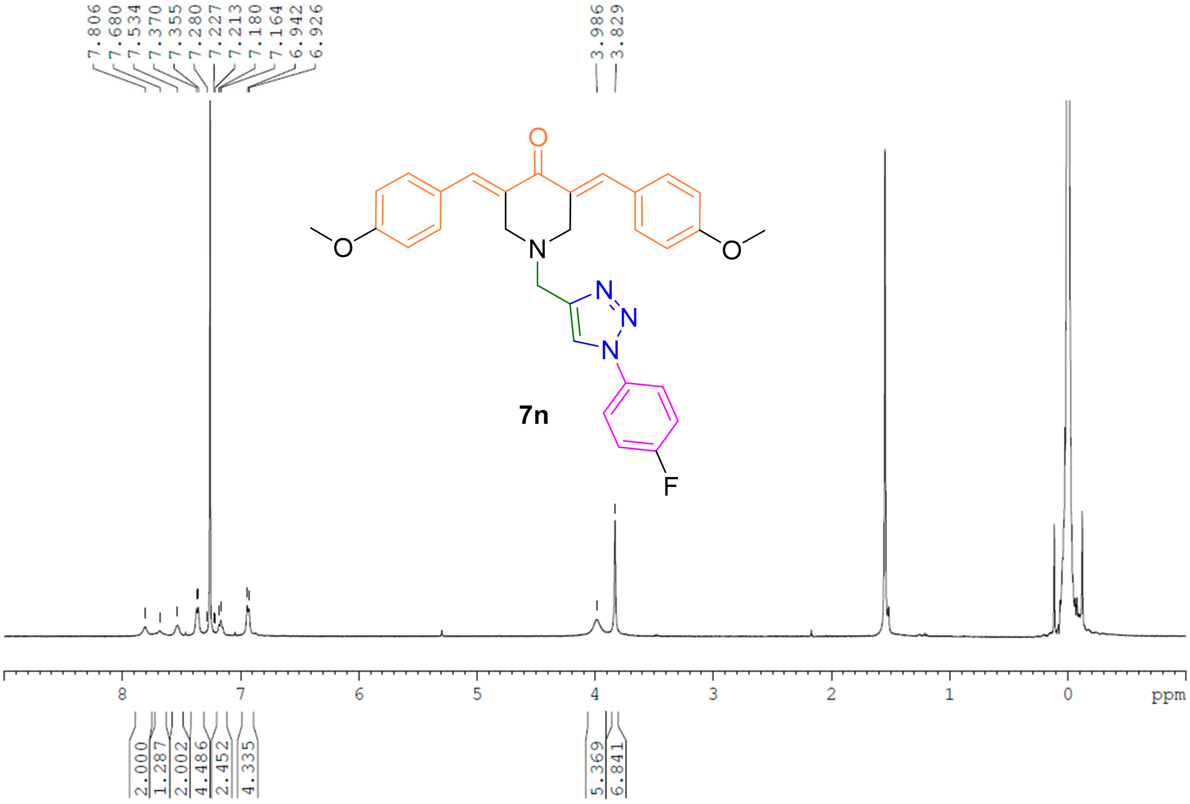

Figure S74. ^{1}H-NMR spectrum of compound **7n**.

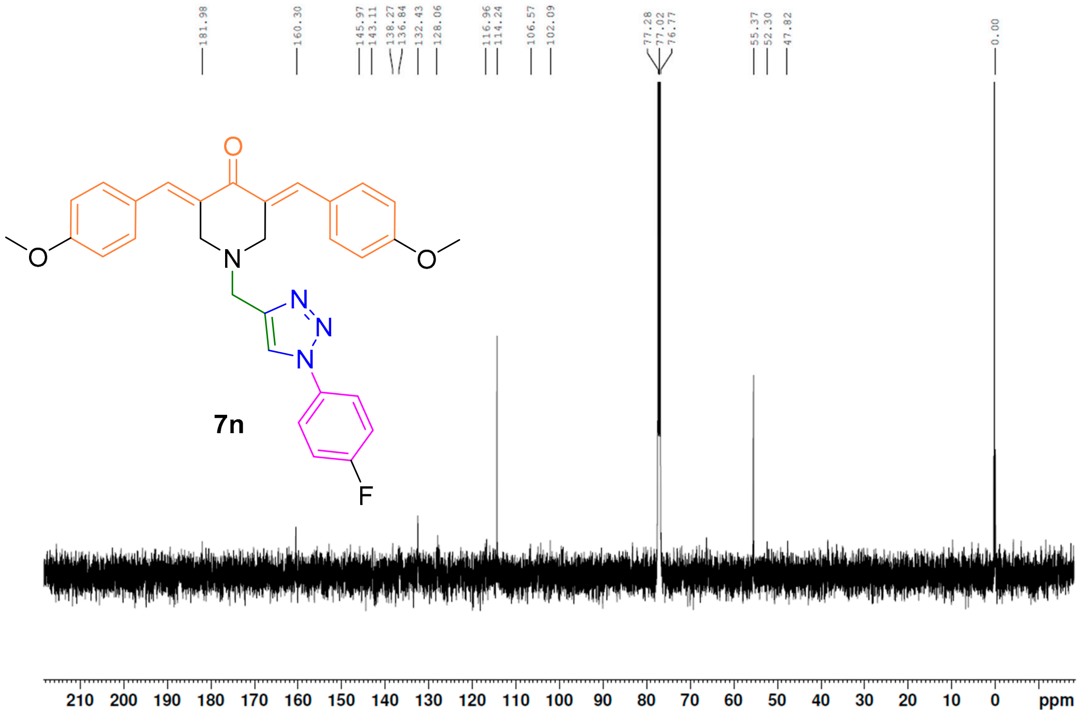

Figure S75. ^{13}C-NMR spectrum of compound **7n**.

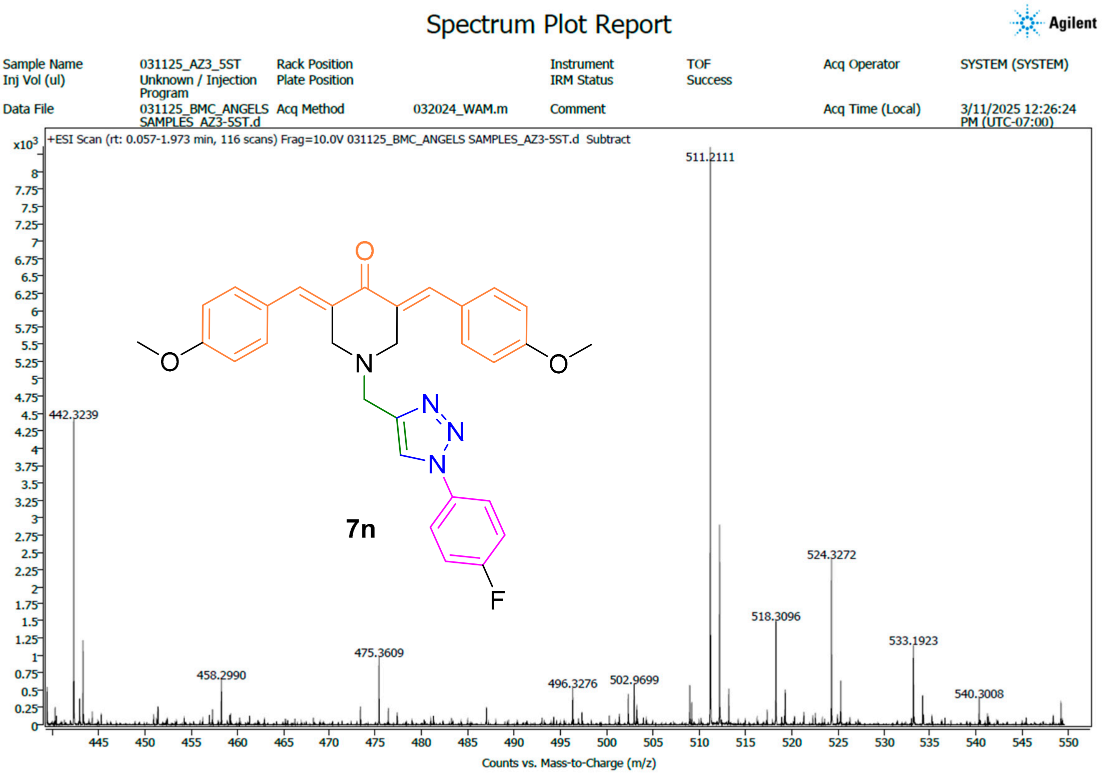

Figure S76. Mass spectrum of compound **7n**.

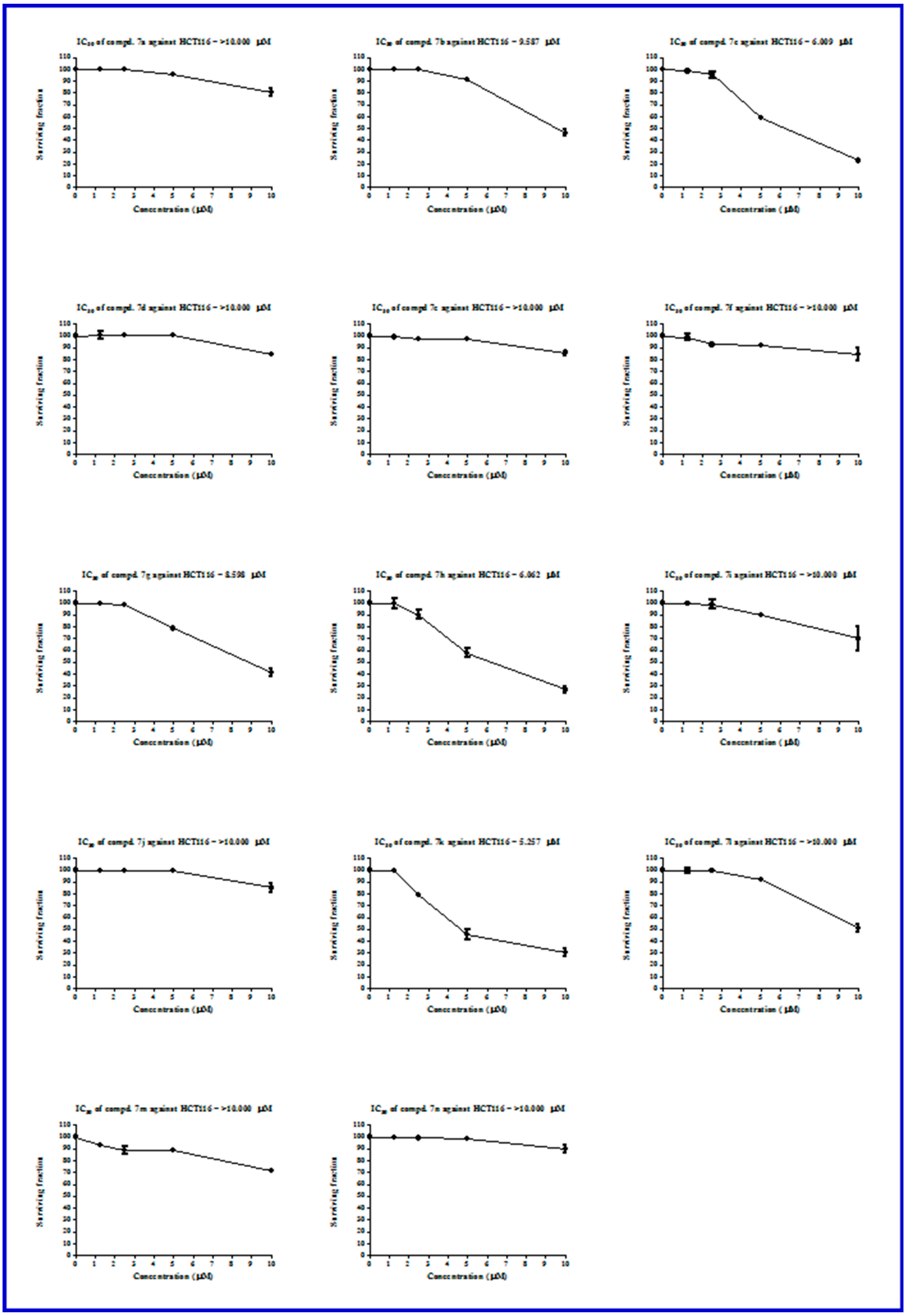

Figure S77. Dose-response curves of the synthesized compounds against the HCT116 cell line.

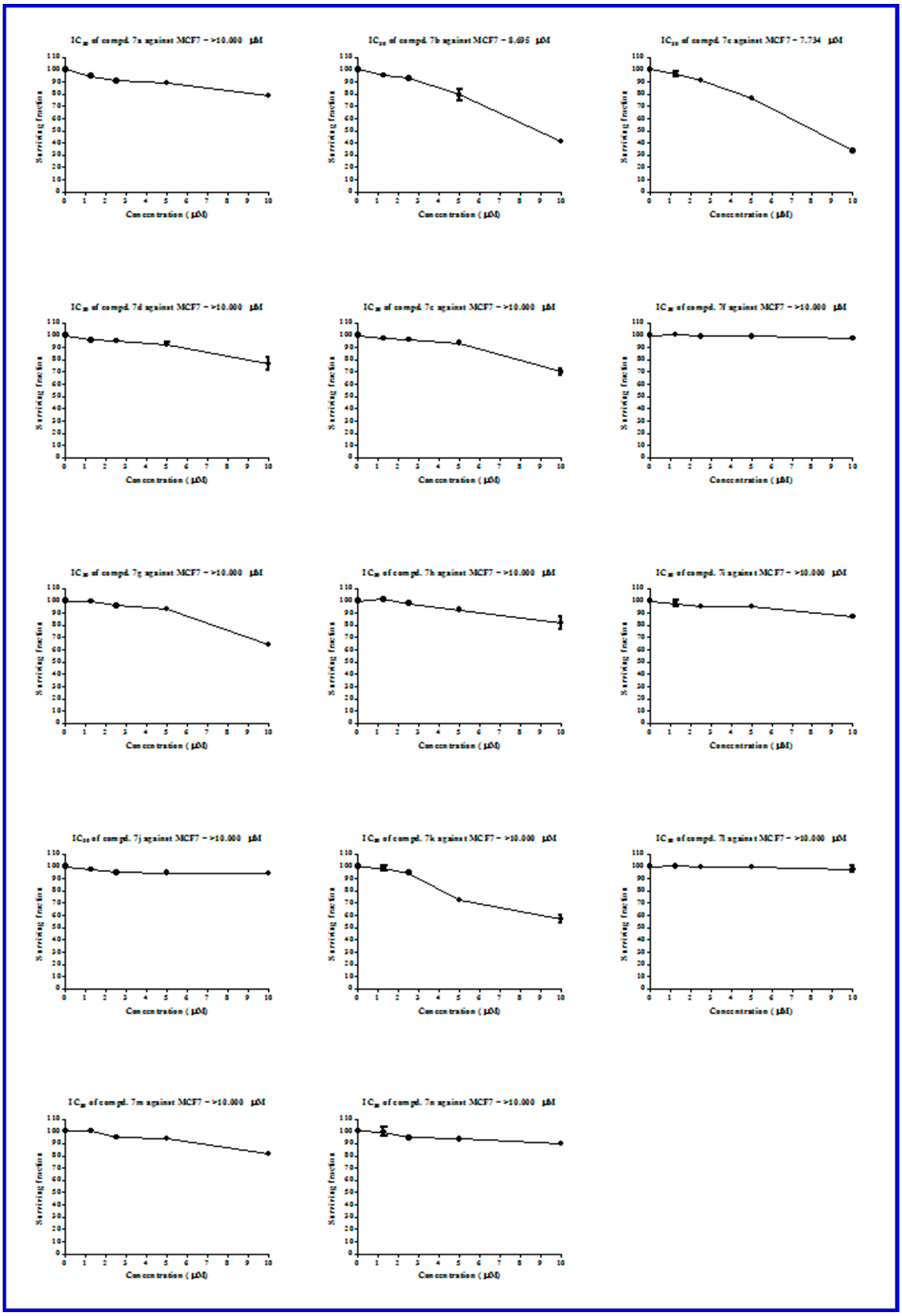

Figure S78. Dose response curves of the synthesized compounds against MCF7 cell line.

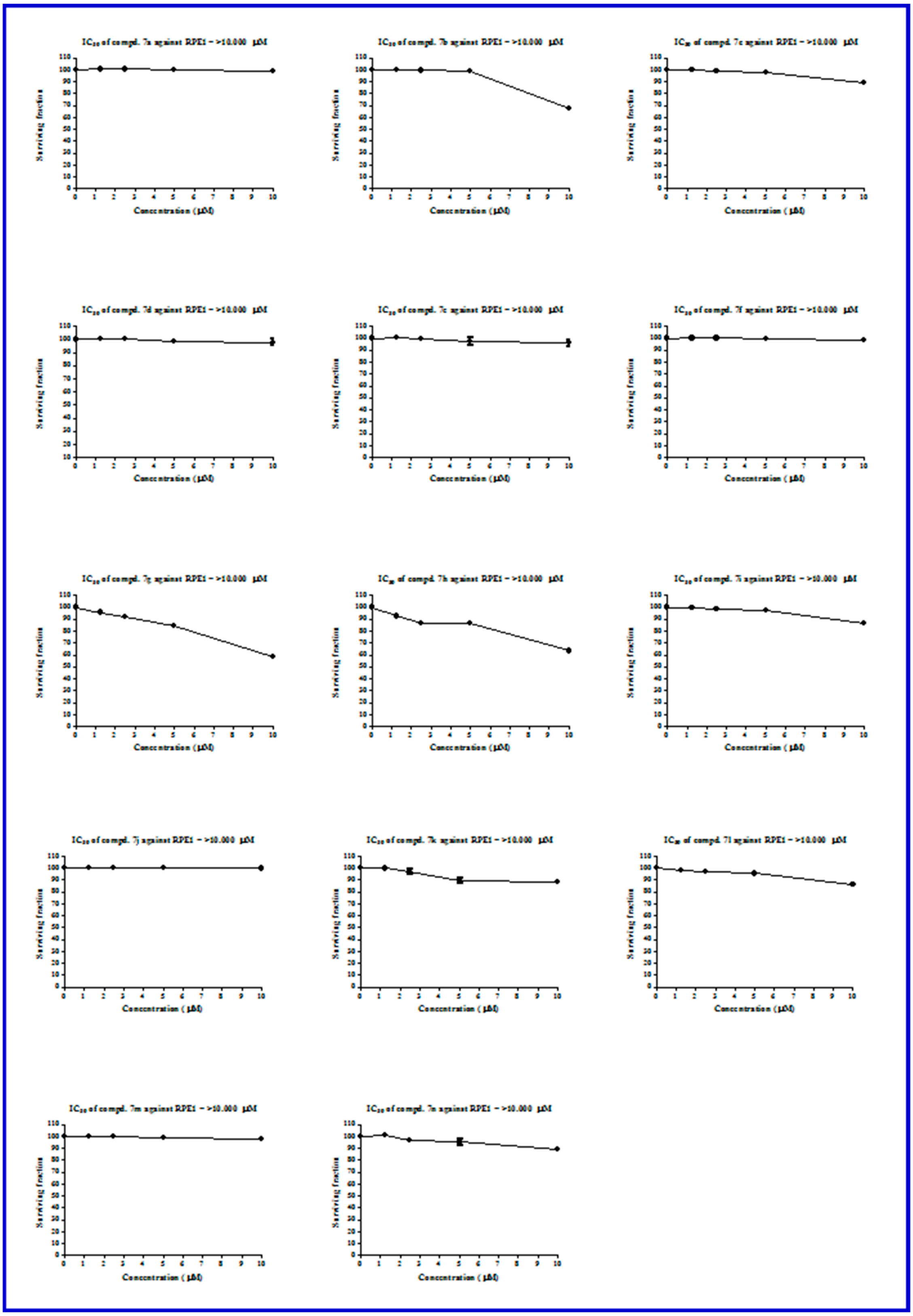

Figure S79. Dose response curves of the synthesized compounds against RPE1 cell line.

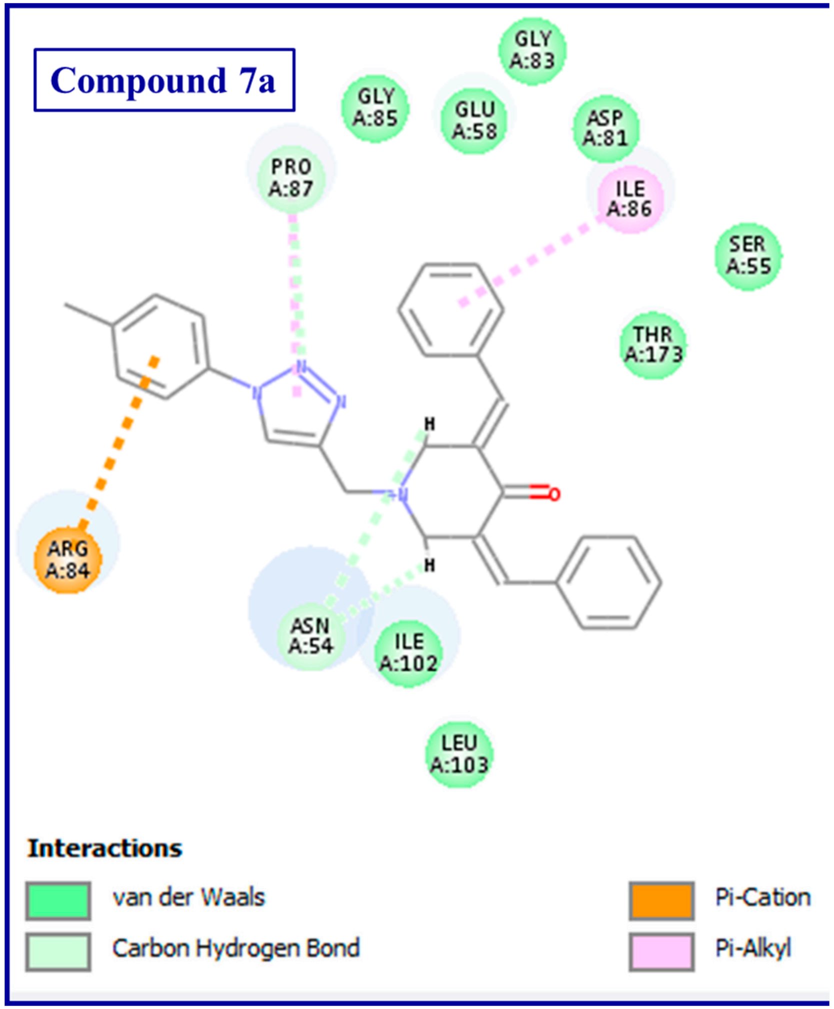

Compound 7a
GLY A:83
GLY A:85
GLU A:58
ASP A:81
PRO A:87
ILE A:86
SER A:55
THR A:173
ARG A:84
ASN A:54
ILE A:102
LEU A:103
Interactions
van der Waals
Carbon Hydrogen Bond
Pi-Cation
Pi-Alkyl

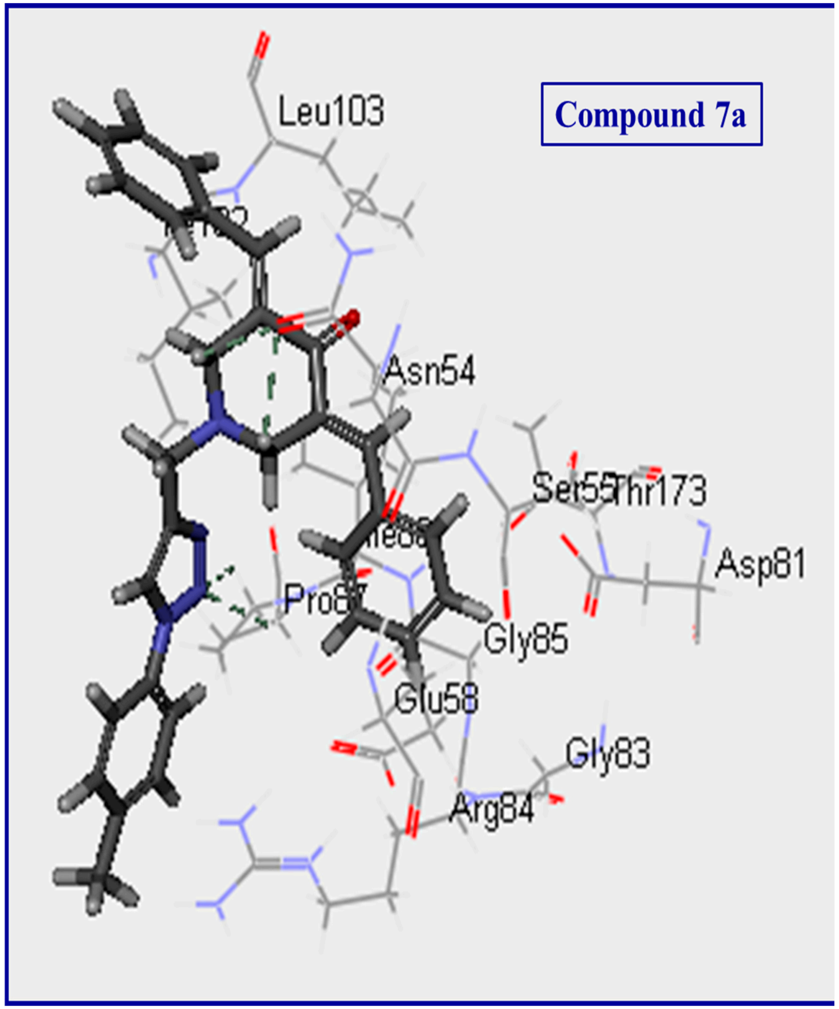

Compound 7a
Leu103
Asn54
Ser55
Thr173
Asp81
Pro87
Gly85
Glu58
Gly83
Arg84

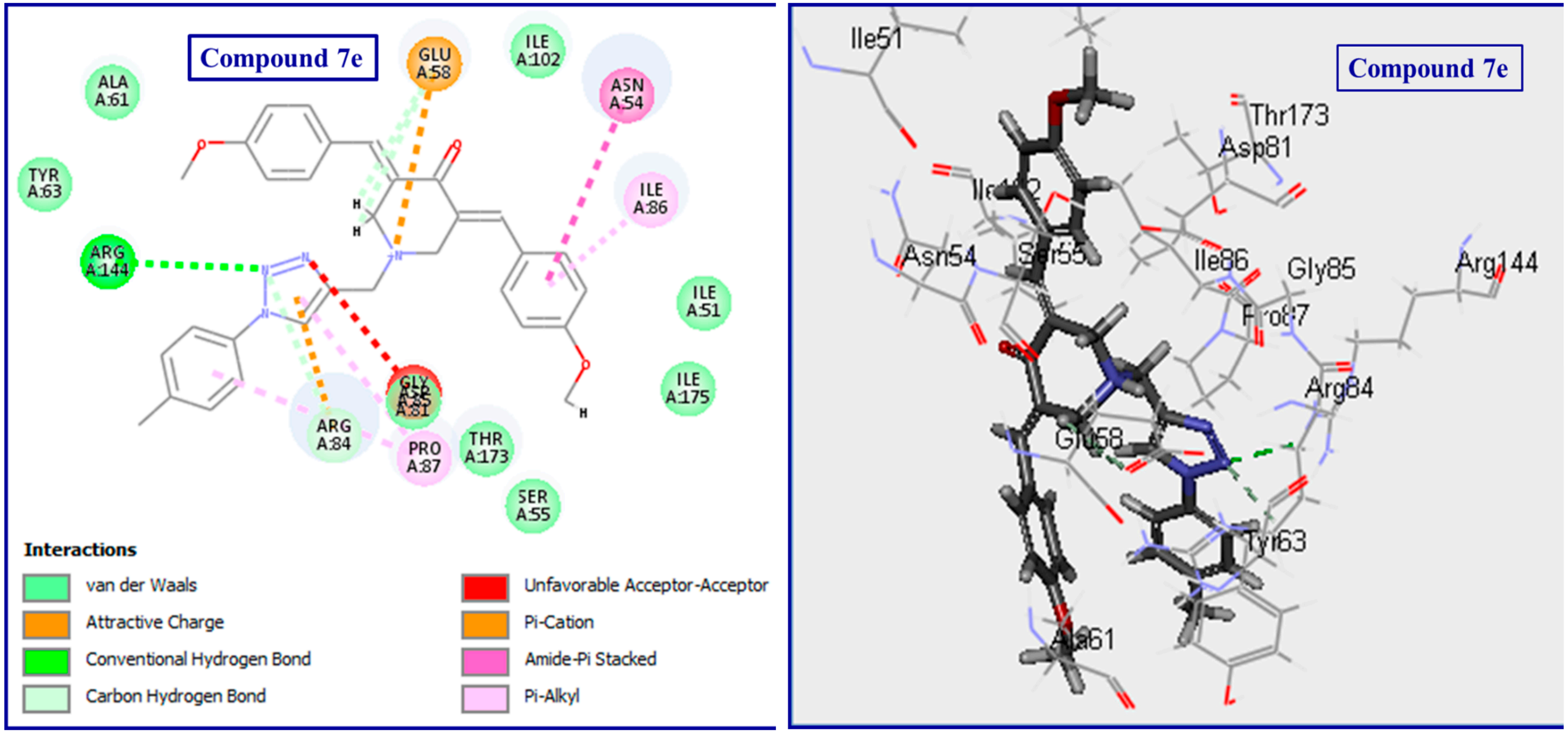

Compound 7e
GLU A:58
ILE A:102
ASN A:54
ALA A:61
TYR A:63
ILE A:86
ARG A:144
ILE A:51
ILE A:175
ARG A:84
PRO A:87
THR A:173
SER A:55
Interactions
van der Waals
Attractive Charge
Conventional Hydrogen Bond
Carbon Hydrogen Bond
Unfavorable Acceptor-Acceptor
Pi-Cation
Amide-Pi Stacked
Pi-Alkyl
Compound 7e
Ile51
Thr173
Asp81
Asn54
Ile86
Gly85
Arg144
Arg84
Glu58
Tyr63
Ala61

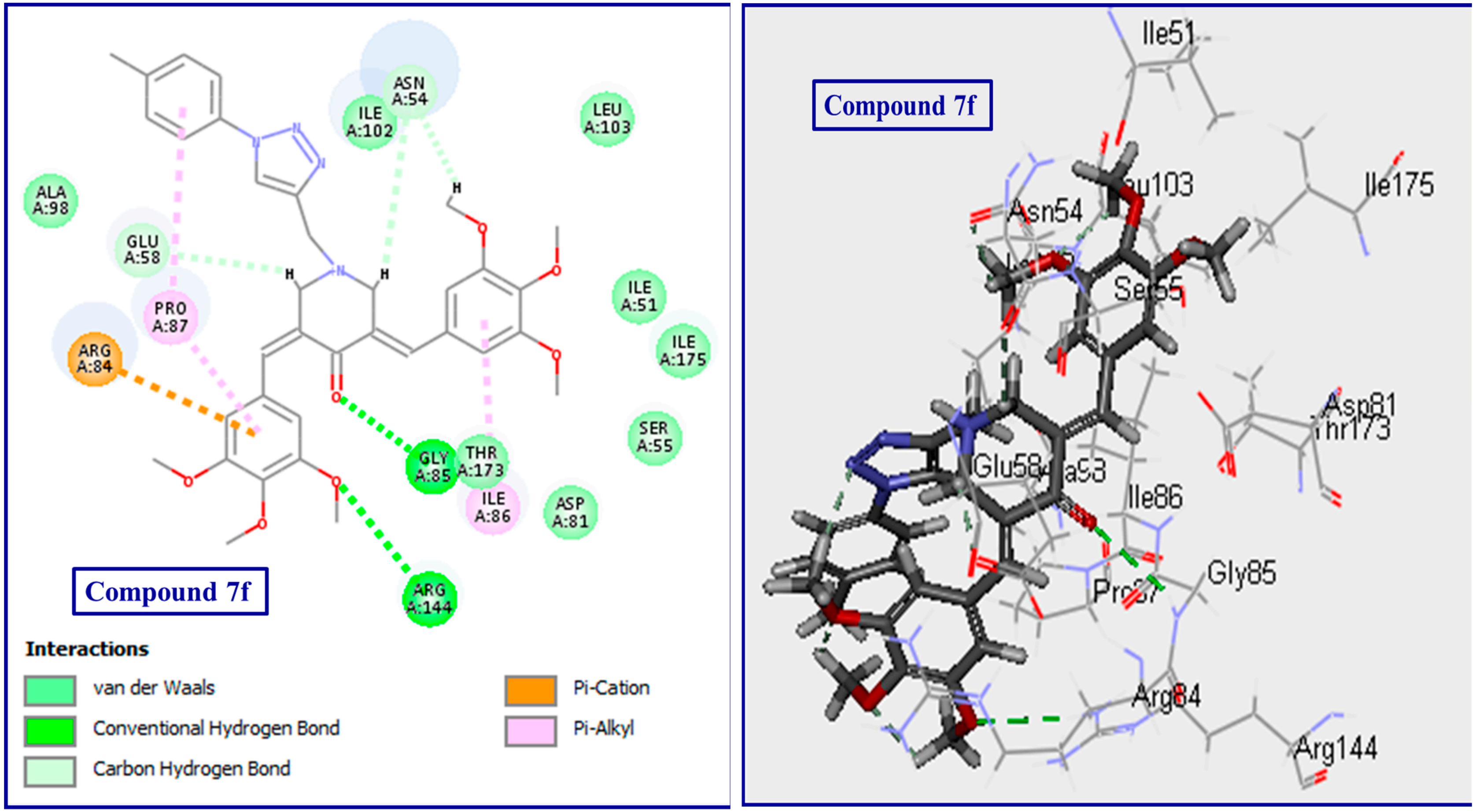

ASN A:54
ILE A:102
LEU A:103
ALA A:98
GLU A:58
ILE A:51
PRO A:87
ARG A:84
ILE A:175
SER A:55
GLY A:85
THR A:173
ILE A:86
ASP A:81
ARG A:144
Compound 7f
Interactions
van der Waals
Conventional Hydrogen Bond
Carbon Hydrogen Bond
Pi-Cation
Pi-Alkyl
Compound 7f
Ile51
Asn54
Ile175
Ser55
Asp81
Ile86
Gly85
Arg84
Arg144

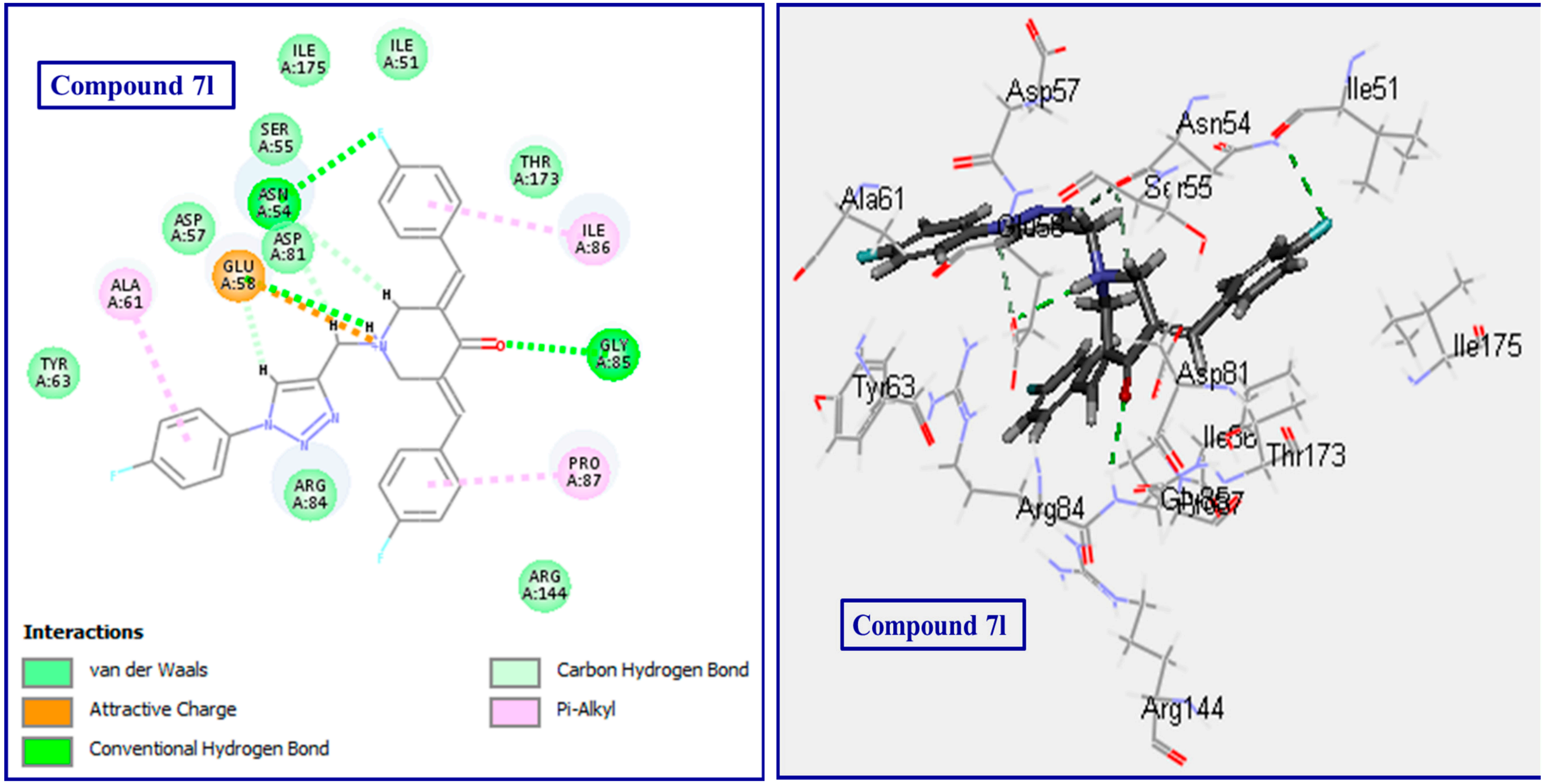
Compound 7l
ILE A:175
ILE A:51
SER A:55
ASN A:54
THR A:173
ASP A:57
ASP A:81
ILE A:86
GLU A:58
ALA A:61
GLY A:85
TYR A:63
PRO A:87
ARG A:84
ARG A:144
Interactions
van der Waals
Attractive Charge
Conventional Hydrogen Bond
Carbon Hydrogen Bond
Pi-Alkyl
Asp57
Ile51
Asn54
Ala61
Ser55
Ile175
Tyr63
Asp81
Ile86
Thr173
Arg84
Compound 7l
Arg144

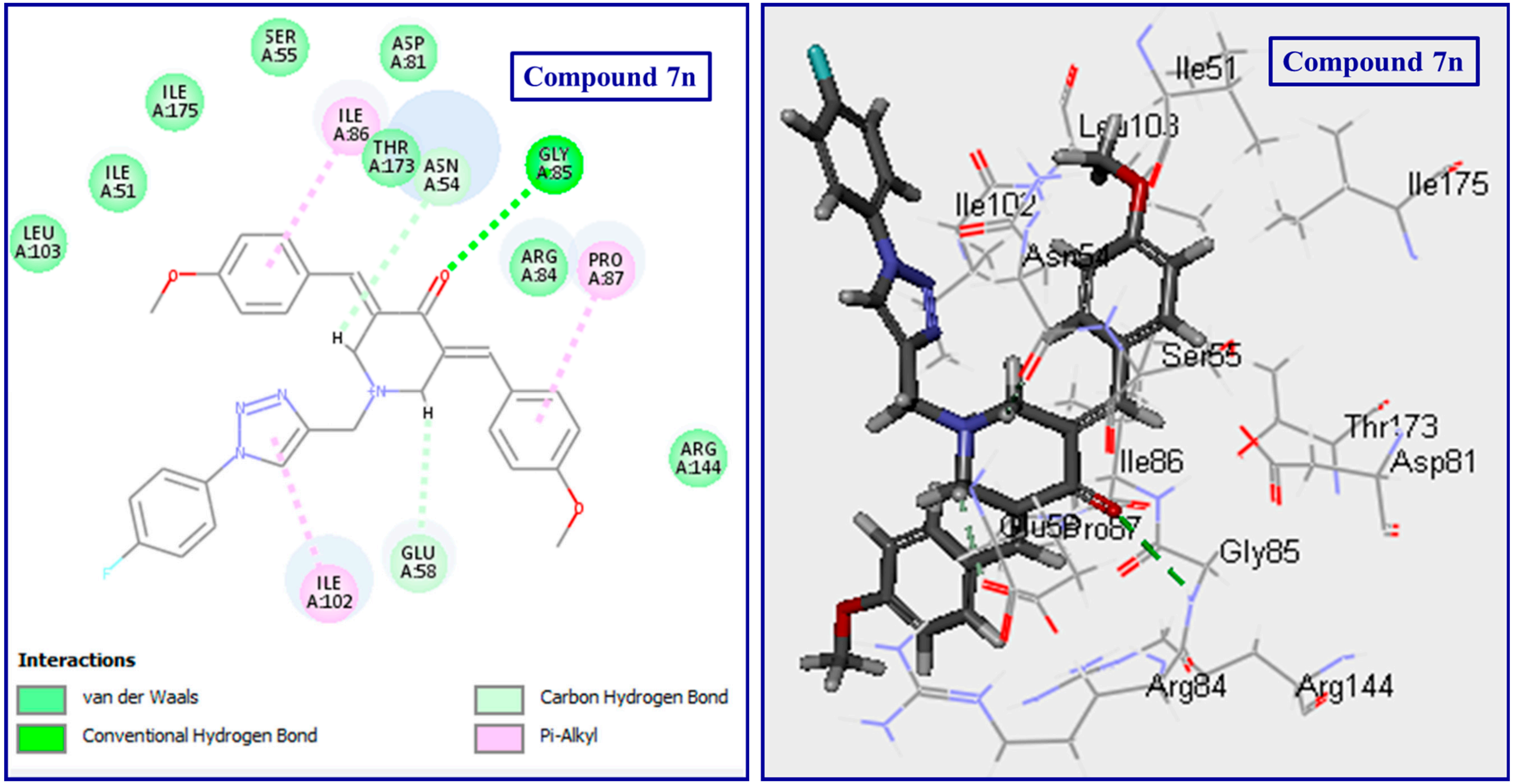
Compound 7n
SER A:55
ASP A:81
ILE A:175
ILE A:86
THR A:173
ASN A:54
GLY A:85
ILE A:51
LEU A:103
ARG A:84
PRO A:87
ARG A:144
GLU A:58
ILE A:102
Interactions
van der Waals
Conventional Hydrogen Bond
Carbon Hydrogen Bond
Pi-Alkyl
Compound 7n
Ile51
Ile102
Ile175
Ser55
Thr173
Asp81
Ile86
Gly85
Arg84
Arg144

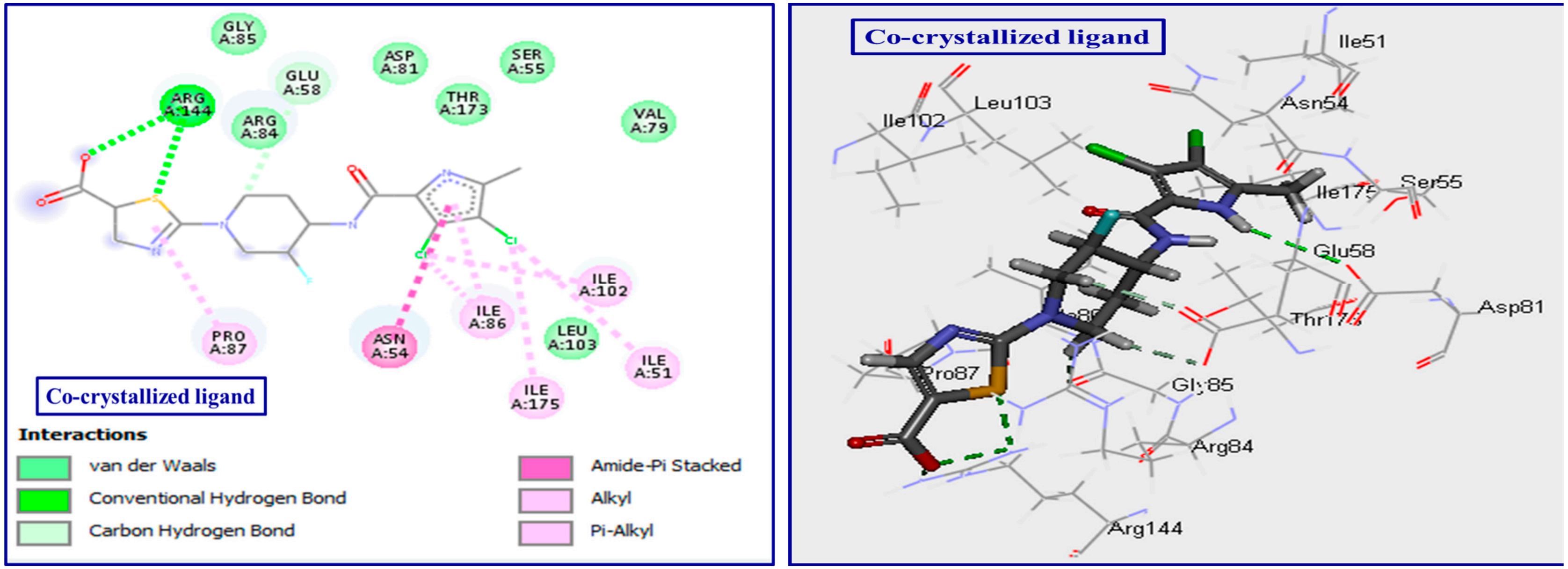

Figure S80. 2D-, and 3D-docking poses of the tested compounds and co-crystallized ligand in the active site of PDB ID: 3TTZ.

Table S1. Descriptors of the QSAR model for the synthesized compounds.

Entry	ID	Coefficient	*s*	*t*	Descriptor
1	0	-54.8747	5.466	-10.040	Intercept
2	D_1	0.168961	0.017	9.742	Min. e-n attraction for bond C-N
3	D_2	2.37733	0.340	6.997	Max. PI-PI bond order
4	D_3	-0.156992	0.044	-3.540	Average information content (order 1)

$N = 14$, $n = 3$, $R^2 = 0.931$, R^2cvOO = 0.830, R^2cvMO = 0.862, $F = 45.097$, $s^2 = 0.0002$

1/MIC = -54.8747 + (0.168961 x D_1) + (2.37733 x D_2) – (0.156992 x D_3)

Table S2. Observed and estimated MIC (μM) values of the synthesized compounds according to the BMLR-QSAR model.

Compd.	Observed		Estimated		Difference[#]
	MIC	1/MIC	MIC	1/MIC	
**7a**	17.9	0.055866	22.1	0.045206	-4.2
**7b**	496.7	0.002013	-66.8	-0.01497	563.5
**7c**	530.5	0.001885	81.8	0.012226	448.7
**7d**	269.7	0.003708	185.4	0.005393	84.3
**7e**	31.6	0.031646	30.8	0.03243	0.8
**7f**	12.8	0.078125	15.6	0.063997	-2.8
**7g**	127.6	0.007837	343.6	0.00291	-216.0
**7h**	273.2	0.00366	83.4	0.011985	189.8
**7i**	139	0.007194	36.8	0.027193	102.2
**7j**	259.9	0.003848	83.3	0.012011	176.6
**7k**	985.8	0.001014	195.9	0.005104	789.9
**7l**	8.2	0.121951	9.7	0.103288	-1.5
**7m**	267.5	0.003738	1458.8	0.000686	-1191.3
**7n**	7.8	0.128205	7.0	0.143233	0.8

[#]Difference between the observed and estimated MIC values.

Table S3. Molecular descriptor values of the QSAR model for the synthesized compounds.

Compd.	Descriptors#		
	D_1	D_2	D_3
**7a**	315.6359	0.88037	3.20566
**7b**	315.3881	0.89157	3.49177
**7c**	315.503	0.89484	3.49177
**7d**	315.5441	0.87702	3.30968
**7e**	315.4489	0.90672	3.4848
**7f**	315.3754	0.92248	3.44327
**7g**	315.2917	0.8956	3.33516
**7h**	315.3775	0.89331	3.33516
**7i**	315.4121	0.89366	3.28077
**7j**	315.2896	0.90241	3.37806
**7k**	315.669	0.88185	3.51907
**7l**	315.9376	0.89767	3.42241
**7m**	315.4711	0.88931	3.44716
**7n**	316.0709	0.91272	3.53933

#Descriptors; D_1 = Min. e-n attraction for bond C-N, D_2 = Max. PI-PI bond order, D_3 = Average information content (order 1).

Table S4. RMSD of the protein backbone (PDB ID: 3TTZ).

Entry	Item	RMSD (Å)
1	Conformation 1	0
2	Conformation 2	1.384
3	Conformation 3	1.851
4	Conformation 4	1.865
5	Conformation 5	1.913
6	Conformation 6	2.157
7	Conformation 7	2.126
8	Conformation 8	2.165
9	Conformation 9	2.332
10	Conformation 10	2.326
11	Conformation 11	2.258
12	Conformation 12	2.408
13	Conformation 13	2.428
14	Conformation 14	2.559
15	Conformation 15	2.521
16	Conformation 16	2.507
17	Conformation 17	2.664
18	Conformation 18	2.579
19	Conformation 19	2.512
20	Conformation 20	2.536
21	Conformation 21	2.534
22	Conformation 22	2.409
23	Conformation 23	2.511
24	Conformation 24	2.718
25	Conformation 25	2.706
26	Conformation 26	2.615
27	Conformation 27	2.590
28	Conformation 28	2.478
29	Conformation 29	2.576

30	Conformation 30	2.632
31	Conformation 31	2.677
32	Conformation 32	2.494
33	Conformation 33	2.303
34	Conformation 34	2.449
35	Conformation 35	2.354
36	Conformation 36	2.305
37	Conformation 37	2.356
38	Conformation 38	2.408
39	Conformation 39	2.540
40	Conformation 40	2.434
41	Conformation 41	2.273
42	Conformation 42	2.483
43	Conformation 43	2.777
44	Conformation 44	2.823
45	Conformation 45	2.783
46	Conformation 46	2.756
47	Conformation 47	2.903
48	Conformation 48	2.878
49	Conformation 49	2.682
50	Conformation 50	2.522
51	Conformation 51	2.521
52	Conformation 52	2.595
53	Conformation 53	2.524
54	Conformation 54	2.497
55	Conformation 55	2.594
56	Conformation 56	2.736
57	Conformation 57	2.815
58	Conformation 58	2.717
59	Conformation 59	2.589
60	Conformation 60	2.579

61	Conformation 61	2.526
62	Conformation 62	2.621
63	Conformation 63	2.691
64	Conformation 64	2.639
65	Conformation 65	2.612
66	Conformation 66	2.756
67	Conformation 67	2.786
68	Conformation 68	2.847
69	Conformation 69	2.950
70	Conformation 70	3.018
71	Conformation 71	3.223
72	Conformation 72	3.084
73	Conformation 73	2.940
74	Conformation 74	3.001
75	Conformation 75	2.918
76	Conformation 76	3.030
77	Conformation 77	3.114
78	Conformation 78	3.218
79	Conformation 79	3.341
80	Conformation 80	3.435
81	Conformation 81	3.384
82	Conformation 82	3.410
83	Conformation 83	3.309
84	Conformation 84	3.331
85	Conformation 85	3.440
86	Conformation 86	3.347
87	Conformation 87	3.215
88	Conformation 88	3.330
89	Conformation 89	3.159
90	Conformation 90	3.096
91	Conformation 91	3.253

92	Conformation 92	3.015
93	Conformation 93	3.070
94	Conformation 94	3.313
95	Conformation 95	3.067
96	Conformation 96	3.284
97	Conformation 97	3.162
98	Conformation 98	3.277
99	Conformation 99	3.357
100	Conformation 100	3.368
101	Average RMSD	2.702

Table S5. RMSD of the best conformation pose of compound **7a** docked in the protein active site of PDB ID: 3TTZ.

Entry	Item	RMSD (Å)
1	Conformation 1	0
2	Conformation 2	0.620
3	Conformation 3	0.806
4	Conformation 4	0.699
5	Conformation 5	0.498
6	Conformation 6	0.798
7	Conformation 7	0.596
8	Conformation 8	0.894
9	Conformation 9	0.998
10	Conformation 10	0.997
11	Conformation 11	1.002
12	Conformation 12	1.011
13	Conformation 13	1.007
14	Conformation 14	1.013
15	Conformation 15	1.016
16	Conformation 16	1.016
17	Conformation 17	1.026
18	Conformation 18	1.132
19	Conformation 19	1.033
20	Conformation 20	1.141
21	Conformation 21	1.207
22	Conformation 22	1.147
23	Conformation 23	1.149
24	Conformation 24	1.065
25	Conformation 25	1.065
26	Conformation 26	1.069
27	Conformation 27	1.142
28	Conformation 28	1.137

29	Conformation 29	1.090
30	Conformation 30	1.105
31	Conformation 31	1.120
32	Conformation 32	1.139
33	Conformation 33	1.134
34	Conformation 34	1.150
35	Conformation 35	1.162
36	Conformation 36	1.190
37	Conformation 37	1.206
38	Conformation 38	1.211
39	Conformation 39	1.220
40	Conformation 40	1.231
41	Conformation 41	1.240
42	Conformation 42	1.259
43	Conformation 43	1.281
44	Conformation 44	1.290
45	Conformation 45	1.310
46	Conformation 46	1.349
47	Conformation 47	1.364
48	Conformation 48	1.384
49	Conformation 49	1.384
50	Conformation 50	1.418
51	Conformation 51	1.438
52	Conformation 52	1.467
53	Conformation 53	1.489
54	Conformation 54	1.505
55	Conformation 55	1.548
56	Conformation 56	1.585
57	Conformation 57	1.599
58	Conformation 58	1.651
59	Conformation 59	1.581

60	Conformation 60	1.492
61	Conformation 61	1.434
62	Conformation 62	1.347
63	Conformation 63	1.279
64	Conformation 64	1.415
65	Conformation 65	1.390
66	Conformation 66	1.482
67	Conformation 67	1.721
68	Conformation 68	1.666
69	Conformation 69	1.706
70	Conformation 70	1.644
71	Conformation 71	1.584
72	Conformation 72	1.417
73	Conformation 73	1.361
74	Conformation 74	1.309
75	Conformation 75	1.350
76	Conformation 76	1.306
77	Conformation 77	1.350
78	Conformation 78	1.398
79	Conformation 79	1.447
80	Conformation 80	1.501
81	Conformation 81	1.541
82	Conformation 82	1.589
83	Conformation 83	1.444
84	Conformation 84	1.497
85	Conformation 85	1.449
86	Conformation 86	1.502
87	Conformation 87	1.457
88	Conformation 88	1.402
89	Conformation 89	1.364
90	Conformation 90	1.307

91	Conformation 91	1.182
92	Conformation 92	1.197
93	Conformation 93	1.183
94	Conformation 94	1.193
95	Conformation 95	1.251
96	Conformation 96	1.308
97	Conformation 97	1.312
98	Conformation 98	1.302
99	Conformation 99	1.312
100	Conformation 100	1.311
101	Average RMSD	1.251

Table S6. RMSD of the best conformation pose of compound **7l** docked in the protein active site of PDB ID: 3TTZ.

Entry	Item	RMSD (Å)
1	Conformation 1	0
2	Conformation 2	0.554
3	Conformation 3	0.559
4	Conformation 4	0.558
5	Conformation 5	0.494
6	Conformation 6	0.432
7	Conformation 7	0.486
8	Conformation 8	0.336
9	Conformation 9	0.374
10	Conformation 10	0.318
11	Conformation 11	0.409
12	Conformation 12	0.386
13	Conformation 13	0.359
14	Conformation 14	0.302
15	Conformation 15	0.250
16	Conformation 16	0.204
17	Conformation 17	0.254
18	Conformation 18	0.205
19	Conformation 19	0.232
20	Conformation 20	0.223
21	Conformation 21	0.287
22	Conformation 22	0.305
23	Conformation 23	0.312
24	Conformation 24	0.317
25	Conformation 25	0.323
26	Conformation 26	0.337
27	Conformation 27	0.338
28	Conformation 28	0.345

29	Conformation 29	0.389
30	Conformation 30	0.344
31	Conformation 31	0.381
32	Conformation 32	0.402
33	Conformation 33	0.414
34	Conformation 34	0.403
35	Conformation 35	0.418
36	Conformation 36	0.422
37	Conformation 37	0.420
38	Conformation 38	0.434
39	Conformation 39	0.443
40	Conformation 40	0.409
41	Conformation 41	0.425
42	Conformation 42	0.422
43	Conformation 43	0.405
44	Conformation 44	0.400
45	Conformation 45	0.431
46	Conformation 46	0.442
47	Conformation 47	0.458
48	Conformation 48	0.467
49	Conformation 49	0.465
50	Conformation 50	0.485
51	Conformation 51	0.469
52	Conformation 52	0.503
53	Conformation 53	0.547
54	Conformation 54	0.529
55	Conformation 55	0.545
56	Conformation 56	0.553
57	Conformation 57	0.569
58	Conformation 58	0.570
59	Conformation 59	0.573

60	Conformation 60	0.561
61	Conformation 61	0.548
62	Conformation 62	0.557
63	Conformation 63	0.570
64	Conformation 64	0.589
65	Conformation 65	0.594
66	Conformation 66	0.604
67	Conformation 67	0.603
68	Conformation 68	0.605
69	Conformation 69	0.611
70	Conformation 70	0.623
71	Conformation 71	0.615
72	Conformation 72	0.631
73	Conformation 73	0.643
74	Conformation 74	0.670
75	Conformation 75	0.659
76	Conformation 76	0.660
77	Conformation 77	0.650
78	Conformation 78	0.651
79	Conformation 79	0.674
80	Conformation 80	0.699
81	Conformation 81	0.705
82	Conformation 82	0.738
83	Conformation 83	0.764
84	Conformation 84	0.762
85	Conformation 85	0.805
86	Conformation 86	0.841
87	Conformation 87	0.843
88	Conformation 88	0.866
89	Conformation 89	0.877
90	Conformation 90	0.899

91	Conformation 91	0.927
92	Conformation 92	0.948
93	Conformation 93	0.965
94	Conformation 94	0.977
95	Conformation 95	0.984
96	Conformation 96	0.992
97	Conformation 97	0.893
98	Conformation 98	0.880
99	Conformation 99	0.870
100	Conformation 100	0.860
101	Average RMSD	0.540

Table S7. RMSF of the protein backbone of PDB ID: 3TTZ.

Item	RMSF
TYR14	3.23726
GLY15	3.34221
ALA16	2.59499
GLY17	2.31148
GLN18	2.47618
ILE19	2.03052
GLN20	2.17143
VAL21	1.61594
LEU22	1.56341
GLU23	1.67089
GLY24	1.10036
LEU25	1.16224
GLU26	1.23777
ALA27	0.951982
VAL28	0.787582
ARG29	0.744405
LYS30	1.46404
ARG31	1.66497
PRO32	0.943588
GLY33	1.18176
MET34	1.66104
TYR35	1.29296
ILE36	1.1432
GLY37	1.31563
SER38	1.46943
THR39	1.3179
SER40	0.996606
GLU41	1.24876
ARG42	1.45164

GLY43	0.736039
LEU44	0.70125
HIS45	0.833218
HIS46	1.29448
LEU47	0.784793
VAL48	0.819799
TRP49	1.36535
GLU50	1.02234
ILE51	0.863619
VAL52	1.06278
ASP53	1.31501
ASN54	1.27503
SER55	1.09495
ILE56	1.33712
ASP57	1.47014
GLU58	1.49179
ALA59	1.44763
LEU60	1.72794
ALA61	1.73312
GLY62	1.74652
TYR63	1.83289
ALA64	1.43433
ASN65	1.67915
GLN66	1.71561
ILE67	1.26515
GLU68	1.4194
VAL69	1.03616
VAL70	1.0098
ILE71	1.01152
GLU72	1.13714
LYS73	1.48579

ASP74	1.45933
ASN75	1.00231
TRP76	0.945053
ILE77	0.835164
LYS78	0.975089
VAL79	0.856831
THR80	1.14848
ASP81	1.1546
ASN82	1.3827
GLY83	1.33091
ARG84	1.62906
GLY85	1.22755
ILE86	1.23272
PRO87	1.27548
VAL88	1.1888
ASP89	1.58673
ILE90	1.65714
GLN91	2.03359
GLU92	2.83175
LYS93	2.86108
MET94	2.42162
GLY95	1.97471
ARG96	1.70637
PRO97	1.28074
ALA98	1.19468
VAL99	0.934219
GLU100	1.14837
VAL101	1.09365
ILE102	1.00449
LEU103	0.900802
THR104	0.913154

SER128	0.996848
SER129	0.786923
VAL130	0.682032
VAL131	0.572771
ASN132	0.574021
ALA133	0.505257
LEU134	0.531947
SER135	0.520666
GLN136	0.821672
ASP137	0.959457
LEU138	0.582953
GLU139	0.846062
VAL140	0.614441
TYR141	0.785383
VAL142	0.840033
HIS143	1.14932
ARG144	1.21282
ASN145	1.31972
GLU146	1.34585
THR147	0.896509
ILE148	0.869876
TYR149	0.886648
HIS150	0.682573
GLN151	0.751421
ALA152	0.65634
TYR153	0.622571
LYS154	1.18787
LYS155	1.31097
GLY156	0.87642
VAL157	0.797059
PRO158	0.777656

GLN159	1.0354
PHE160	0.938851
ASP161	1.33225
LEU162	1.00029
LYS163	1.22011
GLU164	1.2744
VAL165	0.996324
GLY166	0.979366
THR167	1.20672
THR168	1.19475
ASP169	2.02288
LYS170	1.92374
THR171	1.37008
GLY172	1.01959
THR173	0.919041
VAL174	0.813485
ILE175	0.854326
ARG176	0.889323
PHE177	0.733571
LYS178	0.809691
ALA179	0.584761
ASP180	0.734625
GLY181	0.791905
GLU182	1.16447
ILE183	0.78263
PHE184	0.764848
THR185	1.298
GLU186	1.70805
THR187	1.6404
THR188	1.00261
VAL189	1.22646

TYR190	0.860777
ASN191	1.12736
TYR192	1.32788
GLU193	1.42527
THR194	1.02121
LEU195	1.09503
GLN196	1.5467
GLN197	1.88167
ARG198	1.82948
ILE199	1.39698
ARG200	2.13281
GLU201	2.02099
LEU202	1.69897
ALA203	2.00429
PHE204	2.76122
LEU205	2.52653
ASN206	2.34695
LYS207	2.22874
GLY208	2.14405
ILE209	1.93754
GLN210	1.93762
ILE211	1.40057
THR212	1.43977
LEU213	1.30391
ARG214	1.80569
ASP215	1.30307
GLU216	1.59546
ARG217	1.32037
ASP218	1.99201
GLU219	2.69883
GLU220	2.79416

ASN221	2.58134
VAL222	3.82339
ARG223	5.18321
GLU224	6.52276
ASP225	6.08563
SER226	6.19587
TYR227	5.8525
HIS228	5.9927
TYR229	4.8556
GLU230	4.2951

Table S8. RMSF of the best conformation pose of compound **7a** docked in the protein active site of PDB ID: 3TTZ.

Item	RMSF
TYR14	0.13379
GLY15	0.11579
ALA16	0.16112
GLY17	0.18376
GLN18	0.11248
ILE19	0.1942
GLN20	0.16847
VAL21	0.13273
LEU22	0.16069
GLU23	0.13334
GLY24	0.19896
LEU25	0.19059
GLU26	0.10816
ALA27	0.10324
VAL28	0.12659
ARG29	0.15319
LYS30	0.1598
ARG31	0.17427
PRO32	0.07168
GLY33	0.18152
MET34	0.04806
TYR35	0.1202
ILE36	0.16219
GLY37	0.1772
SER38	0.15911
THR39	0.11125
SER40	0.19427
GLU41	0.11606

ARG42	0.10917
GLY43	0.1595
LEU44	0.17582
HIS45	0.1796
HIS46	0.19154
LEU47	0.18199
VAL48	0.10698
TRP49	0.12063
GLU50	0.10402
ILE51	0.04884
VAL52	0.1326
ASP53	0.14869
ASN54	0.08625
SER55	0.01539
ILE56	0.11546
ASP57	0.02167
GLU58	0.10859
ALA59	0.17448
LEU60	0.13595
ALA61	0.07057
GLY62	0.05565
TYR63	0.15278
ALA64	0.01175
ASN65	0.12223
GLN66	0.12661
ILE67	0.10245
GLU68	0.1292
VAL69	0.17159
VAL70	0.1241
ILE71	0.15701
GLU72	0.10972

LYS73	0.03787
ASP74	0.02562
ASN75	0.12966
TRP76	0.11344
ILE77	0.18932
LYS78	0.04817
VAL79	0.03172
THR80	0.05116
ASP81	0.086
ASN82	0.06377
GLY83	0.16127
ARG84	0.06
GLY85	0.10039
ILE86	0.14011
PRO87	0.13892
VAL88	0.15023
ASP89	0.11166
ILE90	0.12914
GLN91	0.14326
GLU92	0.16945
LYS93	0.16136
MET94	0.18154
GLY95	0.16513
ARG96	0.10162
PRO97	0.11724
ALA98	0.11375
VAL99	0.1478
GLU100	0.14248
VAL101	0.19748
ILE102	0.16708
LEU103	0.14311

THR104	0.13694
SER128	0.1469
SER129	0.14994
VAL130	0.16251
VAL131	0.13267
ASN132	0.17767
ALA133	0.15416
LEU134	0.14241
SER135	0.19409
GLN136	0.12665
ASP137	0.17652
LEU138	0.17436
GLU139	0.10126
VAL140	0.14195
TYR141	0.13242
VAL142	0.17324
HIS143	0.11364
ARG144	0.02554
ASN145	0.11709
GLU146	0.1785
THR147	0.11273
ILE148	0.1437
TYR149	0.11615
HIS150	0.16444
GLN151	0.19203
ALA152	0.15164
TYR153	0.11061
LYS154	0.10982
LYS155	0.13967
GLY156	0.19907
VAL157	0.19586

PRO158	0.15144
GLN159	0.15231
PHE160	0.19762
ASP161	0.10544
LEU162	0.1987
LYS163	0.13013
GLU164	0.15055
VAL165	0.14108
GLY166	0.12736
THR167	0.18751
THR168	0.17919
ASP169	0.19564
LYS170	0.12226
THR171	0.14483
GLY172	0.13359
THR173	0.10267
VAL174	0.19744
ILE175	0.16797
ARG176	0.17752
PHE177	0.08615
LYS178	0.11054
ALA179	0.06677
ASP180	0.14084
GLY181	0.07161
GLU182	0.17295
ILE183	0.07815
PHE184	0.17806
THR185	0.13596
GLU186	0.10428
THR187	0.1219
THR188	0.11833

VAL189	0.10345
TYR190	0.03763
ASN191	0.03573
TYR192	0.13679
GLU193	0.13277
THR194	0.11567
LEU195	0.11036
GLN196	0.16334
GLN197	0.13357
ARG198	0.17125
ILE199	0.09158
ARG200	0.10068
GLU201	0.08432
LEU202	0.11363
ALA203	0.15175
PHE204	0.12383
LEU205	0.0369
ASN206	0.14351
LYS207	0.03452
GLY208	0.04831
ILE209	0.09889
GLN210	0.1856
ILE211	0.16005
THR212	0.08144
LEU213	0.1327
ARG214	0.13363
ASP215	0.16684
GLU216	0.10355
ARG217	0.12625
ASP218	0.09811
GLU219	0.09774

GLU220	0.10227
ASN221	0.08376
VAL222	0.14647
ARG223	0.10691
GLU224	0.09994
ASP225	0.10598
SER226	0.14927
TYR227	0.0999
HIS228	0.06
TYR229	0.14478
GLU230	0.08562

Table S9. RMSF of the best conformation pose of compound **7l** docked in the protein active site of PDB ID: 3TTZ.

Item	RMSF
TYR14	0.08787
GLY15	0.15739
ALA16	0.15342
GLY17	0.07352
GLN18	0.06822
ILE19	0.07183
GLN20	0.11619
VAL21	0.11139
LEU22	0.13691
GLU23	0.10048
GLY24	0.12472
LEU25	0.12525
GLU26	0.11526
ALA27	0.14045
VAL28	0.07268
ARG29	0.12156
LYS30	0.14134
ARG31	0.15262
PRO32	0.10002
GLY33	0.09853
MET34	0.11176
TYR35	0.09298
ILE36	0.08458
GLY37	0.12131
SER38	0.09441
THR39	0.06134
SER40	0.07504
GLU41	0.12339

ARG42	0.05692
GLY43	0.07164
LEU44	0.09275
HIS45	0.13594
HIS46	0.06449
LEU47	0.07678
VAL48	0.05142
TRP49	0.05319
GLU50	0.15663
ILE51	0.12529
VAL52	0.1656
ASP53	0.15642
ASN54	0.10688
SER55	0.16658
ILE56	0.16089
ASP57	0.07647
GLU58	0.06452
ALA59	0.07719
LEU60	0.05769
ALA61	0.12229
GLY62	0.07599
TYR63	0.07266
ALA64	0.11694
ASN65	0.13804
GLN66	0.09363
ILE67	0.0826
GLU68	0.05976
VAL69	0.0655
VAL70	0.05297
ILE71	0.06424
GLU72	0.10001

LYS73	0.13936
ASP74	0.14873
ASN75	0.15561
TRP76	0.13432
ILE77	0.06637
LYS78	0.08269
VAL79	0.08949
THR80	0.07986
ASP81	0.02584
ASN82	0.12
GLY83	0.11398
ARG84	0.13569
GLY85	0.04753
ILE86	0.02315
PRO87	0.04047
VAL88	0.06194
ASP89	0.07964
ILE90	0.04159
GLN91	0.18007
GLU92	0.02666
LYS93	0.19357
MET94	0.17674
GLY95	0.14568
ARG96	0.10722
PRO97	0.16499
ALA98	0.17127
VAL99	0.14243
GLU100	0.16957
VAL101	0.17141
ILE102	0.08474
LEU103	0.13234

THR104	0.17154
SER128	0.16648
SER129	0.1441
VAL130	0.17266
VAL131	0.10635
ASN132	0.11833
ALA133	0.13424
LEU134	0.09639
SER135	0.1483
GLN136	0.11464
ASP137	0.08885
LEU138	0.07139
GLU139	0.13636
VAL140	0.11272
TYR141	0.16561
VAL142	0.14142
HIS143	0.10708
ARG144	0.14825
ASN145	0.11304
GLU146	0.16639
THR147	0.16872
ILE148	0.09813
TYR149	0.07525
HIS150	0.13856
GLN151	0.18126
ALA152	0.11347
TYR153	0.18544
LYS154	0.16586
LYS155	0.09025
GLY156	0.0948
VAL157	0.08907

PRO158	0.1642
GLN159	0.14456
PHE160	0.15189
ASP161	0.10135
LEU162	0.07018
LYS163	0.09238
GLU164	0.10119
VAL165	0.06006
GLY166	0.14064
THR167	0.10853
THR168	0.10923
ASP169	0.07155
LYS170	0.09625
THR171	0.07097
GLY172	0.08239
THR173	0.10018
VAL174	0.15753
ILE175	0.17087
ARG176	0.12825
PHE177	0.13821
LYS178	0.15154
ALA179	0.10862
ASP180	0.14129
GLY181	0.08207
GLU182	0.05113
ILE183	0.08351
PHE184	0.11689
THR185	0.09944
GLU186	0.08686
THR187	0.18592
THR188	0.14615

VAL189	0.16748
TYR190	0.14978
ASN191	0.12822
TYR192	0.15038
GLU193	0.08467
THR194	0.06562
LEU195	0.13803
GLN196	0.08477
GLN197	0.04602
ARG198	0.08472
ILE199	0.04927
ARG200	0.07088
GLU201	0.12633
LEU202	0.08247
ALA203	0.05862
PHE204	0.0418
LEU205	0.12435
ASN206	0.08562
LYS207	0.06044
GLY208	0.10824
ILE209	0.04652
GLN210	0.06474
ILE211	0.16236
THR212	0.15573
LEU213	0.15508
ARG214	0.10114
ASP215	0.18361
GLU216	0.14439
ARG217	0.10895
ASP218	0.05489
GLU219	0.11304

GLU220	0.10517
ASN221	0.09749
VAL222	0.06377
ARG223	0.10664
GLU224	0.13367
ASP225	0.09093
SER226	0.08546
TYR227	0.03609
HIS228	0.08951
TYR229	0.01374
GLU230	0.15414

Table S10. Physicochemical Space of curcumin hybrids K_1-K_{14}. The pink region indicates the ideal physicochemical space for oral bioavailability.

7a	7b	7c
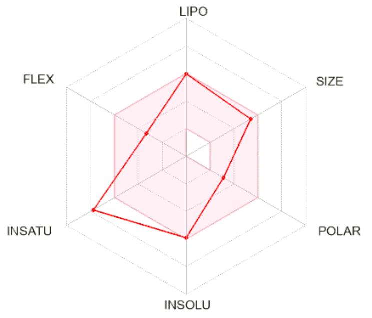	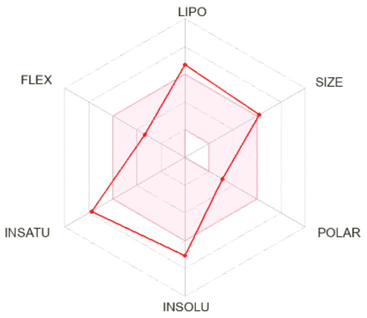	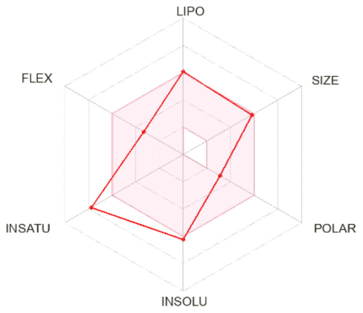
**7d**	**7e**	**7f**
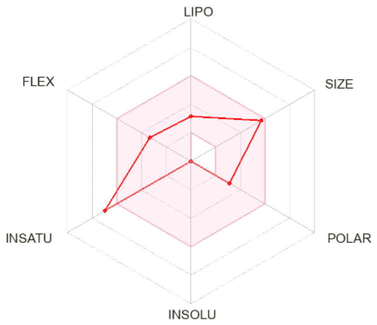	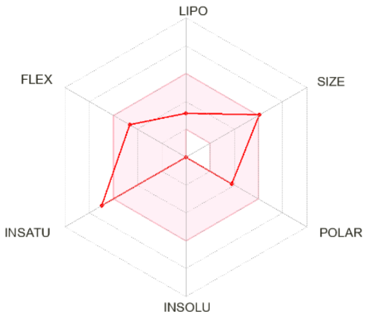	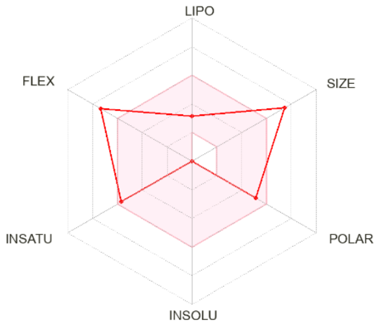
**7g**	**7h**	**7i**

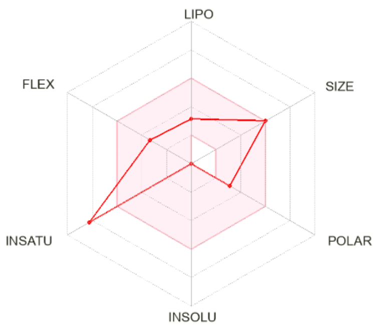
LIPO
SIZE
POLAR
INSOLU
INSATU
FLEX

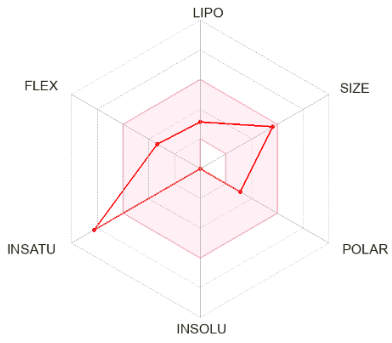
LIPO
SIZE
POLAR
INSOLU
INSATU
FLEX

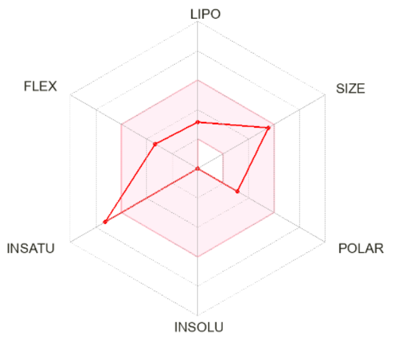
LIPO
SIZE
POLAR
INSOLU
INSATU
FLEX

7j	7k	7l

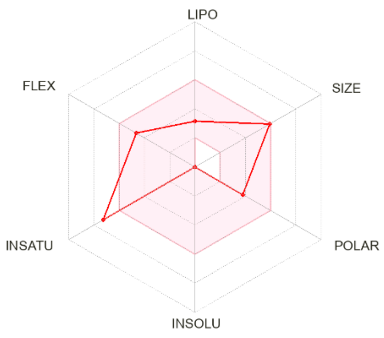
LIPO
SIZE
POLAR
INSOLU
INSATU
FLEX

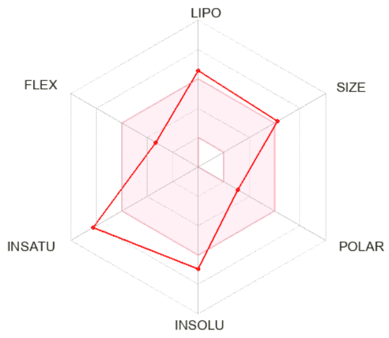
LIPO
SIZE
POLAR
INSOLU
INSATU
FLEX

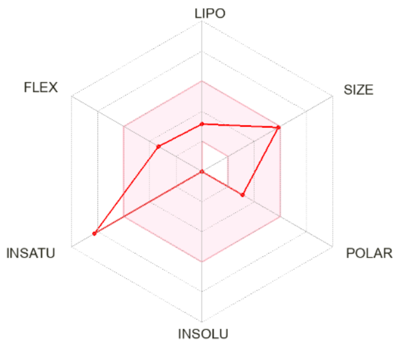
LIPO
SIZE
POLAR
INSOLU
INSATU
FLEX

7m	7n	

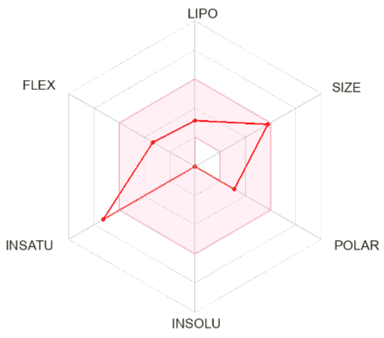
LIPO
SIZE
POLAR
INSOLU
INSATU
FLEX

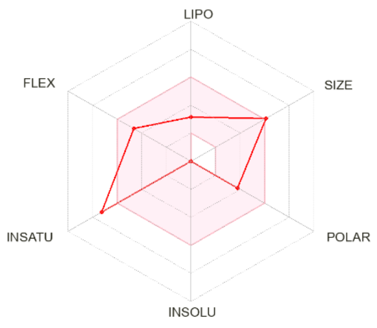
LIPO
SIZE
POLAR
INSOLU
INSATU
FLEX
